# Neoadjuvant PD‐1 Blockade Tislelizumab (BGB‐A317) in Combination With Etoposide and Platinum‐Based Chemotherapy for Limited‐Disease Small Cell Lung Cancer

**DOI:** 10.1002/mco2.70972

**Published:** 2026-09-03

**Authors:** Shiyue Wan, Shiqi Hu, Yilv Yan, Fenghuan Sun, Dongliang Bian, Jie Luo, Dong Xie, Wenxin He, Liang Duan, Yuming Zhu, Nan Song, Ming Liu, Hongcheng Liu, Gening Jiang, Peng Zhang

**Affiliations:** ^1^ Department of Thoracic Surgery Shanghai Pulmonary Hospital Tongji University School of Medicine Shanghai China; ^2^ Department of Oncology Shanghai Pulmonary Hospital Tongji University School of Medicine Shanghai China

**Keywords:** bioinformatics, immunochemotherapy, neoadjuvant therapy, small cell lung cancer

## Abstract

Neoadjuvant immunochemotherapy has transformed treatment paradigms in non–small cell lung cancer, yet its applicability in limited‐disease small cell lung cancer (LD‐SCLC) remains unknown. Here, we report findings from LungMate‐006, the first prospective Phase II trial evaluating PD‐1 blockade with tislelizumab combined with platinum–etoposide in LD‐SCLC. Among 15 enrolled patients, the regimen achieved an objective response rate of 66.7% and enabled curative‐intent surgery in 60% of patients, all with R0 resection. Major pathological response and pathological complete response occurred in 44.4% and 33.3% of surgical cases, respectively. Median event‐free and overall survival reached 21.7 and 32.7 months. Metabolic response by PET‐CT more accurately predicted pathological remission than radiographic shrinkage alone. Bulk RNA sequencing revealed a treatment‐induced transition from a metabolically active baseline state toward an extracellular matrix–remodeled, CAF‐ and macrophage‐enriched microenvironment, alongside upregulation of genes associated with tumor stemness and immune evasion. These transcriptomic changes suggest stromal–myeloid–mediated adaptive resistance in non‐MPR tumors and highlight potential avenues for refining perioperative immunochemotherapy strategies in SCLC.

## Graphical abstract

This study assessed neoadjuvant PD‐1 blockade plus chemotherapy in patients with resectable limited‐stage small cell lung cancer. Peri‐treatment biopsy and surgical samples were analyzed using bulk RNA sequencing and bioinformatics approaches. The results support the feasibility of this perioperative strategy and reveal treatment‐associated transcriptomic and immune microenvironment remodeling, providing molecular insights for future optimization.

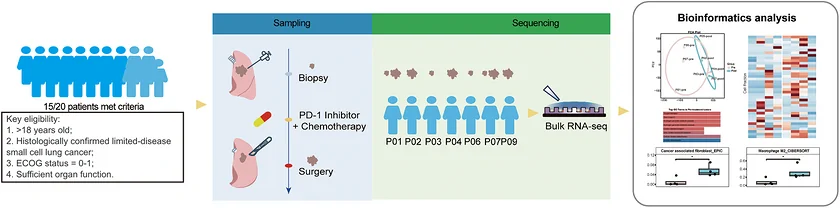

## 1 Introduction

Lung cancer continues to be one of the most prevalent and deadly forms of cancer both in China and worldwide [1, 2]. Among the various types of lung cancer, small cell lung cancer (SCLC) accounts for approximately 15% of all cases. SCLC is particularly aggressive, characterized by rapid growth and early metastasis to other parts of the body. Unfortunately, by the time most patients are diagnosed, approximately 70% are already at an advanced stage of the disease [3]. Despite more than 40 Phase III clinical trials and over 60 drugs being tested, none have shown significant success in improving outcomes for SCLC patients [4, 5].

One promising avenue of research has been the use of immune checkpoint blockade (ICBs). The results of several multicenter randomized double‐blind Phase III studies (CASPIAN and Impower133) showed that combining the PD‐L1 antibody durvalumab with platinum and etoposide significantly enhanced both overall survival (OS) and progression‐free survival (PFS) in ED‐SCLC [6, 7]. Recently, the RATIONALE‐312 trial demonstrated that the addition of PD‐1 blockade to etoposide plus platinum chemotherapy significantly improved survival in extensive‐disease SCLC (ED‐SCLC) compared to chemotherapy alone [8]. Furthermore, the ADRIATIC trial showed that PD‐L1 inhibitor consolidation following concurrent or sequential chemoradiotherapy markedly prolonged OS and progression‐free survival (PFS) in patients with limited‐disease SCLC (LD‐SCLC) [9, 10, 11]. These findings underscored the potential of combining ICBs with topical therapies for LD‐SCLC. However, the safety and efficacy of combining surgery with PD‐1 blockade in neoadjuvant settings for untreated LD‐SCLC remain largely unexplored [12].

In the field of non‐small cell lung cancer (NSCLC), advances in diagnostic techniques, perioperative systemic therapy, and surgical innovations have collectively expanded the boundaries of thoracic surgery, resulting in higher rates of radical resection and improved disease‐free survival. Inspired by these achievements, our study aimed to investigate whether a similar perioperative immunochemotherapy strategy could be translated into the treatment paradigm of resectable limited‐disease SCLC.

This study is a prospective, single‐arm Phase II clinical trial (LungMate‐006) designed to evaluate the feasibility and safety of neoadjuvant immunochemotherapy combining PD‐1 blockade with tislelizumab and etoposide and platinum‐based chemotherapy in patients with LD‐SCLC. The primary endpoints were predefined as feasibility—measured by treatment compliance and surgical conversion rate—and safety—assessed by treatment‐related adverse events. The study met its primary endpoints, demonstrating an objective response rate (ORR) of 66.7% and a disease control rate (DCR) of 86.7%, with no unexpected or intolerable adverse events, thereby supporting the clinical feasibility and tolerability of this neoadjuvant approach. Additionally, the study investigates the molecular features and transcriptomic alterations of SCLC by performing bulk RNA sequencing on baseline and post‐treatment tumor samples to characterize treatment‐induced changes within the tumor and its microenvironment.

## 2 Results

### 2.1 Cohort Characteristics

**FIGURE 1:**
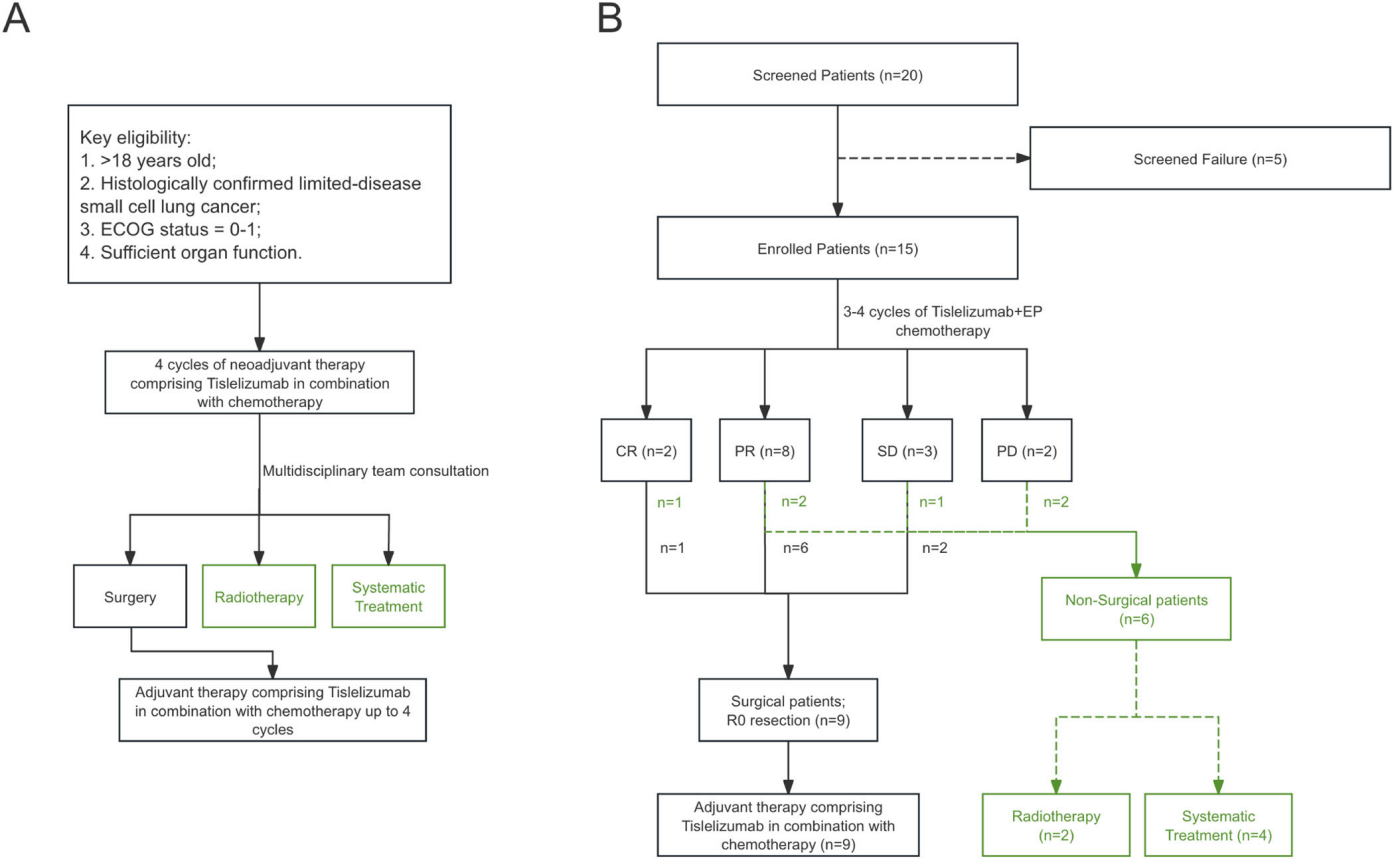
Study design and clinical efficacy of the LungMate‐006 trial. (A) Schematic representation of the study design and treatment protocols. Patients with histologically confirmed limited‐disease small cell lung cancer (LD‐SCLC) meeting key eligibility criteria, including ECOG performance status ≤1 and sufficient organ function, underwent four cycles of neoadjuvant therapy comprising tislelizumab in combination with platinum‐based double‐agent chemotherapy (etoposide and platinum [EP] regimen). Following treatment, patients were evaluated for curative‐intent surgery or radiotherapy. Post‐surgical patients received adjuvant therapy comprising tislelizumab combined with EP chemotherapy. (B) Flowchart summarizing patient screening, enrollment, clinical responses, and treatment allocation. Among 20 screened patients, 15 were enrolled and received neoadjuvant therapy. Clinical responses included complete response (CR; *n* = 2, 13.3%), partial response (PR; *n* = 8, 53.4%), stable disease (SD; *n* = 3, 20.0%), and progressive disease (PD; *n* = 2, 13.3%). Nine patients (60%) underwent surgery, all achieving R0 resection. Non‐surgical patients (*n* = 6) received either radiotherapy or systemic therapy based on clinical evaluation and patient preference. Among the 20 patients screened, five were excluded due to failure to meet eligibility criteria or other clinical considerations. Detailed reasons are provided in Supporting Information.

Twenty eligible patients with LD‐SCLC underwent screening between July 15, 2021, and December 29, 2023, with 15 of them subsequently enrolled in this study as an intention‐to‐treat (ITT) population, as depicted in Figure 1A and 1B. The baseline characteristics were presented in Table 1. The median age at diagnosis was 64 years 64 [interquartile range (IQR): 59–67], with the majority of participants being male (13/15, 86.7%), smokers (14/15, 93.3%), and having an ECOG status of 0 (14/15, 93.3%). Among the enrolled participants, 13 (86.7%) were diagnosed with pure SCLC, while two (13.3%) were diagnosed with combined SCLC—specifically, Patient P01 with combined SCLC‐squamous cell carcinoma and Patient P09 with combined SCLC‐adenocarcinoma. The median maximum tumor dimension of the primary tumor (MTD) for all participants was 32.1 mm [IQR: 24.4–57.7]. The majority of participants (11/15, 73.3%) were categorized as Stage N2. Among the enrolled participants, six (40.0%) were categorized as Stage IIIA, seven (46.6%) as Stage IIIB, one (6.7%) as Stage IIB, and one (6.7%) as Stage IA3.

**TABLE 1:** Baseline characteristics of SCLC patients who received neoadjuvant immunochemotherapy (*n* = 15).

Variables	No. of patients (%)
**Age, median [IQR]**	64 [59–67]
**Gender**	
Male	13 (86.7)
Female	2 (13.3)
**ECOG performance status**	
0	14 (93.3)
1	1 (6.7)
**Histology**	
Pure SCLC	13 (86.7)
Combined SCLC	2 (13.3)
**Smoking history**	
Ever	14 (93.3)
Never	1 (6.7)
**MTD, mm, median [IQR]**	32.1 [24.4–57.7]
**N stage at diagnosis**	
0	1 (6.7)
1	3 (20.0)
2	11 (73.3)
**cTNM stage at diagnosis**	
IA3	1 (6.7)
IIB	1 (6.7)
IIIA	6 (40.0)
IIIB	7 (46.6)

Abbreviations: MTD, maximum tumor dimension; SCLC, small cell lung cancer.

### 2.2 Efficacy

**FIGURE 2:**
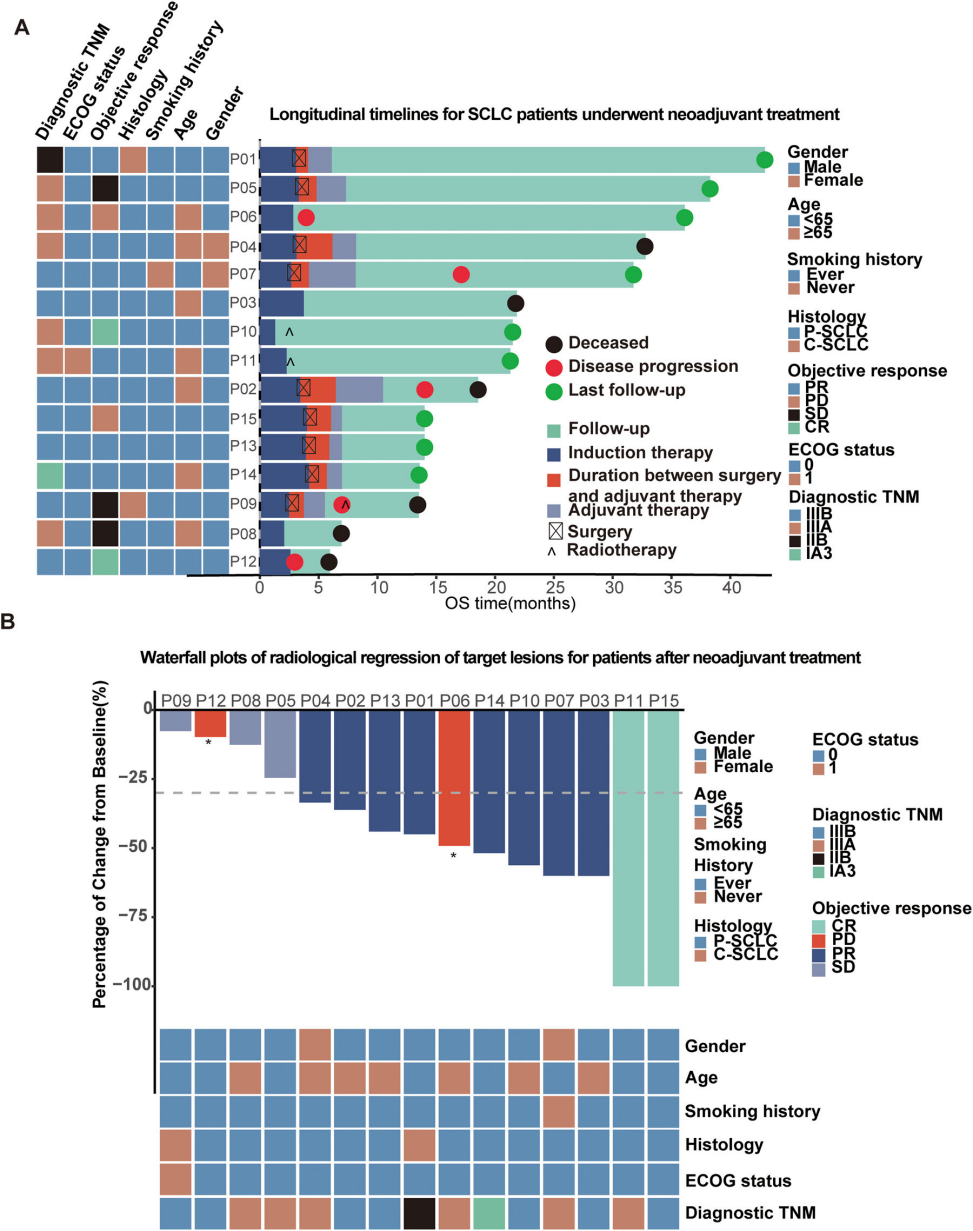
Longitudinal timelines and radiological outcomes of participants treated with neoadjuvant tislelizumab plus chemotherapy. (A) Swimming plot illustrating clinical characteristics, treatment timelines, and follow‐up details of the intention‐to‐treat (ITT) population (*n* = 15). Each bar represents an individual patient, categorized by clinical response to neoadjuvant therapy (CR: complete response; PR: partial response; SD: stable disease; PD: progressive disease). Clinical attributes including gender, age, smoking history, histology (pure small cell lung cancer [P‐SCLC] or combined small cell lung cancer [C‐SCLC]), ECOG performance status, and diagnostic tumor‐node‐metastasis (TNM) staging are displayed alongside the longitudinal treatment outcomes. (B) Waterfall plot showing the percentage change in the radiological size of target lesions from baseline following neoadjuvant treatment, as assessed by RECIST 1.1 criteria. Bars are color‐coded based on individual patient characteristics and treatment outcomes, with patients achieving radiological progression (PD) or non‐progression (SD, PR, CR) clearly delineated. *: Patient P06 was confirmed to have progressive disease due to bone metastasis, while Patient P12 experienced liver metastasis. *Post‐treatment details*: Following disease progression, Patient P02 received chemotherapy with nab‐paclitaxel after developing brain metastasis. Patient P04 completed protocol‐defined adjuvant therapy and transitioned to maintenance therapy with traditional Chinese medicine. Patient P06, who developed bone metastasis, was treated with anlotinib and denosumab for maintenance. Patient P07 experienced relapse in the seventh lymph node group and subsequently underwent one cycle of tislelizumab with cisplatin–etoposide, followed by three cycles of tislelizumab combined with nab‐paclitaxel. Patient P08, deemed inoperable after induction therapy, received two cycles of adebrelimab plus nab‐paclitaxel. Patient P09, following disease progression, was treated with concurrent chemoradiotherapy and a maintenance regimen of nab‐paclitaxel, anlotinib, and irinotecan. Patient P11 underwent radiotherapy and was subsequently maintained on traditional Chinese medicine. Finally, Patient P12 received one cycle of paclitaxel liposome for progressive disease. *Abbreviations*: NAT, neoadjuvant therapy; ECOG, Eastern Cooperative Oncology Group performance status; CR, complete response; PR, partial response; SD, stable disease; PD, progressive disease; RECIST, Response Evaluation Criteria in Solid Tumors.

The efficacy data for neoadjuvant tislelizumab plus chemotherapy are presented in Table 2. Following neoadjuvant treatment (median cycles = 4, IQR: 4–4), eight patients (53.4%) achieved a partial response (PR), two patients (13.3%) achieved a complete response (CR), three patients (20.0%) had stable disease (SD), and two patients (13.3%) experienced progressive disease (PD). The ORR was 66.7% (95% confidence interval [CI]: 38.4%–88.2%), and the DCR was 86.7% (95% CI: 59.5%–98.3%). Swimming plot and waterfall plot illustrating the treatment details and graphic response outcomes of the ITT population are depicted in Figures 2A and 2B, respectively. The surgery rate among patients who underwent neoadjuvant tislelizumab plus chemotherapy was 60% (9/15). These treatment decisions reflect a combination of factors, including disease progression, clinical assessment of treatment response, patient preference, and feasibility of curative‐intent surgery.

**TABLE 2:** Evaluation of efficacy of neoadjuvant immunochemotherapy.

Variables	No. of patients (%)
**Induction ICT cycles, median [IQR]**	4 [4–4]
**Surgery performed**	9 (60.0)
**Objective response**	
PR	8 (53.4)
CR	2 (13.3)
SD	3 (20.0)
PD	2 (13.3)
ORR (%)	66.7
DCR (%)	86.7
**MTD regression, median% [IQR]**	45.00 [29.04–58.10]
**ypTNM** **^a^**	
0	3 (33.3)
IA	2 (22.2)
IIIA	3 (33.3)
IIIB	1 (11.2)
**Extent of resection** **^a^**	
R0	9 (100)
**Surgical methods** **^a^**	
VATS	5 (55.6)
RATS	2 (22.2)
Thoracotomy	2 (22.2)
**Extent of surgery** **^a^**	
Sublobectomy	1 (11.1)
Sleeve lobectomy	1 (11.1)
Lobectomy	7 (77.3)
**Residual tumor cell, median% [IQR]** **^a^**	40.0 [0.0–70.0]
**Pathological response** **^a^**	
pCR	3 (33.3)
MPR	1 (11.1)
Non‐MPR	5 (55.6)
**Adjuvant ICT cycles, median [IQR]** **^a^**	4 [2–4]
**Median operation time, min [IQR]** **^a^**	185.0 [140.0–204.0]
**Median blood loss, mL [IQR]** **^a^**	100.0 [50.0–100.0]
**Median hospital stays postoperatively, days [IQR]** **^a^**	5.0 [4.0–6.0]
**Median chest drainage time, days [IQR]** **^a^**	5.0 [4.0–6.0]

Abbreviations: CR, complete response; DCR, disease control rate; ICT, immunochemotherapy; MPR, major pathologic response; MTD, maximum tumor dimension; ORR, objective response rate; pCR, pathological complete response; PD, progressive disease; PR, partial response; RATS, robot‐assisted thoracic surgery; SD, stable disease; VATS, video‐assisted thoracic surgery.

^a^ Patients received neoadjuvant therapy followed by surgery.

The MPR rate for the ITT population was 26.7% (4/15, 95% CI: 7.8%–55.1%). The MPR rate for the surgical population was 44.4% (4/9, 95% CI: 13.7%–78.8%). Among the four participants who achieved MPR, three participants achieved a pathologic complete response (pCR). All surgical patients achieved R0 resection following neoadjuvant tislelizumab plus chemotherapy. Furthermore, the median percentage of residual tumor cells in the primary tumor after surgery was 40.0% [IQR: 0.0%–70%]. The median duration of postoperative hospital stay was 5 days [IQR: 4–6], and chest drainage was maintained for a median of 5 days [IQR: 4–6]. The median operation time during surgery was 185.0 min [IQR: 140.0–204.0], with a median blood loss of 100.0 mL [IQR: 50.0–100.0]. All surgical patients underwent adjuvant therapy comprising tislelizumab in combination with chemotherapy (median cycles = 4, IQR: 2–4).

### 2.3 Survival Analysis

**FIGURE 3:**
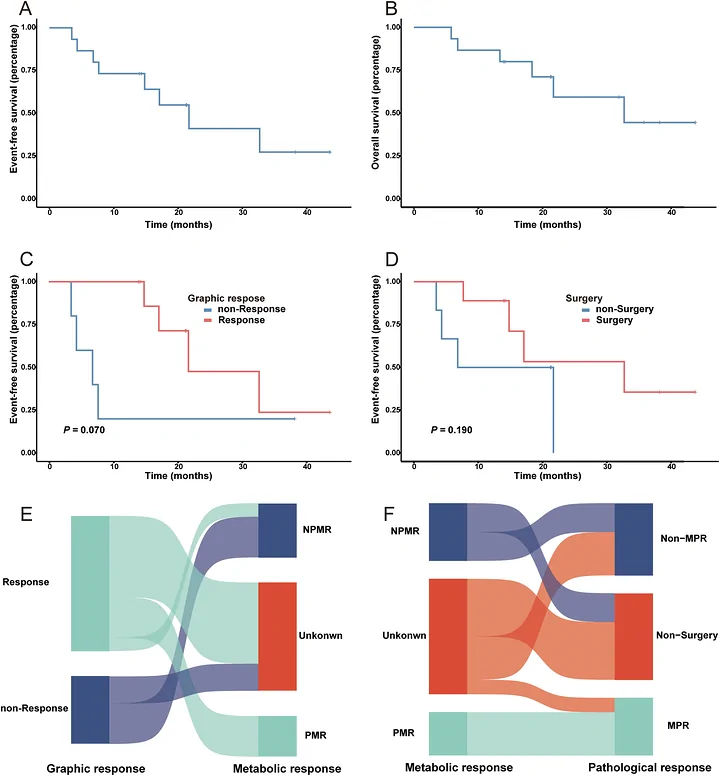
Exploratory outcomes evaluating survival and response associations in the intention‐to‐treat (ITT) population. (A) Kaplan–Meier curve depicting event‐free survival (EFS) in the ITT population (*n* = 15). (B) Kaplan–Meier curve illustrating overall survival (OS) in the ITT population (*n* = 15). (C) Kaplan–Meier curve comparing EFS between participants with a graphic response (partial response [PR] or complete response [CR]) and those with a non‐graphic response (stable disease [SD] or progressive disease [PD]). Patients achieving a graphic response exhibited a trend toward improved EFS compared to non‐responders (*p* = 0.070). (D) Kaplan–Meier curve comparing EFS between patients who underwent surgery and those who did not. Although patients in the surgery group showed a trend toward prolonged EFS compared to the non‐surgery group, the difference was not statistically significant (*p* = 0.190). (E) Sankey diagram showing the association between graphic response and metabolic response. Patients with graphic responses were more likely to achieve a partial metabolic response (PMR) or complete metabolic response (CMR). (F) Sankey diagram illustrating the relationship between metabolic response and pathological response. Participants achieving PMR were more likely to exhibit a major pathological response (MPR), while non‐major pathological response (NMPR) was observed in those with stable metabolic disease (SMD). *Abbreviations*: PR, partial response; CR, complete response; SD, stable disease; PD, progressive disease; PMR, partial metabolic response; CMR, complete metabolic response; MPR, major pathological response; NMPR, non‐major pathological response; NE, not estimable.

As of the data cutoff on February 17, 2025, the median follow‐up duration for all participants was 31.9 months [IQR: 21.2–38.2 months]. During the follow‐up period, five patients experienced disease recurrence or metastasis, and six patients died. The median event‐free survival (EFS) for the ITT population was 21.7 months (95% CI: 14.8 to not estimable [NE]), as depicted in Figure 3A. The median OS for the ITT population was 32.7 months (95% CI: 21.7 to NE), as illustrated in Figure 3B. The 12‐month EFS rate was 73.3% (95% CI: 54.0%–95.5%), and the 12‐month OS rate was 86.7% (95% CI: 71.1%–100.0%). The 24‐month EFS rate was 41.3% (95% CI: 19.3%–88.0%), and the 24‐month OS rate was 59.3% (95% CI: 36.1%–97.3%). In the surgical population, the median EFS was 32.7 months (95% CI: 14.8 to NE), and the median OS was also 32.7 months (95% CI: 18.4 to NE), as shown in Figures S1A and S1B, respectively. The 12‐month EFS rate of the surgical population was 88.9% (95% CI: 0.6%–100%), and the 12‐month OS rate was 100% (95% CI: 100%–100%). Compared with participants who failed to achieve a radiographic response (SD or PD), those who achieved a partial or complete response (PR or CR) during the neoadjuvant induction period demonstrated prolonged EFS (median EFS: 21.7 vs. 6.8 months; *p* = 0.070), as shown in Figure 3C and Table S1. Furthermore, as shown in Figure 3D and Table S2, among the nine patients who underwent surgery following neoadjuvant therapy, the median EFS was 32.7 months, compared with 14.2 months in those who did not undergo surgery (*p* = 0.190). We further performed exploratory analyses stratified by pathological response status. No statistically significant differences in EFS or OS were observed between patients with pCR/MPR and those with non‐MPR (*p* = 0.17 and *p* = 0.22), which may be attributable to the limited sample size and follow‐up duration (Figure S2). Notably, no recurrence or progression events have been observed in patients achieving pCR to date.

### 2.4 Safety

Treatment‐related adverse events (TRAEs) are presented in Table 3. All participants (15/15, 100%) experienced AEs, and no Grade 5 AEs were observed. Grade 3/4 AEs were observed in 11 (11/15, 73.3%) participants, most notably neutropenia (11/11, 100%). Other Grade 3/4 AEs included anemia, decreased white blood cell counts, decreased platelets, and immune‐related hyponatremia. No patients discontinued treatment with tislelizumab combined with chemotherapy due to TRAEs. Eight (8/15, 53.3%) patients experienced immune‐related adverse events, including hyponatremia, altered thyroid function, hyperkalemia, and hyperglycemia. Grade ≥3 immune‐related AEs were observed in only one patient, presenting as hyponatremia. Anemia was present in all participants, and blood count alterations were common. Leukopenia and neutropenia were observed in 14/15 (93.3%) patients, and platelet reduction in 13/15 (86.7%) patients. One patient experienced postoperative complications, specifically postoperative hemothorax, but recovered through non‐surgical procedures. No surgery‐related deaths occurred within 90 days postoperatively.

**TABLE 3:** Adverse events in neoadjuvant immunochemotherapy and surgery.

Type of events	All grade *n* (%)	Grade 1–2 *n* (%)	Grade ≥3 *n* (%)
**Treatment‐related adverse event**			
Rash	1 (6.7)	1 (6.7)	0
Alopecia	12 (80.0)	12 (80.0)	0
Nausea	6 (40.0)	6 (40.0)	0
Decreased appetite	9 (60.0)	9 (60.0)	0
Vomiting	2 (13.3)	2 (13.3)	0
Constipation	1 (6.7)	1 (6.7)	0
fever	1 (6.7)	1 (6.7)	0
Anemia	15 (100)	14 (93.3)	1 (6.7)
WBC count decreased	14 (93.3)	13 (86.7)	1 (6.7)
Neutropenia	14 (93.3)	3 (20.0)	11 (73.3)
Thrombocytopenia	13 (86.7)	11 (73.3)	2 (13.3)
ALT increased	4 (26.7)	4 (26.7)	0
AST increased	3 (20.0)	3 (20.0)	0
**Immune‐related adverse events**			
Hyponatremia	5 (33.3)	4 (26.7)	1 (6.7)
Hyperthyroidism/Hypothyroidism	2 (13.3)	2 (13.3)	0
Hyperkalemia	1 (6.7)	1 (6.7)	0
Hyperglycemia	3 (20.0)	3 (20.0)	0

Abbreviations: ALT, alanine aminotransferase; AST, aspartate aminotransferase; WBC, white blood cells.

### 2.5 Metabolic Responses

Metabolic responses of seven (46.7%) to the investigational therapy were evaluated in the current cohort using positron emission tomography/computed tomography with the tracer [18F]‐fluorodeoxyglucose (FDG‐PET/CT). In the present cohort of seven patients, the metabolic response rate, as per the Positron Emission Tomography Response Criteria in Solid Tumors (PERCIST), was 42.9% (3/7), as detailed in Table S3. Patients achieving either partial metabolic response (PMR) or complete metabolic response (CMR) invariably achieved a partial response (PR), yet among the four patients achieving PR/CR, only three exhibited PMR/CMR (Figure 3E). Furthermore, all patients demonstrating a pCR also exhibited CMR or PMR (Figure 3F).

### 2.6 Bulk RNA‐seq Reveals a Metabolic‐to‐ECM Transition and Myeloid–Stromal Remodeling After Neoadjuvant PD‐1–Based Immunochemotherapy

**FIGURE 4:**
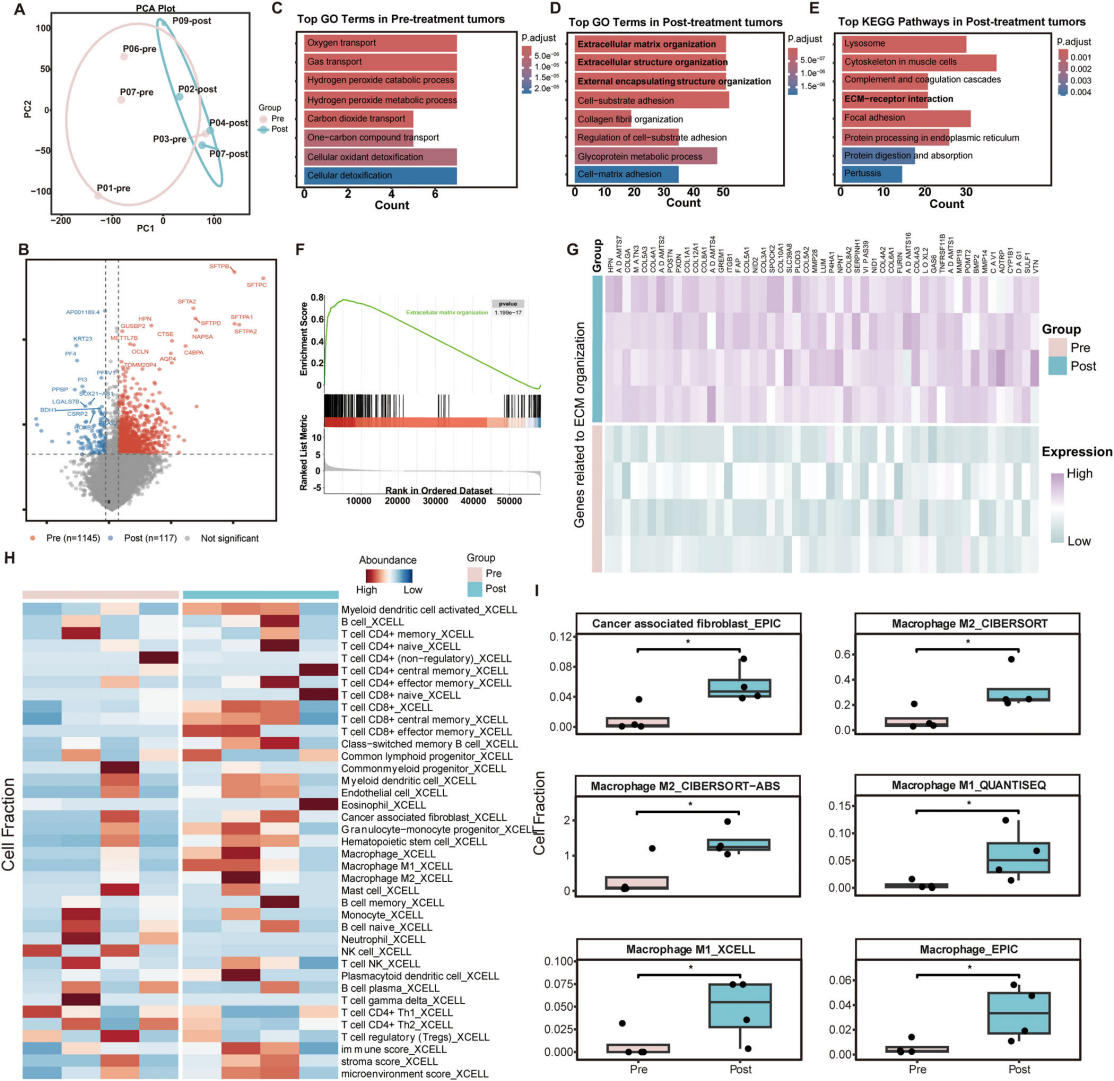
Bulk RNA‐seq analysis reveals metabolic‐to‐ECM transcriptional transition and stromal–myeloid remodeling after neoadjuvant PD‐1–based immunochemotherapy. (A) Principal component analysis (PCA) of paired pre‐ and post‐treatment small cell lung cancer (SCLC) tumor samples showing clear separation of transcriptomic profiles following neoadjuvant PD‐1 blockade plus platinum–etoposide. (B) Volcano plot displaying differentially expressed genes between pre‐ and post‐treatment tumors. Red dots indicate genes upregulated pre‐treatment, blue dots indicate genes upregulated post‐treatment, and gray dots denote non‐significant genes. Key genes associated with tumor stemness, plasticity, and immune tolerance (e.g., SOX21‐AS1, KRT23, BDH1) are highlighted. (C) Top Gene Ontology (GO) biological processes enriched in pre‐treatment tumors, showing dominant oxidative and metabolic signatures characteristic of proliferative SCLC. (D) Top GO terms enriched in post‐treatment tumors, revealing strong activation of extracellular matrix (ECM)–related processes, including ECM organization and collagen fibril formation. (E) Top KEGG pathways enriched in post‐treatment tumors, highlighting ECM–receptor interaction, focal adhesion, lysosomal programs, and cytoskeletal remodeling. (F) Representative GSEA plot showing significant enrichment of ECM‐associated pathways in post‐treatment tumors. (G) Heatmap of ECM‐organization–related genes demonstrating coordinated upregulation of collagens and matrix‐modifying enzymes (e.g., COL1A1, COL4A1, MMP2, ITGB1) following treatment. (H) Immune cell deconvolution (XCell) showing broad remodeling of the tumor microenvironment after therapy across lymphoid, myeloid, and stromal compartments. (I) Multiple independent deconvolution algorithms (EPIC, CIBERSORT, CIBERSORT‐ABS, QUANTISEQ, XCell) consistently reveal increased fractions of cancer‐associated fibroblasts (CAFs) and both M1 and M2 macrophages post‐treatment, indicating stromal expansion and myeloid recruitment. *p*‐values from paired statistical comparisons are indicated.

To delineate transcriptional alterations induced by neoadjuvant PD‐1 blockade plus platinum–etoposide, we performed bulk RNA sequencing on paired pre‐ and post‐treatment tumors (Table S4). Principal component analysis demonstrated a clear separation between the two groups, indicating substantial transcriptomic reprogramming following therapy (Figure 4A). Differential expression analysis identified 1145 genes upregulated in pre‐treatment tumors and 117 genes elevated post‐treatment (Figure 4B). Notably, post‐treatment samples showed increased expression of SOX21‐AS1, KRT23, BDH1, LGALS7B, and CSRNP2, genes associated with tumor stemness, epithelial plasticity, metabolic adaptation, and immune tolerance. As all bulk RNA‐seq samples were derived from non‐MPR cases, this profile may reflect early adaptive resistance to combined immunochemotherapy.

Functional enrichment revealed distinct biological states before and after therapy. Pre‐treatment tumors were enriched for oxidative and gas‐transport processes—including oxygen, carbon dioxide, and hydrogen peroxide metabolism—consistent with the high‐metabolic, highly proliferative phenotype of untreated SCLC (Figure 4C). In contrast, post‐treatment tumors displayed marked enrichment of extracellular matrix (ECM)–related programs, including ECM organization, extracellular structure remodeling, collagen fibril organization, and cell–substrate adhesion (Figure 4D). KEGG analysis further highlighted activation of ECM–receptor interaction, focal adhesion, lysosomal function, and cytoskeletal pathways (Figure 4E), collectively indicating that surviving tumor cells adopt an ECM‐dependent survival state.

Gene‐level analysis corroborated these findings: a broad set of ECM‐organization genes—including multiple collagens (COL1A1, COL1A2, COL4A1, COL4A2, COL8A1, COL5A3), matrix enzymes (MMP2, PXDN), and stromal regulators (ITGB1, SPOCK2, GREM1)—were uniformly upregulated after therapy (Figure 4G). This coordinated induction suggests that residual tumors remodel their structural niche to withstand immune and chemotherapeutic pressure.

Given the strong ECM signatures, we next assessed changes in the tumor microenvironment. XCell analysis demonstrated pronounced remodeling across immune and stromal compartments after treatment (Figure 4H). Multiple independent algorithms—including EPIC, CIBERSORT, CIBERSORT‐ABS, QUANTISEQ, and XCell—consistently revealed significant increases in cancer‐associated fibroblasts (CAFs) as well as both M1 and M2 macrophage subsets (Figure 4I). The expansion of CAFs aligns with the observed ECM remodeling, while macrophage accumulation—across methodological platforms—suggests enhanced myeloid engagement and potential paracrine crosstalk with stromal and tumor cells. Parallel to this myeloid–stromal remodeling, repertoire reconstruction using TRUST4 revealed a functional expansion of the adaptive immune compartment. Notably, post‐treatment tumors exhibited an augmented immune landscape, highlighted by a significant increase in B‐cell receptor (BCR) clonotype richness, suggesting a concurrent antigen‐driven lymphocyte response despite the non‐MPR status (Figure S3A,B).

Collectively, these results reveal that neoadjuvant PD‐1–based immunochemotherapy drives a coordinated metabolic‐to‐ECM transcriptional shift and induces myeloid–stromal co‐evolution, characterized by ECM reinforcement, CAF expansion, and macrophage recruitment. Such remodeling may facilitate persistence of non‐MPR tumors and represents a potential axis of therapeutic resistance in SCLC.

## 3 Discussion

LungMate‐006 is the first reported cohort to explore the safety and feasibility of neoadjuvant PD‐1 blockade for LD‐SCLC. The primary endpoint of this study was to evaluate the safety and feasibility of tislelizumab in combination with chemotherapy for the treatment of LD‐SCLC. Our results suggested that this induction regimen achieved a favorable DCR (86.75) and ORR (66.7%). The 12‐month EFS rate and OS rate were 71.1% and 86.7%, respectively. Furthermore, patients who achieved a significant treatment response in graphic or underwent surgery after induction therapy experienced long‐term survival benefits, with adverse events remaining within acceptable limits.

Although the exploration of PD‐L1 blockade in the treatment of SCLC has advanced rapidly, with PD‐L1 blockade combined with chemotherapy becoming the standard of care for ED‐SCLC, the development of PD‐1 blockade has faced significant challenges [13]. Both second‐ and first‐line trials of PD‐1 blockade have encountered difficulties, underscoring the complexities of using PD‐1 inhibitors in SCLC treatment. Moreover, studies on neoadjuvant PD‐1 blockade for LD‐SCLC are scarce. However, the success of consolidation therapy with durvalumab following CCRT suggests that combining immune checkpoint blockade with topical therapies could be valuable for treating LD‐SCLC. This study is the first to provide data on the safety and efficacy of PD‐1 blockade as a neoadjuvant treatment for LD‐SCLC, with the results indicating that tislelizumab in combination with chemotherapy is both safe and effective for this purpose. A small proportion of patients had combined SCLC histology, which may introduce heterogeneity, although its impact is likely minimal given the limited number of cases.

Current guidelines do not recommend surgery as a treatment option for N+SCLC. However, in this cohort, patients achieved an ORR of 66.7% and a DCR of 86.7%. Another study investigating PD‐L1 blockade in combination with chemotherapy for LD‐SCLC reported an objective response rate (ORR) of 94.1% [12]. In our cohort, 60% of patients (9/15) underwent successful surgery, while two of 15 received radiotherapy. While a Phase II study reported surgical rates of 26%, 16%, and 10% in patients with Stage IIIA, IIIB, and IIIC SCLC, respectively, following treatment with SHR‐1701, with or without chemotherapy [14]. These findings suggest that neoadjuvant sequential surgery or radiotherapy combined with the PD‐1 plus EP regimen may be a feasible treatment option for patients with LD‐SCLC. This neoadjuvant strategy should not be considered an alternative to upfront surgery, but rather a conversion approach that may enable selected patients with initially unresectable LD‐SCLC to undergo subsequent surgical resection.

Moreover, a previous study reported a pathological complete response (pCR) rate of 19% following neoadjuvant chemotherapy in patients with LD‐SCLC [15]. In contrast, neoadjuvant PD‐L1 blockade combined with chemotherapy achieved a pCR rate of 47.1% in a similar population [12]. In our cohort, the MPR rate among those who underwent surgery was 44.4% (4/9), and the pCR rate was 33.3% (3/9). This represents a significant improvement over chemotherapy alone, suggesting that the addition of PD‐1 blockade may substantially enhance tumor eradication in SCLC, offering a potential benefit to patients.

Survival outcomes are crucial for evaluating the efficacy of perioperative tislelizumab combined with chemotherapy. In this study, tislelizumab combined with chemotherapy as a neoadjuvant treatment demonstrated promising survival rates, with a 12‐month EFS rate of 73.3%, a 12‐month OS rate of 86.7%, a median EFS of 21.7 months, and a median OS of 32.7 months in the ITT population. Comparatively, in the JCOG9104 and CONVERT studies, patients with LD‐SCLC who received chemotherapy combined with CRT had 12‐month PFS rates ranging from 50% to 75% [16, 17]. The recently published ADRIATIC study demonstrated significant survival improvements for patients with LS‐SCLC, with 12‐month PFS and OS rates of approximately 52% and 85%, respectively, following durvalumab consolidation therapy post‐chemoradiotherapy [18]. Our findings suggest that the neoadjuvant regimen of tislelizumab combined with platinum‐based chemotherapy achieved comparable efficacy to that reported in the ADRIATIC trial. It is important to note that in the present study, non‐surgical patients did not receive consolidation immunotherapy following radiotherapy. Furthermore, although our results suggest favorable clinical outcomes with neoadjuvant tislelizumab combined with chemotherapy, direct comparisons with the ADRIATIC trial should be interpreted with caution due to differences in study design, patient selection, and treatment strategies.

However, further extended follow‐up will be required to fully assess the long‐term survival benefits of this neoadjuvant approach in our cohort. In a Phase 1/2 trial, 83% of Stage III patients treated with pembrolizumab and CCRT had a 12‐month PFS of 65% [10]. Notably, the current study suggests that replacing radiotherapy with surgery as a local treatment option, combined with PD‐1 blockade induction therapy, may provide greater survival benefits. In contrast, our study reported a 12‐month OS rate of 86.7% in the ITT population and 100% in the surgical population, supporting the feasibility of surgery following induction with tislelizumab combined with chemotherapy.

Moreover, patients tolerated neoadjuvant tislelizumab combination chemotherapy well, with manageable safety profiles, as evidenced by no treatment discontinuation due to TRAEs. Although Grade 3–4 adverse events were frequent, they were mainly hematologic and consistent with EP chemotherapy. No patients were excluded from surgery, and no surgical delays or increased perioperative complications were observed, suggesting that the addition of tislelizumab did not compromise tolerability [6, 19, 20, 21].

Furthermore, analysis of the exploratory endpoints in the current study suggests that the significance of PET/CT in perioperative evaluation of small cell lung cancer patients may have been underestimated. Previous studies in lung cancer, particularly in the context of immunotherapy, have suggested that metabolic response assessed by PET/CT may better reflect treatment efficacy compared to conventional radiological evaluation. However, whether these findings can be extrapolated to LD‐SCLC remains to be determined [22]. Throughout four cycles of neoadjuvant therapy, most patients attained PR, yet only those achieving both partial metabolic response (PMR) and PR ultimately reached MPR, whereas those with PR and stable metabolic disease (SMD) were confirmed non‐MPR pathologically. These findings suggest that metabolic response may provide additional information beyond radiological assessment in evaluating treatment response. However, given the limited sample size, this observation should be considered exploratory and requires validation in larger prospective studies.

Bulk RNA‐seq profiling revealed pronounced transcriptional remodeling in both tumor cells and the tumor microenvironment following neoadjuvant therapy. Several genes—SOX21‐AS1, KRT23, BDH1, LGALS7B, and CSRNP2—were markedly upregulated after PD‐1 blockade combined with EP chemotherapy. These genes have been implicated in tumor stemness, treatment resistance [23, 24], and immune evasion across multiple cancer types, suggesting that their induction may contribute to therapeutic tolerance in SCLC.

At baseline, untreated SCLC samples were enriched for metabolic and transport pathways, consistent with the highly proliferative, metabolically active phenotype characteristic of this disease. In contrast, post‐treatment tumors showed strong enrichment of extracellular matrix (ECM) remodeling pathways. This shift implies that ECM reorganization may represent a key adaptive mechanism through which SCLC cells evade cytotoxic or immune‐mediated killing [25, 26, 27].

Analysis of tumor microenvironmental cell populations further supported this model. Cancer‐associated fibroblasts—central regulators of ECM architecture—were significantly expanded after therapy. Concurrently, both M1 and M2 macrophage populations increased across multiple deconvolution algorithms. The coordinated rise of CAFs and myeloid populations suggests a stromal–myeloid axis that reinforces ECM remodeling [28], thereby providing structural and paracrine support that enables residual SCLC cells to survive treatment.

The enrichment of ECM components, CAFs, and macrophages in non‐MPR cases highlights critical therapeutic vulnerabilities [29]. While this fibrotic TME likely acts as a barrier to treatment in SCLC [30], the biological plasticity of these stromal cells suggests that their resistance‐conferring roles may be reversible [31]. Strategies such as “normalizing” the stroma via TGF‐β or FAP inhibition, combined with myeloid‐reprogramming agents, offer promising avenues to re‐sensitize resistant tumors to immunochemotherapy [32, 33]. Future validation in patient‐derived models will be essential to evaluate whether targeting this ECM‐CAF‐macrophage axis can effectively overcome resistance and improve clinical outcomes [34].

Collectively, these findings highlight ECM‐driven and myeloid–stromal–mediated mechanisms as potential contributors to resistance against neoadjuvant immunochemotherapy in SCLC.

Current guidelines recommend adjuvant chemotherapy and postoperative radiotherapy—particularly mediastinal—for N2‐stage LD‐SCLC to reduce locoregional recurrence. However, this study did not include postoperative radiotherapy, which may affect long‐term disease control. Second, although prophylactic cranial irradiation (PCI) is standard for patients with N2 involvement, it was omitted due to concerns over neurocognitive toxicity and patient preference. In addition, the absence of consolidation immunotherapy in non‐surgical patients reflects historical treatment practices and limits the generalizability of our findings to current standards of care. These factors may limit the generalizability of our findings. Future studies should consider incorporating thoracic radiotherapy and PCI to better evaluate surgery‐based multimodal strategies in LD‐SCLC.

## 4 Conclusion

Neoadjuvant PD‐1 blockade with tislelizumab combined with platinum–etoposide shows acceptable safety and feasibility in patients with LD‐SCLC, enabling surgical resection in a substantial proportion of cases and providing encouraging survival outcomes. Bulk transcriptomic analyses reveal that tumors undergoing this regimen transition from a metabolically active baseline state to an ECM‐remodeled, CAF‐ and macrophage‐enriched microenvironment, suggesting stromal–myeloid–mediated adaptive resistance in non‐MPR tumors. These findings underscore the need to integrate tumor‐intrinsic features and microenvironmental remodeling into future strategies for perioperative immunochemotherapy in SCLC and highlight potential therapeutic avenues targeting ECM‐ and myeloid‐associated resistance pathways.

## 5 Materials and Methods

### 5.1 Study Design

LungMate‐006 is a single‐arm, open‐label, single‐center study of neoadjuvant tislelizumab in combination with conventional chemotherapy for the treatment of LD‐SCLC. This study was conducted at Shanghai Pulmonary Hospital, which was registered at https://www.clinicaltrials.gov before participant enrollment (NCT04542369). This study aimed to investigate the feasibility and safety of tislelizumab in combination with chemotherapy as a neoadjuvant treatment for resectable LD‐SCLC. The study protocol is provided in the Supporting Information.

### 5.2 Patient Eligibility

Treatment‐naïve patients with resectable LD‐SCLC, older than 18 years, with an Eastern Cooperative Oncology Group (ECOG) performance status of 0 or 1, were enrolled in this trial. At the time of diagnosis, two thoracic surgeons independently evaluated tumor resectability using positron emission tomography/computed tomography (PET/CT) and chest computed tomography (CT). Patients with extensive mediastinal nodal disease, including involvement of more than three nodal stations or bulky/conglomerated N2 lymph nodes, were generally considered unresectable and were not selected for surgical intervention.

SCLC was pathologically confirmed by immunohistochemical analysis of specimens obtained through endobronchial ultrasound‐guided biopsy (EBUS) or percutaneous lung biopsy. Clinical staging was determined using chest CT, brain magnetic resonance imaging (MRI), and PET/CT. Eligible patients were required to have adequate hematologic, hepatic, renal, and pulmonary function to tolerate neoadjuvant therapy followed by radical surgery.

Patients who were pregnant or breastfeeding, had unstable systemic disorders such as interstitial lung disease, pulmonary fibrosis, or cardiovascular disease, or had received any antitumor treatment outside the study protocol, including chemotherapy or immunotherapy, were excluded from this trial.

### 5.3 Treatment Protocols

All enrolled subjects underwent four cycles of treatment, administered once every 3 weeks, following the specified regimen for each cycle: (i) tislelizumab: 200 mg per dose, administered via intravenous infusion (30–60 min) on Day 1 of each cycle; (ii) platinum‐based double‐agent chemotherapy (etoposide and platinum regimen): cisplatin 75 mg/m^2^ or carboplatin (AUC5) plus etoposide 100 mg/m^2^, administered via intravenous infusion, with cisplatin/carboplatin on Day 1 of each cycle and etoposide on Days 1, 2, and 3. Patients deemed resectable at the conclusion of the neoadjuvant cycles were scheduled for surgery within 30 weeks of the initial dose of neoadjuvant therapy. If the participants received less than four cycles of neoadjuvant therapy, the patient may remain in the study and should undergo surgery within 4–8 weeks of the last study protocol treatment. After surgical resection, patients received adjuvant tislelizumab (200 mg) in combination with platinum‐based double‐agent chemotherapy once every 3 weeks for up to four cycles. Patients determined to be unsuitable for surgery following neoadjuvant therapy received treatment as assessed by their physician (Figure 1A). During neoadjuvant therapy, treatment was administered every 3 weeks. Radiographic evaluation with chest CT was routinely performed after every two cycles to assess tumor response. Additional imaging was conducted if disease progression was clinically suspected. PET/CT was recommended after completion of neoadjuvant therapy prior to surgery to evaluate systemic tumor burden. For patients who did not undergo PET/CT, alternative staging methods, including bone scintigraphy, brain MRI, and other organ‐specific imaging, were performed. Discontinuation of neoadjuvant therapy and transition to alternative treatment strategies (e.g., chemoradiotherapy or systemic therapy) were determined based on disease progression, treatment tolerance, and multidisciplinary team (MDT) evaluation.

### 5.4 Primary Endpoint

The primary endpoints of this study were to evaluate the feasibility and safety of neoadjuvant anti‐PD‐1 therapy in combination with etoposide and platinum‐based chemotherapy for the treatment of limited‐disease small cell lung cancer (LD‐SCLC). Feasibility was defined based on treatment compliance and the rate of successful surgical conversion, while safety was assessed by the incidence and severity of treatment‐related adverse events (TRAEs). Safety evaluations were conducted according to the Common Terminology Criteria for Adverse Events (CTCAE) version 4.0 and included documentation of perioperative complications.

### 5.5 Secondary Endpoint

Efficacy outcomes include the objective response rate (ORR) and disease control rate (DCR) assessed according to Response Evaluation Criteria in Solid Tumors version 1.1 (RECIST 1.1) for solid tumors; the major pathological response (MPR) of intention‐to‐treat (ITT) population defined as tumor cell presence of less than 10% or primary lesion disappearance with lymph node metastasis; pathological complete response (pCR) was defined as the absence of any viable tumor cells in both the primary tumor and sampled lymph nodes; the surgery rate for the ITT population; R0 resection rate within the surgical population; overall survival (OS) and event‐free survival (EFS). EFS was defined as the time from treatment initiation to the first occurrence of disease progression, failure to undergo surgery, postoperative recurrence, or death from any cause.

### 5.6 Exploratory Outcomes

Exploration of the interplay among ORR, metabolic response rates, and pathologic response status, alongside transcriptomic characterization of SCLC during treatment. Metabolic response rates were estimated after correcting for attenuation, following the Positron Emission Tomography Response Criteria in Solid Tumors version 1.0 (PERCIST 1.0). Patients underwent FDG‐PET/CT at baseline and after completion of neoadjuvant therapy (after four cycles), prior to surgery, to evaluate metabolic response and reassess resectability. The dynamic transcriptomic characterization was derived from bulk RNA‐seq analysis of paired pre‐ and post‐treatment tumor samples from enrolled patients.

### 5.7 Follow‐Up

Follow‐up was conducted via telephone contact or outpatient visits. For participants who underwent surgery, chest CT scans were performed every 3 months during the first year postoperatively, every 6 months from 2 to 5 years, and annually thereafter. Additionally, annual MRI scans of the brain, abdominal ultrasonography, and bone scans were performed. Follow‐up continued until the time of death as detailed in Supporting Information.

### 5.8 Multiplex Immunofluorescence Staining

Formalin‐fixed paraffin‐embedded (FFPE) tumor sections were subjected to multiplex immunofluorescence (mIF) staining using a four‐color immunohistochemistry kit (Absin, cat# abs50012‐20T) according to the manufacturer's protocol. Antigen retrieval was performed using citrate buffer (pH 6.0), followed by blocking and sequential primary antibody incubation. The following primary antibodies were used: anti‐Prosurfactant Protein C (SFTPC; Abcam, cat# ab90716; 1:200), anti‐NEUROD1 (Abcam, cat# ab109224; 1:150), and anti‐ASCL1 (BD Biosciences, cat# 556604; 1:200). Species‐specific HRP‐conjugated secondary antibodies and tyramide signal amplification (TSA) were applied sequentially for each target. Nuclei were counterstained with DAPI. Fluorescence images were acquired using a confocal microscope under consistent exposure settings across all samples.

### 5.9 Statistical Analysis

Efficacy and safety analyses were conducted on the ITT population, comprising patients who received the neoadjuvant treatment with tislelizumab in combination with chemotherapy according to the treatment protocol. All statistical analyses were performed using appropriate descriptive and inferential methods, including chi‐square or Fisher's exact tests for categorical variables and independent‐samples *t*‐tests for continuous variables. Survival outcomes were estimated using the Kaplan–Meier method, and between‐group comparisons were conducted with the log‐rank test. The median duration of follow‐up was estimated by the reverse Kaplan–Meier approach. EFS and OS were analyzed as key endpoints to evaluate the feasibility of neoadjuvant treatment. EFS was measured from the start of neoadjuvant therapy to the first occurrence of disease progression before or after surgery, postoperative recurrence, progression in patients who did not undergo surgery, or death from any cause. OS was calculated from the date of diagnosis until death from any cause.

### 5.10 RNA Sequencing and Bioinformatic Processing

A total of eight tissue samples, with four collected at baseline and four following treatment, were included in this study and successfully processed for RNA sequencing (RNA‐seq). Total RNA was isolated from fresh‐frozen specimens using TRIzol reagent. Library preparation was carried out with the NEBNext Ultra RNA Library Prep Kit for Illumina, and unique index adapters were ligated to each sample to enable multiplexing. Pooled libraries underwent paired‐end sequencing (2× 150 bp) on an Illumina NovaSeq 6000 platform.

Raw sequencing reads in FASTQ format were subjected to quality control and adapter trimming using fastp (version 0.20.1) [35]. The cleaned reads were then aligned to the human reference genome (GRCh38) with the splice‐aware aligner STAR (version 2.7.6a) [36], employing default parameters. Resulting BAM files were used as input for read quantification, which was performed by featureCounts (version 2.0.1) [36] to generate gene‐level count matrices. Expression levels were normalized using the trimmed mean of *M*‐values (TMM) method and subsequently transformed to log_2_(counts per million+1). To mitigate potential technical batch effects, the “ComBat” algorithm implemented in the sva R package was applied.

### 5.11 Differential Gene Expression Analysis

Differential expression between baseline and post‐treatment groups was assessed using DESeq2 [37]. This method models raw count data via a negative binomial distribution and estimates dispersion to identify statistically significant changes. For each gene, the fold change was computed to quantify the magnitude and direction of expression differences, while statistical significance was evaluated using a two‐sided Wald test. Genes meeting the thresholds of |log_2_(fold change)| greater than 1 and adjusted *p* < 0.05 were classified as differentially expressed. The T‐cell receptor and B‐cell receptor repertoires are constructed by the TRUST4 algorithm [38].

### 5.12 Gene Set Enrichment Analysis

To explore biological pathways associated with transcriptional changes, we performed gene set enrichment analysis (GSEA) [39]. Predefined gene sets from the Gene Ontology (GO) and Kyoto Encyclopedia of Genes and Genomes (KEGG) databases downloaded from the Molecular Signatures Database were used as reference collections. GSEA was conducted using the standard weighted Kolmogorov–Smirnov‐like statistic. Pathways with an adjusted *p*‐value less than 0.05 were considered significantly enriched.

### 5.13 Tumour Microenvironment Estimation

The infiltration of immune cells was evaluated by the algorithm “immunedeconv” (v2.1.3) [40].

## Author Contributions

**Shiyue Wan**: conceptualization, data curation, formal analysis, visualization, Writing – original draft, Writing – review and editing. **Shiqi Hu**: data curation, formal analysis, investigation, writing – original draft, writing – review and editing. **Yilv Yan**: data curation, methodology, visualization, writing – original draft, writing – review and editing. **Fenghuan Sun**: data curation, investigation, visualization, writing – original draft, writing – review and editing. **Dongliang Bian**: investigation, data curation, writing – review and editing. **Jie Luo**: methodology, formal analysis, writing – review and editing. **Dong Xie**: investigation, data curation, writing – review and editing. **Wenxin He**: investigation, validation, writing – review and editing. **Liang Duan**: methodology, validation, writing – review and editing. **Yuming Zhu**: data curation, validation, writing – review and editing. **Nan Song**: conceptualization, methodology, project administration, writing – review and editing. **Ming Liu**: conceptualization, project administration, resources, writing – review and editing. **Hongcheng Liu**: funding acquisition, project administration, resources, supervision, writing – review and editing. **Gening Jiang**: funding acquisition, resources, supervision, writing – review and editing. **Peng Zhang**: conceptualization, funding acquisition, project administration, resources, supervision, writing – review and editing. All authors have read and approved the final manuscript.

## Funding

This work was supported by the National Natural Science Foundation of China (Grant No. 82430053, 82125001, 82503832), the China Postdoctoral Science Foundation (Grant No. 2025M772336), the Shanghai Science and Technology Committee (Grant No.24SF1904500), Innovation Program of Shanghai Municipal Education Commission (Grant No. 2023ZKZD33), and Foundation of Shanghai Pulmonary Hospital (Grant No. LYRC202402, FKLY20004).

## Ethics Statement

This study was conducted in accordance with the Declaration of Helsinki (as revised in 2013). The study was approved by the independent ethics committee in Tongji University‐affiliated Shanghai Pulmonary Hospital (FK‐LYIB‐001). All participants provided written informed consent, and there was no payment for their participation.

## Conflicts of Interest

The authors declare no conflicts of interest.

## Supporting information




**Supporting File**: mco270972‐sup‐0001‐SuppMat.docx.

## Data Availability

Raw bulk RNA‐seq data have been deposited to the Genome Sequence Archive of the BIG Data Center at the Beijing Institute of Genomics, Chinese Academy of Science, under accession number HRA017482 (accessible at http://bigd.big.ac.cn/gsa‐human).
