# Supplementary material for: Neoadjuvant PD‐1 Blockade Tislelizumab (BGB‐A317) in Combination With Etoposide and Platinum‐Based Chemotherapy for Limited‐Disease Small Cell Lung Cancer
Source: MedComm (2020). 2026 Sep 3;7(9):e70972. doi: 10.1002/mco2.70972 (PMC13542703; doi:10.1002/mco2.70972)
Supplement: Supplementary file 1 [file MCO2-7-e70972-s001.docx]

**Supplementary Materials**

Neoadjuvant PD-1 blockade Tislelizumab (BGB-A317) in Combination with Etoposide and Platinum-based Chemotherapy for Limited-Disease Small Cell Lung Cancer

Authors & Institutions:

Shiyue Wan, PhD 1^#^, Shiqi Hu, MD 1^#^, Yilv Yan, PhD 1^#^, Fenghuan Sun, MD 1^#^, Dongliang Bian, MD 1,Jie Luo, MD 2, Dong Xie, MD 1, Wenxin He, MD 1, Liang Duan, MD 1, Yuming Zhu, MD 1, Nan Song, MD 1^*^, Ming Liu, MD 1^*^, Hongcheng Liu, MD 1^*^, Gening Jiang, MD 1^*^, Peng Zhang, MD 1^*^

\# Contributed equally to this work

* Corresponding author

1 Department of Thoracic Surgery, Shanghai Pulmonary Hospital, Tongji University School of Medicine, No. 507 Zhengmin Road, Shanghai, 200433, China.

2 Department of Oncology, Shanghai Pulmonary Hospital, Tongji University School of Medicine, No. 507 Zhengmin Road, Shanghai, 200433, China.

Corresponding author’s email:

Peng Zhang: [zhangpeng1121@tongji.edu.cn](mailto:zhangpeng1121@tongji.edu.cn)

Gening Jiang: geningjiang@tongji.edu.cn

Hongcheng Liu: lhcsz@163.com

Ming Liu: liu_ming@tongji.edu.cn

Nan Song: songnan@tongji.edu.cn

**The reasons for not undergoing surgery are as follows:**

**Patient P03** was unable to proceed with further treatment due to the COVID-19 pandemic, preventing timely surgery. **Patient P06** developed bone metastasis during treatment, resulting in disease progression (PD); as a result, neither surgery nor radiotherapy was performed. **Patient P08** was evaluated with stable disease (SD) after induction therapy and was deemed inoperable. The patient subsequently received two cycles of adebrelimab plus nab-paclitaxel but developed pneumonia during treatment and passed away in January 2023. **Patient P11** achieved a complete response (CR) following induction therapy and actively chose to undergo radiotherapy instead of surgery. **Patient P12** experienced disease progression (PD), with liver metastasis confirmed, and did not undergo surgery or radiotherapy. **Patient P10** actively requested radiotherapy as the preferred treatment option and, consequently, did not undergo surgery.

**Among the 20 patients screened for eligibility, 5 patients were excluded prior to enrollment for the following reasons:**

Excluded patient 1 withdrew consent during the screening period due to concerns regarding tolerance to chemotherapy.

Excluded patient 2 was found to have bilateral lung metastases based on PET/CT evaluation and was therefore deemed ineligible.

Excluded patient 3 was identified with bone metastases on bone scintigraphy and also had impaired renal function, which did not meet the inclusion criteria.

Excluded patient 4 was excluded due to the presence of brain metastases detected by cranial magnetic resonance imaging (MRI).

Excluded patient 5 was found to have vertebral metastasis on PET/CT evaluation and was therefore excluded.

Overall, the primary reasons for exclusion were the presence of distant metastases and failure to meet eligibility criteria.

**Sample Size Estimation and Endpoint Setting**

This was a prospective, single-arm, exploratory phase II study, with feasibility and safety as the primary endpoints. Given the exploratory nature of the study, no formal confirmatory sample size calculation was performed.

To provide supportive rationale for the planned sample size, an approximate survival-based estimation was conducted using historical data. Based on previous studies, the median progression-free survival (PFS) of concurrent chemoradiotherapy in LD-SCLC was assumed to be approximately 10.0 months. It was hypothesized that the investigational regimen could improve median PFS to 20.0 months (hazard ratio [HR] = 0.50) under an exponential survival model.

Under these assumptions, approximately 10 PFS events would be required for exploratory design purposes. Assuming an accrual period of 18 months and a minimum follow-up of 26 months after the last patient enrolled, the expected event rate was approximately 70%. After accounting for an additional 5% censoring rate, the required sample size was estimated to be approximately 15 patients.

This estimation was intended to support detection of a preliminary efficacy signal rather than formal hypothesis testing. Therefore, efficacy analyses in this study were primarily descriptive and exploratory.

**Table S1. Baseline Characteristics Between Different Responding Patient Groups for Neoadjuvant Tislelizumab plus chemotherapy**

| **Variables** | **Overall** | **PR** | **NPR** | ***p*** | |
| --- | --- | --- | --- | --- | --- |
| **Total** | 15 | 10 | 5 |  |  |
| **Age, median [IQR]** | 64.0 [59.0, 67.0] | 64.5 [61.0, 66.5] | 63.0 [57.0, 67.0] | 0.805 |  |
| **Gender** |  |  |  |  |  |
| Male | 13 | 8 | 5 | 0.283 |  |
| Female | 2 | 2 | 0 |  |  |
| **ECOG performance status** |  |  |  |  |  |
| 0 | 14 | 9 | 5 | 0.464 |  |
| 1 | 1 | 1 | 0 |  |  |
| **Histology** |  |  |  |  |  |
| Pure SCLC | 13 | 9 | 4 | 0.591 |  |
| Combined SCLC | 2 | 1 | 1 |  |  |
| **Smoking History** |  |  |  |  |  |
| Ever | 14 | 9 | 5 | 0.464 |  |
| Never | 1 | 1 | 0 |  |  |
| **MTD at diagnosis, median [IQR]** | 32.10 [24.35, 57.70] | 36.10 [25.17, 56.75] | 32.10 [24.00, 88.90] | 0.713 |  |
| **N stage at diagnosis** |  |  |  |  |  |
| 0 | 1 | 1 | 0 | 0.761 |  |
| 1 | 3 | 2 | 1 |  |  |
| 2 | 11 | 7 | 4 |  |  |

(IQR: Interquartile range; SCLC: Small cell lung cancer; MTD: Maximum tumor dimension; ECOG: Eastern Cooperative Oncology Group performance status; PR: PR: Graphic response group including the partial response and complete response; NPR: Non-graphic response group including the stable disease and progressive disease.)

**Table S2. Baseline Characteristics and Response Information for Neoadjuvant Tislelizumab plus chemotherapy between Surgical and Non-Surgical Patients**

| **Variables** | **Overall** | **Surgical** | **Non-surgical** | ***p*** |
| --- | --- | --- | --- | --- |
| **Total** | 15 | 9 | 6 |  |
| **Age, median [IQR]** | 64.00 [59.00, 67.00] | 60.00 [57.00, 65.00] | 66.00 [64.25, 67.75] | 0.058 |
| **Gender** |  |  |  |  |
| Male | 13 | 7 | 6 | 0.215 |
| Female | 2 | 2 | 0 |  |
| **ECOG performance status** |  |  |  |  |
| 0 | 14 | 8 | 6 | 0.398 |
| 1 | 1 | 1 | 0 |  |
| **Histology** |  |  |  |  |
| Pure SCLC | 13 | 7 | 6 | 0.215 |
| Combined SCLC | 2 | 2 | 0 |  |
| **Smoking History** |  |  |  |  |
| Ever | 14 | 8 | 6 | 0.398 |
| Never | 1 | 1 | 0 |  |
| **MTD at diagnosis, median [IQR]** | 32.10 [24.35, 57.70] | 30.80 [24.70, 54.20] | 44.85 [24.23, 65.93] | 0.906 |
| **N stage at diagnosis** |  |  |  |  |
| 0 | 1 | 1 | 0 | 0.446 |
| 1 | 3 | 1 | 2 |  |
| 2 | 11 | 7 | 4 |  |
| **RECIST 1.1** |  |  |  |  |
| CR | 2 | 1 | 1 | 0.274 |
| PD | 2 | 0 | 2 |  |
| PR | 8 | 6 | 2 |  |
| SD | 3 | 2 | 1 |  |

(IQR: Interquartile range; SCLC: Small cell lung cancer; MTD: Maximum tumor dimension; ECOG: Eastern Cooperative Oncology Group performance status; CR: Completely response; PR: Partial response; SD: Stable disease; PD: Progressive disease.)
**Table S3. Information on objective response, metabolic response and pathological response of 7 patients.**

| **Patient Number** | **RECIST1.1** | **PERCIST1.0** | **Pathological response** |
| --- | --- | --- | --- |
| P01 | PR | SMD | Non-MPR |
| P08 | SD | SMD | Non-Surgery |
| P09 | CR | SMD | Non-MPR |
| P12 | PD | PMD | Non-Surgery |
| P13 | PR | PMR | pCR |
| P14 | PR | PMR | pCR |
| P15 | CR | CMR | pCR |

(CR: Completely response; PR: Partial response; SD: Stable disease; PD: Progressive disease; SMD: Stable metabolic disease; PMR: Partial metabolic response; CMR: Complete metabolic response; PMD: Progressive metabolic disease; pCR: Pathological complete response; MPR: Major pathologic response.)

**Table S4. Baseline characteristics and treatment information of patients included in single-cell transcriptome analyses**

| Patient Number | Gender | TNM at Diagnosed | Neoadjuvant Cycles | RESICT 1.1 | Treatment after neoadjuvant | Postoperative Treatment | Sample Type |
| --- | --- | --- | --- | --- | --- | --- | --- |
| P01 | Male | T2bN1M0-IIB | 4 | PR | Surgery | Tislelizumab in combination with chemotherapy for 4 cycles | Surgical specimens |
| P02 | Male | T3N2M0-IIIB | 4 | PR | Surgery | Tislelizumab in combination with chemotherapy for 4 cycles | Pre-treatment and Surgical specimens |
| P04 | Female | T1cN2M0-IIIA | 4 | PR | Surgery | Tislelizumab in combination with chemotherapy for 4 cycles | Surgical specimens |
| P06 | Male | T3N1M0-IIIA | 4 | PD | Systematic Treatment | Systematic Treatment | Pre-treatment specimens |
| P07 | Female | T4N2M0-IIIB | 4 | PR | Surgery | Tislelizumab in combination with chemotherapy for 4 cycles | Surgical specimens |
| P09 | Male | T4N2M0-IIIB | 4 | SD | Surgery | Tislelizumab in combination with chemotherapy for 3 cycles | Pre-treatment and Surgical specimens |

**Figure S1.** **Kaplan-Meier survival curves of the surgical population in the trial.**

**(A)** Kaplan-Meier curve depicting event-free survival (EFS) in the surgical population (n = 15). **(B)** Kaplan-Meier curve illustrating overall survival (OS) in the surgical population (n = 15).

 **Figure S2.** Kaplan–Meier curves of survival outcomes stratified by pathological response.

**(A)** Event-free survival (EFS) in patients achieving pathological complete response (pCR) or major pathological response (MPR) versus those with non-MPR.

**(B)** overall survival (OS) in patients achieving pCR/MPR versus those with non-MPR.

**Figure S3. Changes in BCR and TCR repertoire following neoadjuvant treatment.**

**(A)** B-cell receptor (BCR) clonotype richness (unique clonotypes) and total BCR abundance (total counts) before and after treatment. A significant increase in BCR clonotype richness was observed after treatment (P = 0.029), while total BCR abundance showed a non-significant increasing trend (P = 0.057).

**(B)** T-cell receptor (TCR) clonotype richness and total TCR abundance before and after treatment. No statistically significant differences were observed (P = 0.24 and P = 0.17, respectively), although an increasing trend was noted.

Each dot represents an individual patient, and paired samples are connected by dashed lines. Statistical comparisons were performed using paired tests.

Neoadjuvant PD-1 blockade Tislelizumab (BGB-A317) in Combination with Etoposide and Platinum-based Chemotherapy for Resectable Limited-Disease Small Cell Lung Cancer (LD-SCLC)

A Single-Arm, Open-Label, Phase II Clinical Trial

Investigator: Professor Zhang Peng

Institution: Shanghai Pulmonary Hospital of Tongji University

Study Period: 2020.7-2023.12

Version: 2.0

Version Date: 2020.7.15

**Confidentiality Statement**

The information contained in this document, especially the unpublished data, is owned by the Sponsor of this study and is provided to you as confidential since you are considered as an investigator, potential investigator, or consultant for review by you, your research team and the independent ethics committee/institutional review committee. It must be made clear that such confidential information is not allowed to be disclosed to others without the written approval from the Sponsor, except that it is used to obtain the Informed Consent Form from patients who will possibly take the study drugs.

| Protocol Summary | |
| --- | --- |
| Title | Neoadjuvant PD-1 blockade Tislelizumab (BGB-A317) in Combination with Etoposide and Platinum-based Chemotherapy for Resectable Limited-Disease Small Cell Lung Cancer (LD-SCLC): A Single-Arm, Open-Label, Phase II Clinical Trial |
| Version No. | 2 |
| Research Institution | Shanghai Pulmonary Hospital of Tongji University |
| Organization on in Charge | Shanghai Pulmonary Hospital of Tongji University |
| Principal Investigator | Professor Zhang Peng |
| Participants | Patients with Limited- disease stage small cell lung cancer |
| Objectives | To investigate the feasibility and safety of Tislelizumab in combination with chemotherapy as a neoadjuvant treatment for LD-SCLC |
| Study Design | Single-Arm, Open-Label, Phase II Clinical Trial |
| Sample Size | 15 in total |
| Test Drug | Tislelizumab (BGB-A317) |
| Dosage and Mode of Administration | 1. Tislelizumab: 200 mg/administration, intravenous infusion (30-60 minutes) on day 1 of each cycle; 2. Platinum-based doublet chemotherapy (etoposide and platinum regimen): cisplatin 75 mg/m² or carboplatin (AUC5) + etoposide 100 mg/m², intravenous infusion. Cisplatin/carboplatin is administered on day 1, etoposide on days 1, 2, and 3 of each cycle. |
| Trial Content | ①Preoperative Treatment Stage All enrolled patients will receive four treatment cycles, each lasting 3 weeks, with the following regimen: 1. Tislelizumab: 200 mg/administration, intravenous infusion (30-60 minutes) on day 1 of each cycle; 2. Platinum-based doublet chemotherapy (etoposide and platinum regimen): cisplatin 75 mg/m² or carboplatin (AUC5) + etoposide 100 mg/m², intravenous infusion. Cisplatin/carboplatin is administered on day 1, etoposide on days 1, 2, and 3 of each cycle. ②Surgical Treatment Stage The maximum interval from the first dose of neoadjuvant therapy to surgery is 30 weeks. Patients receiving fewer than 4 cycles of neoadjuvant therapy may continue in the study and should undergo surgery within 4-8 weeks after the last study treatment. Surgery must achieve non-palliative complete resection with negative margins. ③Postoperative Adjuvant Treatment Stage Postoperative adjuvant chemoimmunotherapy is given up to 4 cycles. Adjuvant radiotherapy may be added based on physician recommendation and patient preference, and treatment may be received at the study site or external facilities. |
| Research Time | 2020.7-2023.12 |
| Endpoints | ①The primary endpoints of this study are to observe the safety and feasibility of neoadjuvant anti-PD-1 therapy combined with etoposide and platinum in treating LD-SCLC.  ②Secondary endpoints include major pathological response rate, 5-year overall survival (5-y OS), progression-free survival (PFS), overall survival (OS), quality of life (QoL), and R0 resection rate. ③Exploratory endpoints: To explore potential predictive biomarkers in blood and tumor tissue, and to analyze lymphocyte infiltration in the tumor. |
| Statistical methods | Efficacy and safety analyses were conducted on the ITT population, comprising patients who received 4 cycles of neoadjuvant treatment with Tislelizumab in combination with chemotherapy. Descriptive statistics, chi-square tests, Fisher’s exact tests, independent sample t-tests, Kaplan-Meier method, and Log-Rank tests were employed for analysis. The median follow-up time was determined using the reverse Kaplan-Meier method. EFS and OS were calculated and reported to assess the feasibility of neoadjuvant treatment. EFS was defined as the time from the initiation of neoadjuvant therapy to disease progression before or after surgery, disease recurrence post-surgery, disease progression without surgery, or death from any cause. OS was defined as the time from the date of diagnosis to death from any cause. |
| Follow-up | Follow-up includes symptom monitoring, chest CT scans, whole-body ECT if bone pain is present, and brain MRI for symptoms like headache, vomiting, or gait disturbances. Postoperative follow-up occurs at 3 months, then every 6 months for 2 years if no recurrence or metastasis is detected. After 2 years without recurrence, follow-up transitions to telephone survival follow-up every 3 months ±7 days. For patients with recurrence or metastasis, follow-up switches to post-progression survival follow-up every 3 months ±14 days. |

Catalogue

[1. Background and Rationale	10](#_Toc172804876)

[1.1 Lung Cancer	10](#_Toc172804877)

[1.2 Small Cell Lung Cancer	10](#_Toc172804878)

[1.3 Current Treatment of Limited-Disease Small Cell Lung Cancer	10](#_Toc172804879)

[1.4 Tumor Immunotherapy	12](#_Toc172804880)

[1.5 Immunotherapy Target: PD-1	12](#_Toc172804881)

[1.6 Anti-PD-1 Drug: Tislelizumab (BGB-A317)	13](#_Toc172804882)

[1.7 Current Status of PD-1 Pathway-Targeted Therapy for SCLC Patients	13](#_Toc172804883)

[1.8 Research Rationale	14](#_Toc172804884)

[2. Research Objectives	15](#_Toc172804885)

[3. Study Design	15](#_Toc172804886)

[3.1 Study Design	15](#_Toc172804887)

[3.2 Sample Size	16](#_Toc172804888)

[3.3 Participant Selection	16](#_Toc172804889)

[3.3.1 Inclusion Criteria	16](#_Toc172804890)

[3.3.2 Exclusion Criteria	17](#_Toc172804891)

[3.4 Study Duration	18](#_Toc172804892)

[3.5 Withdrawal from Study Treatment	18](#_Toc172804893)

[3.6 Treatment Plan	18](#_Toc172804894)

[3.6.1 Preoperative Treatment	18](#_Toc172804895)

[3.6.2 Surgical Treatment	18](#_Toc172804896)

[3.6.3 Postoperative Adjuvant Treatment	19](#_Toc172804897)

[3.6.4 Follow-Up	19](#_Toc172804898)

[3.7 Drug Monitoring	19](#_Toc172804899)

[3.7.1 Monitoring	19](#_Toc172804900)

[3.8 Drug Dose Adjustment	19](#_Toc172804901)

[3.8.1 Tislelizumab Dose Adjustment	19](#_Toc172804902)

[3.8.2 Chemotherapy Dose Adjustment	20](#_Toc172804903)

[3.8.3 Criteria for Terminating the Chemotherapy Regimen	21](#_Toc172804904)

[3.9 Concomitant Therapy and Smoking	21](#_Toc172804905)

[3.9.1 Prohibited Medications	21](#_Toc172804906)

[3.9.2 Permitted Concomitant Medications/Therapies	22](#_Toc172804907)

[3.10 Treatment Compliance	22](#_Toc172804908)

[3.11 Study Endpoints	23](#_Toc172804909)

[3.11.1 Safety and Feasibility Endpoints	23](#_Toc172804910)

[3.11.2 Efficacy Endpoints	23](#_Toc172804911)

[3.11.3 Exploratory Endpoints	23](#_Toc172804912)

[3.11.4 Patient Self-Assessment Metrics	23](#_Toc172804913)

[4. Study Assessments and Procedures	24](#_Toc172804914)

[4.1 Screening	24](#_Toc172804915)

[4.1.1 Informed Consent and Screening	24](#_Toc172804916)

[4.1.2 Patient Identification	24](#_Toc172804917)

[4.1.3 Demographic Data and Medical History	24](#_Toc172804918)

[4.1.4 Women of Childbearing Potential and Contraception	24](#_Toc172804919)

[4.2 Enrollment Eligibility Confirmation	25](#_Toc172804920)

[4.3 Safety Assessments	25](#_Toc172804921)

[4.3.1 Vital Signs	25](#_Toc172804922)

[4.3.2 Physical Examination	25](#_Toc172804923)

[4.3.3 ECOG Performance Status (PS) Assessment	25](#_Toc172804924)

[4.3.4 Laboratory Safety Tests	25](#_Toc172804925)

[4.3.5 Pulmonary Function Tests	26](#_Toc172804926)

[4.3.6 Electrocardiogram (ECG)	26](#_Toc172804927)

[4.3.7 Imaging Assessments and Evaluations	26](#_Toc172804928)

[4.4 Biomarkers	28](#_Toc172804929)

[4.5 Follow-Up	28](#_Toc172804930)

[4.6 Visit Windows	28](#_Toc172804931)

[4.7 Unscheduled Visits	29](#_Toc172804932)

[5. Safety Monitoring and Reporting	29](#_Toc172804933)

[5.1 Risks Related to Study Drugs	29](#_Toc172804934)

[5.1.1 Risks Related to Tislelizumab	29](#_Toc172804935)

[5.1.2 Risks Related to Chemotherapy	29](#_Toc172804936)

[5.2 Comprehensive Safety Management Plan	29](#_Toc172804937)

[5.2.1 Inclusion and Exclusion Criteria	29](#_Toc172804938)

[5.2.2 Safety Monitoring Plan	30](#_Toc172804939)

[5.3 Adverse Events	30](#_Toc172804940)

[5.3.1 Definitions and Reporting	30](#_Toc172804941)

[5.3.3 Causality Assessment	31](#_Toc172804942)

[5.3.4 Follow-up of Adverse Events	32](#_Toc172804943)

[5.3.5 Clinically Significant Abnormal Laboratory Test Results	32](#_Toc172804944)

[5.4 Definition of Serious Adverse Events	32](#_Toc172804945)

[5.5 Suspected Unexpected Serious Adverse Reactions (SUSAR)	33](#_Toc172804946)

[5.6 Time, Frequency, and Method of Recording Adverse Events (AE) and Serious Adverse Events (SAE)	33](#_Toc172804947)

[5.6.1 Adverse Event Reporting Period	33](#_Toc172804948)

[5.6.2 Reporting Serious Adverse Events	33](#_Toc172804949)

[5.6.3 Active Inquiry of Adverse Events	34](#_Toc172804950)

[5.6.4 Disease Progression	34](#_Toc172804951)

[5.6.5 Death	34](#_Toc172804952)

[5.6.6 Pregnancy	34](#_Toc172804953)

[5.6.7 Evaluation and Recording of Immune-Related Adverse Events	34](#_Toc172804954)

[5.7 Management of Special Attention AEs	35](#_Toc172804955)

[5.7.1 Infusion-Related Reactions	35](#_Toc172804956)

[5.7.2 Severe Hypersensitivity Reactions and Flu-Like Symptoms	36](#_Toc172804957)

[5.7.3 Immune-Related Adverse Events	36](#_Toc172804958)

[5.7.4 Renal Dysfunction	38](#_Toc172804959)

[6. Statistical Methods and Sample Size Determination	38](#_Toc172804960)

[6.1 Statistical Analysis	38](#_Toc172804961)

[6.1.1 Analysis Sets	38](#_Toc172804962)

[6.1.2 Demographic and Baseline Characteristics	38](#_Toc172804963)

[6.2 Safety and Feasibility Analysis	38](#_Toc172804964)

[6.3 Efficacy Analysis	39](#_Toc172804965)

[6.4 Exploratory Efficacy Analysis	40](#_Toc172804966)

[6.5 Sample Size Considerations	40](#_Toc172804967)

[7. Ethical and Legal Considerations	40](#_Toc172804968)

[7.1 Ethical Standards	40](#_Toc172804969)

[7.2 Independent Ethics Committee (IEC) or Institutional Review Board (IRB)	40](#_Toc172804970)

[7.3 Protocol Amendments	40](#_Toc172804971)

[7.4 Subject Information and Informed Consent	40](#_Toc172804972)

[7.5 Patient and Data Confidentiality	41](#_Toc172804973)

[8. Study Monitoring	41](#_Toc172804974)

[9. Data Handling and Record Keeping	41](#_Toc172804975)

[9.1 Case Report Forms	41](#_Toc172804976)

[9.2 Data Collection	42](#_Toc172804977)

[9.3 Archiving	42](#_Toc172804978)

[9.4 Investigator's File/Document Retention	42](#_Toc172804979)

[9.5 Audits and Inspections	42](#_Toc172804980)

[10. References	43](#_Toc172804981)

[Appendix 1. Assessment Schedule	47](#_Toc172804982)

[Appendix 2. ECOG Performance Status	50](#_Toc172804983)

[Appendix 3. RECIST Guideline for Solid Tumors (Version 1.1)	51](#_Toc172804984)

[Appendix 4. New York Heart Association (NYHA) Functional Classification	59](#_Toc172804985)

[Appendix 5. Assessment and Management of Immune-Related Adverse Events	60](#_Toc172804986)

1. Background and Rationale

1.1 Lung Cancer

Primary bronchogenic lung cancer is one of the most common malignant tumors. According to the latest data from 2019, lung cancer has the highest incidence rate (12.9%) and mortality rate (23.5%) among malignant tumors[1]. In China, 2015 data showed that lung cancer also ranked first in incidence (19.6%) and mortality (26.0%) among all malignant tumors, a significant increase compared to the 2007 incidence (13.0%) and mortality (18.0%) rates. The incidence and mortality rates are highest among men, while for women, lung cancer incidence ranks second (below breast cancer) but first in mortality[2-4]. Approximately 25% of global lung cancer patients are in the Asia-Pacific region, making lung cancer a formidable killer both globally and in Asia.

1.2 Small Cell Lung Cancer

Small cell lung cancer (SCLC) is a heterogenous neuroendocrine tumor originating from Kulchitsky cells of the bronchial mucosal epithelium and is the most aggressive subtype of lung cancer, accounting for about 15%-20% of all lung cancers[5, 6]. SCLC has two staging systems: the 8th edition TNM staging and the Veterans Affairs Lung Study Group (VALSG) staging system. The latter is the most commonly used in clinical practice and divides SCLC into limited-disease (LD-SCLC) and extensive-disease (ED-SCLC). LD-SCLC is confined to one hemithorax and can be safely encompassed within a tolerable radiation field, accounting for 30%-40% of SCLC cases. ED-SCLC extends beyond one hemithorax, including distant metastases and malignant pleural or pericardial effusions[7]. Smoking accounts for 98% of SCLC cases, characterized by high malignancy, aggressive nature, rapid progression, and early distant metastasis[8, 9]. Although SCLC is highly sensitive to chemoradiotherapy, it has a short remission period and a high relapse rate. Most SCLC patients are elderly with a smoking history or heavy smokers, often with multiple chronic underlying diseases, leading to generally poor treatment outcomes, with a median overall survival (mOS) of less than 1 year and a two-year survival rate of less than 5%.

1.3 Current Treatment of Limited-Disease Small Cell Lung Cancer

Chemotherapy remains the cornerstone of SCLC treatment throughout the course of the disease. The application range of radiotherapy has broadened and become more precise and individualized. Targeted therapy is still under research without mature treatment plans, and surgery has become an important part of comprehensive SCLC treatment. Immunotherapy combined with chemotherapy has emerged as a new first-line treatment option and is starting to play a crucial role in SCLC treatment.

Compared to non-small cell lung cancer (NSCLC), SCLC has a higher metastatic potential and worse survival outcomes. Since the 1980s, multiple clinical trials have shown that platinum combined with etoposide achieves higher complete remission rates and better prognoses in SCLC, establishing its foundation in SCLC treatment regimens[10, 11]. A phase III clinical trial published in 2002 also demonstrated that LD-SCLC patients receiving the etoposide+cisplatin (EP) regimen had better prognoses[12]. SCLC is also very sensitive to radiotherapy, especially for LD-SCLC. Current guidelines recommend the EP regimen combined with thoracic radiotherapy as the first-line treatment for LD-SCLC[6].

A meta-analysis[13] showed that concurrent chemoradiotherapy reduced local recurrence rates by 25%-30% and improved the two-year survival rate by 5%-7% compared to chemotherapy alone, highlighting the significant role of radiotherapy in LD-SCLC treatment. Although the timing of radiotherapy has been controversial, three clinical studies comparing concurrent and sequential chemoradiotherapy have confirmed that concurrent chemoradiotherapy is significantly more effective than sequential chemoradiotherapy[14-16].

In recent years, targeted drugs have increasingly been applied in the treatment of malignant tumors, with many clinical trials exploring the role of targeted therapy in SCLC patients, such as anti-angiogenesis drugs, PARP inhibitors, antibody-drug conjugates, and multi-target inhibitors. However, most trial results have not met expectations[17-20]. Given the crucial role of chemoradiotherapy in treating SCLC patients, surgery has been controversial. The only two randomized studies did not show benefits from surgery for patients[21, 22]. However, these studies were conducted early with design deficiencies, likely affecting the results. Post-2000, numerous retrospective studies have indicated that early or LD-SCLC patients can benefit from surgery[23-26]. Other studies also show that surgery combined with chemotherapy can significantly improve the prognosis of early or LD-SCLC patients[27-29], suggesting that surgery combined with chemotherapy could benefit LD-SCLC. However, there is still debate on whether surgery or neoadjuvant chemotherapy followed by surgery should be the first choice for LD-SCLC patients (Table 1).

Research by Fujimori et al. found that the three-year survival rates for stage I-II and stage III SCLC patients were 73.3% and 42.9%, respectively, after neoadjuvant chemotherapy and surgery[30]. A prospective single-center study by Veronesi et al. indicated that SCLC patients could benefit from surgery after neoadjuvant chemotherapy if they achieved complete remission and were pathologically staged as stage I[31].

| Table 1. Clinical Studies Related to Small Cell Lung Cancer (SCLC) | | | |  |  |
| --- | --- | --- | --- | --- | --- |
| **Study** | **Stage** | **Treatment** | **Sample Size** | **5-years OS** | ***P*** |
| Schreiber^23^ | LD-SCLC | Surgery vs. Non-surgery | 863 vs 13316 | 42% vs 13% | <0.001 |
| Luchtenborg^24^ | LD-SCLC | Surgery vs. Non-surgery | 465 vs 45383 | 31% vs 3% | <0.001 |
| James^25^ | I | Surgery + RT vs. Non-surgery | 38 vs 205 | 57.1% vs 50.3% | 0.71 |
| Weksler^26^ | I - II | Surgery vs. Non-surgery | 893 vs 2666 | 26.9% vs 7% | <0.001 |
| Yang^27^ | T1-2N0M0 | Surgery + chemotherapy (Raditherapy) vs. Non-surgery | 544 vs 388 | 52.7% vs 40.4% | <0.01 |
| Tsuchiya^28^ | I – IIIA | Surgery + (Carboplatin + Paclitaxel) | 35 vs 26 | 70% vs 38.5% | 0.10 |
| Yang^29^ | T1-2N0M0 | Surgery + Chemotherapy vs. Chemoradiotherapy | 501 vs 501 | 47.6% vs 29.8% | <0.01 |

In recent years, immunotherapy has become a major focus of tumor research due to its unique mechanism of action, raising high hopes among tumor researchers. SCLC is highly heterogeneous, genomically unstable, and has a high tumor mutation burden, theoretically making it more sensitive to immunotherapy. Thus, many exploratory clinical trials have been conducted in the SCLC field. Current trials mainly target ED-SCLC patients and primarily involve combination therapies, including immunotherapy with chemotherapy, immunotherapy with targeted drugs, and combinations of different immunotherapies. The CTLA-4 inhibitor ipilimumab was the first immunotherapy drug studied in SCLC. A phase III clinical trial found that ipilimumab combined with standard chemotherapy did not improve OS and PFS in ED-SCLC patients[32]. Similar to CTLA-4, the PD-1 receptor and its ligand (PD-L1) are another promising immune checkpoint that could better restore the body's immune function against tumors. A phase II clinical trial showed that maintenance therapy with pembrolizumab after chemotherapy did not improve mPFS in ED-SCLC patients[33]. Another phase III clinical trial[34] explored the use of nivolumab alone or in combination with ipilimumab for second-line and above treatment of sensitive relapsed SCLC, showing that the combination group had higher ORR (23% vs 11%) and OS (7.8 months vs 4.1 months) compared to the monotherapy group. Therefore, more clinical studies are needed to further explore the role of immunotherapy drugs in SCLC.

1.4 Tumor Immunotherapy

Host immunity is fundamental to inhibiting human cancer, and tumor cells escaping host immunity is a crucial pathway in the development of human cancer. The three phases of tumor immune editing are elimination (host immune response to tumor neoantigens and destruction of tumor cells), equilibrium (residual tumor cells that escape immune destruction continue to grow and metastasize under host immune suppression), and escape (tumor cells overcome immune control and develop into clinically evident cancer). Clinically visible tumors indicate that the host immune system cannot recognize and eliminate early cancers, likely due to immune tolerance induced among tumor-specific T cells and the expression of immune-suppressive ligands at checkpoints[35]. These ligands bind to receptors on T cells, signaling to downregulate effector functions such as cytokine production and cytotoxic activity. Therefore, strategies that enhance host antitumor immunity and generate durable immune responses have the potential for long-term tumor control or even cure.

1.5 Immunotherapy Target: PD-1

Programmed cell death protein 1 (PD-1 or CD279) is mainly expressed on activated T cells, B cells, and myeloid cells. It is a 55 kDa type I transmembrane protein and a member of the T cell costimulatory receptor CD28 family, which also includes CD28, CTLA-4, ICOS, and BTLA[35]. Two specific PD-1 ligands have been identified: PD-L1 (also known as B7-H1 or CD274) and PD-L2 (also known as B7-DC or CD273), primarily expressed on antigen-presenting cells. PD-L1 and PD-L2 have been confirmed to downregulate T cell activation upon binding to PD-1 in both mouse and human systems[36, 37]. Studies have reported PD-L1 expression in SCLC[38], which might lead to immune evasion and tumor progression upon PD-1 and PD-L1 binding. This hypothesis is supported by independent research showing that PD-L1 expressed by tumor cells increases apoptosis of activated tumor-specific T cells in vitro and protects tumor cells from effector T cell-induced apoptosis[39]. Preclinical data show that PD-1 blockade is effective in both PD-L1 positive and negative tumors, suggesting its role at the level of immune initiation and the tumor microenvironment[40]. This might be related to host mechanisms (i.e., PD-L1 expression on antigen-presenting cells) that limit antitumor responses. Thus, both PD-L1 positive and negative tumors may benefit from immunotherapy.

1.6 Anti-PD-1 Drug: Tislelizumab (BGB-A317)

Tislelizumab (BGB-A317) is a humanized IgG4 subtype monoclonal antibody targeting PD-1, currently under clinical development for the treatment of various human malignancies. It is an IgG4 subtype antibody engineered to mitigate interaction with Fcγ receptors (e.g., FcγRI and FcγRIIIA). It demonstrates low affinity for complement component 1q (C1q). In vitro studies indicate that tislelizumab exhibits minimal or no effect on antibody-dependent cellular cytotoxicity (ADCC), antibody-dependent cellular phagocytosis (ADCP), or complement-dependent cytotoxicity (CDC) [41, 42]. Tislelizumab exerts its pharmacological action by binding with high specificity and affinity (dissociation constant [KD]=0.15 nM) to the extracellular domain of human PD-1, competitively blocking the interaction of PD-1 with its ligands, PD-L1 and PD-L2, thereby inhibiting PD-1-mediated negative signal transduction in T cells. In vitro cellular assays have shown that tislelizumab persistently and dose-dependently enhances the functional activity of human T cells and pre-activated peripheral mononuclear cells. Additionally, tislelizumab has demonstrated antitumor activity in several xenograft models where human peripheral blood mononuclear cells and either human cancer cells (A431 [epithelial carcinoma]) or tumor fragments (BCCO-028 [colon carcinoma]) were co-injected into immunocompromised mice.

1.7 Current Status of PD-1 Pathway-Targeted Therapy for SCLC Patients

With the increasing variety of investigational immunotherapy drugs, clinical research has grown annually, diversifying treatment options and offering new hope for SCLC treatment. Targeting the PD-1 pathway alone or in combination with anti-cytotoxic T-lymphocyte-associated protein 4 (CTLA-4) drugs has shown encouraging efficacy signals in previously treated ES-SCLC patients. The phase I/II CheckMate 032 study investigated the efficacy of the anti-PD-1 human IgG4 antibody nivolumab (3 mg/kg) monotherapy, or sequential nivolumab maintenance after combining with the anti-CTLA-4 monoclonal antibody ipilimumab, in patients who progressed after at least one line of platinum-based systemic chemotherapy. The objective response rate (ORR) was 12% (95% CI: 6.5, 19.5), with a median response duration of 13 months (3.0, 42.1) [34]. Based on these data, the US FDA granted accelerated approval for nivolumab in August 2018 for the treatment of metastatic SCLC patients who have progressed after platinum-based chemotherapy and at least one other line of therapy. The KEYNOTE-028 study investigated another anti-PD-1 monoclonal antibody, pembrolizumab. KEYNOTE-028 was a non-randomized, multi-cohort phase Ib trial conducted in patients with PD-L1-positive advanced solid tumors. Results showed that this PD-1 antibody exhibited similar promising efficacy in previously treated PD-L1-positive (tumor cell staining proportion ≥1%) ES-SCLC patients, with an ORR of 33% (8/24 patients) and a median response duration of 19.4 months, with a 1-year survival rate of 37.7% [43].

Several ongoing clinical trials are exploring the role of PD-1 pathway-targeted antibodies in SCLC treatment, as listed in Table 2. The results of the multicenter, randomized, double-blind phase III CASPIAN trial demonstrated that the PD-L1 antibody durvalumab, in combination with platinum and etoposide, significantly improved overall survival (OS) in ED-SCLC [44]. Similarly, the IMpower133 trial showed that the PD-L1 antibody atezolizumab, combined with carboplatin and etoposide, significantly extended median OS and median progression-free survival (PFS) in SCLC [45]. However, to date, there is no further research confirming the efficacy and safety of neoadjuvant anti-PD-1 therapy in LD-SCLC, nor evaluations of long-term outcomes such as patient survival and recurrence rates. Additionally, administering neoadjuvant anti-PD-1 immunotherapy in an unaffected tumor immune microenvironment has the potential to establish a long-term tumor immune response, reduce tumor size, improve surgical complete resection rates, and eliminate micrometastases. Considering that neoadjuvant therapy delays surgery, posing some risk of progression, more trials are needed to evaluate these aspects.

| Table 2. Studies on PD-1 Pathway Targeted Immunotherapy for SCLC | |  |
| --- | --- | --- |
| Study | Treatment Group | Stage |
| REACTION | Platinum/Etoposide +/- Pembrolizumab | II |
| KEYNOTE 604 | Platinum/Etoposide +/- Pembrolizumab | III |
| IMpower133 | Carboplatin/Etoposide +/- Atezolizumab | III |
| Caspian | Platinum/Etoposide + Durvalumab +/- Tremelimumab vs. Chemotherapy Alone | III |
| MCC-18914 | Platinum/Etoposide, Sequential Thoracic Radiotherapy +/- Nivolumab + Ipilimumab | I/II |
| NCT02402920 | Platinum/Etoposide, Sequential Thoracic Radiotherapy +/- Pembrolizumab | I |

1.8 Research Rationale

SCLC accounts for 15%-20% of all lung malignancies and is the most aggressive subtype of lung cancer, with an overall 5-year survival rate being low. Nearly two-thirds of SCLC cases are diagnosed at an extensive stage, losing the opportunity for surgery or curative treatment, with chemotherapy remaining the standard therapy [10, 11]. Since the 1980s, the combination of etoposide with platinum, along with thoracic radiotherapy, has been the first-line gold standard treatment for SCLC [6]. Although SCLC patients initially respond well to chemoradiotherapy, most will relapse or metastasize within two years and die [46]. Recent studies have shown that immunotherapy significantly improves the efficacy of SCLC treatment to some extent [44, 45]. Tumor cell immune evasion is a critical pathway in the development of human cancers, with research confirming that PD-1 ligands (PD-L1 and PD-L2) downregulate T cell activation upon binding to PD-1, facilitating tumor immune evasion. Thus, immunotherapy has emerged as a new approach to cancer treatment. Anti-PD-1 pathway-targeted immunotherapy for advanced SCLC patients shows significantly better outcomes than traditional chemotherapy. Administering neoadjuvant anti-PD-1 immunotherapy in an unaffected tumor immune microenvironment has the potential to establish a long-term tumor immune response. In NSCLC, neoadjuvant anti-PD-1 immunotherapy has been shown to significantly improve pathological response rates, though long-term efficacy remains uncertain. However, to date, no studies have confirmed the safety and efficacy of neoadjuvant anti-PD-1 therapy in LD-SCLC. We urgently need a well-designed, clearly staged prospective study to clarify the role and impact of tislelizumab in neoadjuvant immunotherapy for resectable LD-SCLC patients.

2. Research Objectives

To evaluate the safety and feasibility of neoadjuvant anti-PD-1 drug tislelizumab combined with etoposide and platinum in treating resectable LD-SCLC, the primary pathological response rate, and long-term tumor efficacy indicators.

The primary endpoints of this study are to observe the safety and feasibility of neoadjuvant anti-PD-1 therapy combined with etoposide and platinum in treating LD-SCLC. Secondary endpoints include major pathological response rate, 5-year overall survival (5-y OS), progression-free survival (PFS), overall survival (OS), quality of life (QoL), and R0 resection rate.

Exploratory endpoints: To explore potential predictive biomarkers in blood and tumor tissue, and to analyze lymphocyte infiltration in the tumor.

3. Study Design

3.1 Study Design

This is an open-label, non-randomized, single-center clinical study aimed at evaluating the safety and feasibility of preoperative neoadjuvant anti-PD-1 therapy combined with etoposide and platinum-based chemotherapy in resectable LD-SCLC. The study flow chart is as follows:

3.2 Sample Size

Approximately 15 patients are expected to participate in the study.

3.3 Participant Selection

3.3.1 Inclusion Criteria

- Signed informed consent;

- Age ≥ 18 years;

- Histologically or cytologically confirmed SCLC (confirmed by biopsy, bronchoscopy, etc.), initial treatment patients with imaging-confirmed LD-SCLC;

- ECOG performance status score of 0-1;

- Expected survival of at least 12 weeks;

- Measurable lesions;

- Good function of other major organs (liver, kidney, hematologic system, etc.):

- Hemoglobin ≥ 9.0 g/dL (maintained by transfusion or above this level);

- Red blood cell count ≥ 2.0×10^12/L;

- Absolute neutrophil count (ANC) ≥ 1.0×10^9/L;

- Platelet count ≥ 100×10^9/L;

- Serum total bilirubin ≤ 1.5 times the upper limit of normal (ULN);

- ALT, AST, and alkaline phosphatase ≤ 2.5×ULN;

- INR ≤ 1.5 and APTT ≤ 1.5 times the ULN in patients not on anticoagulation therapy. Patients on full or gastrointestinal anticoagulant therapy may enter if the dose has been stable for at least 2 weeks and coagulation test results are within local treatment limits.

- No systemic metastasis;

- Expected complete resection;

- Adequate cardiopulmonary function to tolerate surgery;

- Women of childbearing potential must have a negative pregnancy test within 7 days before starting treatment and use reliable contraception (e.g., IUD, oral contraceptives, condoms) from the start to 30 days after the trial ends;

- Male participants of childbearing potential should use condoms during the trial and for 30 days after its completion.

3.3.2 Exclusion Criteria

Patients meeting any of the following criteria will be excluded:

- Prior systemic anticancer therapy for SCLC, including surgery, local radiotherapy, cytotoxic drugs, targeted therapy, and experimental therapy;

- History of other malignancies within the last five years, except SCLC;

- Unstable systemic diseases such as uncontrolled hypertension or severe arrhythmia;

- Active, known, or suspected autoimmune disease or requiring systemic treatment autoimmune paraneoplastic syndrome;

- Allergy to the study drug;

- History or current interstitial lung disease;

- Concurrent HIV infection or active viral hepatitis;

- Pregnant or breastfeeding women;

- Malabsorption conditions;

- Neurological or psychiatric disorders that prevent cooperation;

- Other conditions deemed unsuitable for enrollment by the investigator, such as patients requiring pneumonectomy.

3.4 Study Duration

With a monthly enrollment of 3 patients, the enrollment period is approximately 5 months; follow-up is 60 months, making the total study duration about 65 months.

3.5 Withdrawal from Study Treatment

Patients will be withdrawn from study treatment under the following conditions:

- Patient or legal representative requests withdrawal;

- Investigator determines that continued participation is harmful to the patient's health;

- Severe allergic reactions to chemotherapy drugs and tislelizumab;

- Other serious adverse reactions deemed by the principal investigator or designated researchers to require treatment interruption;

- Poor compliance;

- Positive β-HCG test indicating pregnancy (to be reported using the clinical trial pregnancy report form);

- Emergence of other diseases that significantly affect clinical evaluation and require cessation of the study protocol;

- Emergence of other malignancies requiring treatment;

- Loss of follow-up;

- Use of prohibited drugs or substances that may cause toxic reactions or skew study results;

- Patient death.

All reasons for withdrawal should be documented in the case report form and patient medical records. Patients withdrawn due to adverse events or laboratory abnormalities should be followed until the event resolves or stabilizes, with outcomes recorded. Deaths occurring during the trial or within 28 days of trial completion should be reported to the investigator within 24 hours using the serious adverse event (SAE) report form and notified to the ethics department.

3.6 Treatment Plan

3.6.1 Preoperative Treatment

All enrolled patients will receive four treatment cycles, each lasting 3 weeks, with the following regimen:

1. Tislelizumab: 200 mg/administration, intravenous infusion (30-60 minutes) on day 1 of each cycle;

2. Platinum-based doublet chemotherapy (etoposide and platinum regimen): cisplatin 75 mg/m² or carboplatin (AUC5) + etoposide 100 mg/m², intravenous infusion. Cisplatin/carboplatin is administered on day 1, etoposide on days 1, 2, and 3 of each cycle.

3.6.2 Surgical Treatment

The maximum interval from the first dose of neoadjuvant therapy to surgery is 30 weeks. Patients receiving fewer than 4 cycles of neoadjuvant therapy may continue in the study and should undergo surgery within 4-8 weeks after the last study treatment. Surgery must achieve non-palliative complete resection with negative margins.

3.6.3 Postoperative Adjuvant Treatment

Postoperative adjuvant chemoimmunotherapy is given up to 4 cycles. Adjuvant radiotherapy may be added based on physician recommendation and patient preference, and treatment may be received at the study site or external facilities.

3.6.4 Follow-Up

Follow-up includes symptom monitoring, chest CT scans, whole-body ECT if bone pain is present, and brain MRI for symptoms like headache, vomiting, or gait disturbances. Postoperative follow-up occurs at 3 months, then every 6 months for 2 years if no recurrence or metastasis is detected. After 2 years without recurrence, follow-up transitions to telephone survival follow-up every 3 months ±7 days. For patients with recurrence or metastasis, follow-up switches to post-progression survival follow-up every 3 months ±14 days.

3.7 Drug Monitoring

3.7.1 Monitoring

All patients receiving tislelizumab or etoposide and platinum-based chemotherapy must undergo complete blood cell count tests, including platelet counts. Monitor patients’ nadir and recovery levels with tests before each administration and on days 8 and 15 of each cycle. Regular biochemical tests to evaluate liver and kidney functions should be done before each administration. The next treatment cycle can only commence if neutrophil count ≥ 1.5×10^9/L, platelet count ≥ 100×10^9/L, creatinine clearance rate ≥ 45 mL/min, total bilirubin ≤ 1.5×ULN, alkaline phosphatase (AP), AST, and ALT ≤ 3×ULN.

3.8 Drug Dose Adjustment

3.8.1 Tislelizumab Dose Adjustment

Dose reduction is not recommended. Discontinue administration in the following situations:

- Grade 2 pneumonitis;

- AST or ALT > 3 and ≤ 5×ULN or total bilirubin > 1.5 and ≤ 3×ULN;

- Grade 2 or 3 diarrhea or colitis;

- Symptomatic hypophysitis, adrenal insufficiency, hypothyroidism, hyperthyroidism, or grade 3 or 4 hyperglycemia;

- Grade 2 ocular inflammation toxicity;

- Grade 2 or 3 pancreatitis or grade 3 or 4 amylase or lipase level increases (> 2.0×ULN);

- Grade 3 or 4 infections;

- Grade 2 infusion-related reactions;

- Grade 3 rash.

Tislelizumab may be resumed after adverse reactions resolve to grade 0-1. Immediately discontinue for:

- Grade 3 or 4 pneumonitis;

- AST or ALT > 5×ULN or total bilirubin > 3×ULN;

- Grade 4 diarrhea or colitis;

- Grade 4 hypophysitis;

- Myasthenia syndrome/Graves’ disease, Guillain-Barré syndrome, or encephalitis (any grade);

- Grade 3 or 4 ocular inflammation toxicity;

- Grade 4 or recurrent pancreatitis (any grade);

- Grade 3 or 4 infusion-related reactions;

- Grade 4 rash.

3.8.2 Chemotherapy Dose Adjustment

Chemotherapy dose adjustments should be based on the investigator's clinical judgment, adhering to the prescribing information and clinical practice guidelines. Doses are calculated using the baseline weight. If the patient’s weight changes by ≥10% from the baseline (or new reference weight), dose adjustments are necessary. Toxicity related to the study drug must regress to baseline, grade 0, or grade 1 before administering the next dose, with the exception of alopecia or grade 2 fatigue. Each chemotherapy drug allows for a maximum of two dose reductions. Once a dose is reduced, all subsequent doses should maintain that reduced dose or be further reduced if necessary. There is no dose escalation in this study. If a further dose reduction is needed, chemotherapy must be discontinued.

In the case of chemotherapy-related toxicity, chemotherapy can proceed if the adverse event (AE) resolves within 10 days. Chemotherapy or tislelizumab administration will resynchronize in subsequent cycles based on the chemotherapy administration dates. If the AE does not resolve within 10 days, chemotherapy is withheld. If the AE resolves within 21 days, chemotherapy or tislelizumab will resume on Day 1 of the next treatment cycle according to the original schedule.

**Selected Precautions:**

- Neutropenia: Prompt evaluation and aggressive treatment according to clinical practice guidelines should be conducted for fever or other signs of infection.

- Nephrotoxicity:

- Nephrotoxicity is a common adverse event with cisplatin. Oral hydration is recommended. Avoid nephrotoxic drugs such as aminoglycoside antibiotics.

- Patients with a creatinine clearance (CrCl) < 60 mL/min should not receive cisplatin.

- Ototoxicity and sensory neuropathy should be evaluated before each cycle. Cisplatin is contraindicated in patients with pre-existing hearing impairment.

For toxicities not listed above, dose adjustments are allowed according to clinical standards.

3.8.3 Criteria for Terminating the Chemotherapy Regimen

Chemotherapy drugs should be discontinued if any of the following conditions occur, in addition to the specific situations mentioned above:

- Any grade 4 peripheral neuropathy necessitates discontinuation of cisplatin.

- Persistent grade 3 sensory abnormalities require discontinuation of cisplatin.

- Grade 3 or 4 drug-related thrombocytopenia with clinically significant bleeding.

- Grade 3 or 4 total bilirubin elevation and grade 4 ALT or AST elevation necessitate discontinuation of etoposide.

- Discontinuation of treatment is required for drug-related liver function test abnormalities meeting any of the following criteria:

- AST or ALT > 5-10× ULN for more than 2 weeks.

- ALT or AST > 10× ULN.

- Total bilirubin > 5× ULN.

- Concurrent AST or ALT > 3× ULN and total bilirubin > 2× ULN.

- Discontinuation of cisplatin is required if any cisplatin-related creatinine clearance falls below <30 mL/min (calculated using the Cockcroft-Gault formula).

- Discontinuation of etoposide is required if creatinine clearance falls below <15 mL/min.

- Discontinuation of treatment is required if the same drug-related AE recurs after two dose reductions.

- Any grade 3 or 4 drug-related hypersensitivity or infusion reaction necessitates discontinuation of the implicated drug. Drugs not considered related to the hypersensitivity or infusion reaction may continue to be administered.

- Any grade 4 AE related to the study drug that is deemed unsuitable for dose reduction by the investigator requires discontinuation of the implicated drug. Drugs not considered related to the event may continue to be administered.

- Discontinuation of the drug component is required if the toxicity does not resolve within 21 days.

For toxicities not listed above, the investigator should make a medical judgment based on the patient's health status and clinical standards to determine whether to terminate the chemotherapy regimen.

3.9 Concomitant Therapy and Smoking

All concomitant medications and treatments (including start/stop dates and indications) must be recorded in the patient’s original data and the corresponding part of the case report form (CRF). All patients are advised to quit smoking during treatment.

3.9.1 Prohibited Medications

- Immunosuppressants (except those used to treat drug-related AEs).

- Systemic corticosteroids >10 mg/day (prednisone or equivalent), except for the treatment or control of drug-related AEs (according to the study protocol), as premedication for chemotherapy, or for short-term prophylactic treatment.

- Live vaccines administered within 28 days before enrollment and within 60 days after the last dose of tislelizumab.

- Herbal medicines with immune-stimulating properties (e.g., mistletoe extracts) or those known to interfere with liver or other major organ functions (e.g., St. John’s wort). Patients must inform the investigator of all herbal medicines used during the study. Herbal treatment is not recommended as their pharmacokinetics, safety profiles, and potential drug-drug interactions are usually unknown. However, patients may use herbal medicines with the investigator's approval if there are no known interactions with the study treatment. Herbal treatments for cancer are prohibited as mentioned above.

- RANK-L inhibitors (e.g., denosumab): Patients who received denosumab treatment before enrollment must be willing and suitable to switch to bisphosphonate treatment.

3.9.2 Permitted Concomitant Medications/Therapies

Most concomitant medications and therapies deemed necessary by the investigator and consistent with clinical treatment standards for supportive care (e.g., antiemetics, antidiarrheals) and in the best interest of the patient are permitted.

Systemic corticosteroids required to control irAEs must be tapered to non-immunosuppressive doses (prednisone or equivalent ≤10 mg/day) before the next dose of the study drug. Short-term steroid use for prophylactic treatment (e.g., for patients allergic to contrast agents used in imaging) is permitted.

The use of hematopoietic growth factors (i.e., G-CSF or GM-CSF) to treat febrile neutropenia is allowed, but these factors cannot be used as primary prophylactic treatment. Growth factors must be discontinued at least 48 hours before the next study drug treatment cycle begins. Any use of growth factor support must be recorded in the patient's records and the CRF.

Patients may continue hormone replacement therapy or supportive therapy initiated before enrollment. If bisphosphonate treatment for bone metastases was started before enrollment and the dose is stable, it may continue during the study. Bisphosphonate use for non-malignant tumor indications is allowed during the study.

3.10 Treatment Compliance

The use, dose, and administration time of tislelizumab and etoposide and platinum-based chemotherapy for each cycle should be recorded in the CRF. The reasons for delayed administration, dose reduction, or missed doses must also be documented in the CRF.

Subject compliance with treatment and protocol includes voluntary adherence to all aspects of the protocol. Subjects who do not adhere to scheduled follow-up visits or medication administration may be terminated at the discretion of the principal investigator.

3.11 Study Endpoints

3.11.1 Safety and Feasibility Endpoints

Safety is defined as the frequency of serious adverse events occurring within 90 days after the last preoperative PD-1 drug administration or within 30 days post-surgery, or before starting new treatment (whichever occurs first), based on the NCI CTCAE version 5.0 criteria. Safety analysis will mainly use descriptive methods and percentages, listing the AEs occurring in this trial.

Feasibility will be assessed by the successful completion of preoperative treatment and surgery without any prolonged treatment-related delays. Delayed treatment is defined as extending > 37 days from the pre-planned Day 0.

3.11.2 Efficacy Endpoints

- Major Pathological Response (MPR): The proportion of patients achieving a major pathological response, defined as less than 10% of residual viable tumor cells in the resected tumor as determined by HE staining.

- Objective Response Rate (ORR): The proportion of patients with measurable disease at baseline who achieve complete response (CR) or partial response (PR) according to RECIST v1.1. Patients without post-baseline assessments will be considered non-responders.

- 5-Year Overall Survival (5-year OS): Defined as the time from the start of treatment to death from any cause within 5 years. Survival curves will be calculated using the Kaplan-Meier method.

- Disease-Free Survival (DFS): Defined as the time from the start of treatment to the first confirmed recurrence of disease or death from any cause (whichever occurs first).

- Overall Survival (OS): Defined as the time from the start of initial treatment to death from any cause. Patients alive at the time of analysis will have their last contact date used as the cutoff date. Survival curves will be calculated using the Kaplan-Meier method.

- R0 Resection Rate: R0 resection is defined as a negative surgical margin with no residual tumor visible under the microscope.

3.11.3 Exploratory Endpoints

Exploring potential predictive biomarkers in blood and tumor tissue, analyzing lymphocyte infiltration in tumors, etc.

3.11.4 Patient Self-Assessment Metrics

The Shanghai Pulmonary Hospital Patient Survey Questionnaire will be completed at baseline (Day 1 of Cycle 1) and at the end of each cycle during the neoadjuvant treatment phase (pre-surgery Day -20, Day -1). The questionnaire should be completed independently by the patient at the beginning of each visit, prior to any interaction with the physician, to ensure that the patient's responses are not influenced by any contact with the doctor.

4. Study Assessments and Procedures

Refer to Appendix 1 for the study assessment schedule. Patient safety and tolerability will be closely monitored throughout the study. All assessments for each patient must be completed and recorded in the patient’s medical records. Treatment administration is contingent upon reviewing clinical assessments and laboratory values (which must be obtained before any dosing) and determining them to be acceptable according to the protocol guidelines.

4.1 Screening

Screening assessments will be conducted within 35±7 days before enrollment. Patients who consent to participate will sign an informed consent form before starting the screening procedures. The screening period begins on the first day of the screening procedures. At baseline, a physical examination, laboratory assessments, and pulmonary function tests will be conducted for all patients (Appendix 1). Screening assessments may be repeated as needed; eligibility will be evaluated based on the most recent screening results.

4.1.1 Informed Consent and Screening

A voluntarily signed written informed consent must be obtained before any study-specific procedures are performed. Informed consent forms for both enrolled and screening-failed patients will be retained at the study center. All screening assessments must be completed and reviewed, and patients must meet all eligibility criteria before enrollment.

4.1.2 Patient Identification

Upon obtaining the informed consent form, researchers will assign a unique patient number to potential study participants.

4.1.3 Demographic Data and Medical History

Demographic data include the year of birth (or age) and gender. Medical history includes any clinically significant disease history, surgical history, or cancer history; fertility status (i.e., fertile or infertile); history of alcohol and tobacco use (i.e., past, current, or never). Cancer history includes evaluations of prior surgeries, radiation therapy, and drug treatments, including the dates of initiation and cessation, best response, and reasons for discontinuation. Imaging results conducted before study enrollment may be collected for investigator review.

4.1.4 Women of Childbearing Potential and Contraception

Women of childbearing potential are defined as those physiologically capable of becoming pregnant.

4.2 Enrollment Eligibility Confirmation

The investigator will evaluate and confirm the eligibility of each patient. All screening results and relevant medical history must be obtained before confirming eligibility. All inclusion criteria must be met, and no exclusion criteria should be present.

4.3 Safety Assessments

4.3.1 Vital Signs

Vital signs include body temperature (˚C), respiratory rate, heart rate, and blood pressure (systolic and diastolic) measured after 10 minutes of rest in a seated position. Height (only at baseline) and weight should also be measured and recorded in the Case Report Form (CRF). Vital signs should be measured during the screening period (−D42 to D0), before the start of each treatment cycle, at the end-of-treatment assessment, and during the safety assessment period. Additionally, vital signs may be measured once during infusion and 30 minutes post-infusion as clinically indicated.

4.3.2 Physical Examination

A comprehensive physical examination, including evaluations of 1) head, eyes, ears, nose, throat, 2) cardiovascular system, 3) skin, 4) musculoskeletal system, 5) respiratory system, 6) gastrointestinal system, and 7) nervous system, will be performed during the screening period (−D42 to D0), before the start of each treatment cycle, at the end-of-treatment assessment, and during the safety assessment period. Any abnormalities discovered during the screening period will be graded according to NCI-CTCAE v5.0 and recorded in the CRF using appropriate disease/condition terminology.

4.3.3 ECOG Performance Status (PS) Assessment

ECOG PS (Appendix 2) will be assessed during the screening period (−D42 to D0), on Day 1 of each treatment cycle, at the end-of-treatment assessment, and during the safety follow-up visit.

4.3.4 Laboratory Safety Tests

Laboratory tests, including serum biochemistry, hematology, coagulation, and urinalysis, will be conducted as specified below. The following hematology and serum biochemistry tests will be performed during the screening period, before the start of each cycle of induction and maintenance therapy, at the end-of-treatment assessment, and during the safety assessment period:

- Hepatitis Markers: HBV tests (HBsAg, HBsAb, HBeAg, HBeAb, HBcAb, HBcAb IgM); HCV antibody test.

- HIV Antibody Test.

- Thyroid Function: FT3, FT4, TSH, and triiodothyronine (T3).

- Complete Blood Count (CBC): Hemoglobin, red blood cell count, white blood cell count, neutrophil count, lymphocyte count, and platelet count.

- Serum Biochemistry: Lactate dehydrogenase (LDH), alkaline phosphatase (ALP), gamma-glutamyl transferase (GGT), total cholesterol (TC), triglycerides (TG), total protein (TP), albumin (ALB), fasting glucose (GLU).

- Liver and Kidney Function and Electrolytes: Alanine aminotransferase (ALT), aspartate aminotransferase (AST), total bilirubin (TBIL), direct bilirubin (DBIL), creatinine (Cr), blood urea nitrogen (BUN), potassium (K+), sodium (Na+), chloride (Cl-).

- Cardiac Enzymes: Creatine kinase (CK), creatine kinase-MB (CK-MB), myoglobin (Mb), troponin I (cTnI).

- Coagulation Tests: PT, APTT, INR, TT, FDP.

- Urinalysis: Urine protein (PRO), urine glucose (GLU), urine occult blood (urine red blood cells, urine white blood cells).

4.3.5 Pulmonary Function Tests

Pulmonary function tests, including spirometry and oxygenation assessment, should be conducted during the screening period (−D42 to D0), pre-surgery, at the end-of-treatment assessment, and during the safety follow-up period. At a minimum, pulse oximetry should be performed at rest and during exercise, or diffusing capacity testing, to determine patient eligibility for study participation. Clinical confirmation of enrollment eligibility is required for patients with significantly impaired pulmonary function, such as resting pulse oximetry <90% without oxygen and further decreases during exercise, forced expiratory volume in one second (FEV1) or diffusing capacity of the lung for carbon monoxide (DLCO) below 60% of predicted values adjusted for age and gender.

4.3.6 Electrocardiogram (ECG)

ECG recordings will be obtained during the screening period, at the end-of-treatment assessment, and during the safety follow-up visit, as clinically indicated. For safety monitoring purposes, the investigator must review, sign, and date all ECG tracings. ECG tracings, whether in paper or electronic format, will be retained at the study center as part of the permanent study records for the patient.

4.3.7 Imaging Assessments and Evaluations

Tumor imaging assessments should be conducted during the screening period. During the study, tumor imaging assessments will be performed once after completing the second cycle, regardless of any treatment delays. During the first 2 years post-surgery, imaging assessments will be conducted every 3 months, and every 6 months thereafter if no recurrence or metastasis is observed. If recurrence or metastasis occurs, patients enter the survival follow-up phase with telephone follow-ups every 3 months ±14 days. Tumor assessments will continue until imaging-based disease progression is confirmed according to RECIST v1.1.

Patients who discontinue study treatment for reasons other than disease progression (e.g., toxicity) will continue tumor assessments as planned until the start of subsequent anticancer therapy, disease progression, withdrawal of consent, loss to follow-up, death, or study termination, whichever occurs first.

Screening and subsequent assessments include contrast-enhanced CT or MRI of the chest, abdomen, pelvis, and brain (complete assessment during screening; subsequent assessments must include chest contrast-enhanced CT, with others as clinically indicated). Imaging of other known or suspected disease sites must be included (e.g., neck, limbs).

All measurable and evaluable lesions should be assessed and recorded during the screening visit and reassessed during each subsequent tumor evaluation. Consistent imaging procedures (e.g., the same CT scanning protocol) should be used throughout the study for evaluating disease sites identified during screening.

- All screening patients must undergo brain imaging (MRI or CT, MRI preferred) at baseline (within 35±7 days before enrollment).

- If tumor assessments use PET/CT scanners, the CT scans must meet diagnostic CT scan standards.

- Bone scans are only performed at screening if clinically indicated. If bone metastases are identified during screening but not detected by CT or MRI, bone scans should be repeated when complete response (CR) or progression of bone metastases is suspected.

- CT scans of the neck or limbs are only performed at screening if clinically indicated. If metastatic disease is evident in these areas during screening, follow-up evaluations must include these areas throughout the study. Investigators may use other methods to assess target and non-target lesions per RECIST v1.1.

Investigators will evaluate treatment efficacy according to RECIST v1.1. Consistent evaluation by the same assessor is preferred to ensure uniformity across follow-up results.

In immune therapies such as PD-1 monoclonal antibodies, mechanisms such as immune cell infiltration and other factors may cause an apparent increase in tumor volume or the appearance of new tumor lesions, which can result in pseudo-progression. Therefore, if researchers suspect that radiographic disease progression may be pseudo-progression, it is feasible to perform a biopsy to examine pathological tissue for confirmation. If pathological examination is not feasible, patients may continue treatment until radiographic reassessment confirms disease progression ≥4 weeks later (but not exceeding 6 to 8 weeks from the initial date of disease progression recording). Additionally, if a patient is determined to have disease progression based on RECIST v1.1 but, in the researcher’s judgment, may still benefit from continuing study treatment, the patient may continue to receive study treatment. For patients suspected of pseudo-progression or those who may still benefit from study treatment based on the researcher’s judgment after disease progression, the following criteria must be met to continue treatment:

•	No clinical symptoms or signs of disease progression (including significant worsening of clinically relevant laboratory values)

•	ECOG performance status stable and ≤1

•	No rapid disease progression or tumor progression in critical anatomical sites requiring other urgent medical intervention (e.g., spinal cord compression)

•	For treatment continued after radiographic disease progression, the researcher must obtain written informed consent and inform the patient that this approach is not the standard cancer treatment.

Patients continuing treatment after disease progression will continue tumor assessments as per the original schedule or as clinically needed until treatment ends. If study medication is continued after the researcher’s initial assessment of disease progression, this must be documented in the CRF.

4.4 Biomarkers

Tumor tissue specimens, in addition to assessing PD-L1 expression, may also be evaluated for exploratory and predictive biomarkers related to the efficacy or clinical benefit of tiragolumab, such as lymphocyte infiltration in tumors.

For patients confirmed to have disease progression during the study, optional biopsies at accessible tumor sites may be performed to explore resistance mechanisms. If feasible, follow-up biopsy samples should ideally be taken from the same tumor lesions as the baseline biopsy.

During screening, preoperative treatment, surgery, adjuvant therapy (if applicable), and upon disease progression (if applicable), selective blood samples (10 mL per time point) should be collected to explore the relationship between blood-based biomarkers and the efficacy, resistance, and prognosis of tiragolumab in combination with chemotherapy.

4.5 Follow-Up

After treatment ends, patients enter the follow-up period, which includes symptom assessment, chest CT scans, and if bone pain occurs, a whole-body ECT should be performed. If headache, vomiting, or unilateral limb weakness or gait instability occur, a cranial MRI should be conducted. Follow-ups are performed every 3 months ±14 days for 2 years. If no recurrence or metastasis occurs within 2 years post-treatment, the patient will enter a telephone survival follow-up phase, with follow-up calls every 3 months ±14 days. Follow-up includes survival status, time of disease recurrence, recording of the date and cause of death if applicable, and details of any additional antitumor treatments received post-progression. If recurrence or metastasis occurs, the patient will enter the post-progression survival follow-up phase, with telephone follow-ups every 3 months ±14 days, including survival status, date and cause of death if applicable, and details of any additional antitumor treatments received.

4.6 Visit Windows

All visits should be conducted within ±3 days of the scheduled date, unless otherwise specified (see Appendix 1). Unless an acceptable time window is specified, all assessments should be performed on the designated visit dates. If a scheduled visit conflicts with holidays, weekends, or other events, the visit should be arranged on the nearest feasible date (visit time windows are detailed in Appendix 1), with subsequent visits following the planned schedule, every 3 weeks from Day 1 of Cycle 1.

4.7 Unscheduled Visits

Unscheduled visits may be conducted at any time upon request by the patient or investigator and may include vital signs/targeted physical examination, ECOG performance status, AE review, review of concomitant medications and supportive treatments, imaging assessments, physical examinations of the liver, spleen, and lymph nodes, disease-related systemic symptoms, and hematological and biochemical laboratory assessments. The date and reason for unscheduled visits must be recorded in the CRF.

If an unscheduled visit is required to assess toxicity or suspected disease progression, diagnostic tests may be conducted at the investigator’s discretion, and the results should be recorded in the unscheduled visit CRF.

5. Safety Monitoring and Reporting

The investigator is responsible for monitoring and recording events that meet the AE or SAE standards and definitions described in this study protocol.

5.1 Risks Related to Study Drugs

5.1.1 Risks Related to Tislelizumab

Tislelizumab was approved for domestic use in December 2019. For specific risk information, please refer to the drug package insert and the Investigator's Brochure.

5.1.2 Risks Related to Chemotherapy

Please refer to the latest drug package inserts for risk information related to specific cisplatin or etoposide-based chemotherapy.

5.2 Comprehensive Safety Management Plan

5.2.1 Inclusion and Exclusion Criteria

Inclusion and exclusion criteria are designed to protect the safety of patients enrolled in this study. The results of nonclinical toxicology studies and clinical data of tislelizumab, as well as nonclinical and clinical data from other PD-L1/PD-1 inhibitors, have been considered. Specifically, patients at risk for active autoimmune diseases during the study or with a history of recurrent autoimmune diseases, as well as patients who received vaccinations within 4 weeks prior to enrollment, are excluded from this study. Patients with contraindications to the comparator drugs are also excluded.

5.2.2 Safety Monitoring Plan

The safety of this study will be assessed by monitoring all AEs, defined and graded according to NCI-CTCAE v5.0. Patient safety (including laboratory values) will be assessed as per the schedule in Appendix 1. Clinical laboratory results must be reviewed before the start of each cycle.

All enrolled patients will undergo clinical assessments and standard laboratory tests both before and during the study. Safety assessments will include medical interviews, AE recording, physical examinations, laboratory indicators (hematology, biochemistry, etc.), and other evaluations. Patients will also be closely monitored for any signs or symptoms of autoimmune diseases and infections.

All AEs must be recorded from the first administration of the study drug (SAEs from the time of informed consent) until 30 days after the last dose (including chemotherapy), or until another anticancer therapy is initiated, whichever occurs first. Post-treatment, AEs deemed related to the study treatment will be followed until they resolve to baseline or ≤ Grade 1, or are deemed stable by the investigator, the patient is lost to follow-up, the patient withdraws consent, or the AE is determined to be unrelated to the study treatment or participation.

The investigator will record all drug-related SAEs occurring from treatment termination until the patient’s death, withdrawal of consent, or loss to follow-up (whichever comes first). Investigators are required to report all AEs, including those related to pregnancy.

The following sections outline anticipated potential safety issues in this study and measures to avoid or reduce such toxicities.

5.3 Adverse Events

5.3.1 Definitions and Reporting

An AE is any adverse and unexpected sign (including abnormal laboratory values), symptom, or disease (new onset or worsening of pre-existing conditions) that has a temporal relationship with the use of the study drug, regardless of whether it is considered related to the study drug.

Examples of AEs include:

- Worsening of chronic or intermittent pre-existing conditions, including increased severity, frequency of attacks, prolonged duration, and/or significantly worsened outcomes.

- Newly diagnosed diseases discovered or confirmed after administration of the study drug, even if the disease may have existed before the study started.

- Signs, symptoms, or clinical consequences suspected to be caused by drug interactions.

- Signs, symptoms, or clinical consequences suspected to be caused by an overdose of the study drug or concomitant medications (overdose itself should not be reported as an AE or SAE).

If an AE or SAE occurs, the investigator is responsible for reviewing all records related to the AE or SAE (e.g., hospital records, laboratory results, and diagnostic reports). The investigator will then record all relevant information related to the AE or SAE in the CRF.

5.3.2 Severity Assessment

The investigator is required to evaluate the severity of each reported AE and SAE during the study. AEs and SAEs should be assessed and graded according to NCI-CTCAE v5.0.

Toxicities not applicable to NCI-CTCAE grading will be graded based on the following definitions:

- Grade 1: Mild; asymptomatic or mild symptoms; clinical or diagnostic observations only; no intervention needed.

- Grade 2: Moderate; minimal local or noninvasive intervention indicated; limiting age-appropriate instrumental ADL.

- Grade 3: Severe or medically significant but not immediately life-threatening; hospitalization or prolongation of existing hospitalization indicated; disabling; limiting self-care ADL.

- Grade 4: Life-threatening consequences; urgent intervention indicated.

- Grade 5: Death related to AE.

Note: The terms "severe" and "serious" are not synonymous. Severity is a measure of the intensity of an event (e.g., specific AE grades: mild [Grade 1], moderate [Grade 2], severe [Grade 3], or life-threatening [Grade 4]), while seriousness is based on regulatory criteria.

5.3.3 Causality Assessment

The investigator is responsible for assessing the relationship between each AE or SAE and the study drug based on their best clinical judgment. Other causes, such as the natural history of the underlying disease, concomitant treatments, other risk factors, and the temporal relationship between the AE or SAE and the study drug, should be considered and investigated. The investigator should refer to the Tislelizumab Investigator’s Brochure to determine their assessment.

For each AE, causality should be classified as "related" or "unrelated". If an AE is judged to have a "reasonable possibility" (i.e., there are facts, evidence, or parameters suggesting a causal relationship) of being caused by the study drug, it should be classified as related. Several factors should be considered in this assessment, including:

- Temporal relationship between the AE and the administration of the study drug/procedure.

- Whether other potential causes have been identified.

- The mechanism of action of the study drug.

- Biological plausibility.

If any of the following criteria are met, the AE can be classified as "related" to the study drug, otherwise, it should be classified as unrelated:

- Clear evidence of a causal relationship with other contributory factors excluded.

- Evidence suggesting a causal relationship, unlikely to be due to other factors.

- Some evidence of a causal relationship (e.g., AE occurring within a reasonable time frame after drug administration), but other factors might also have contributed to the AE (such as the patient's clinical condition or other concomitant AEs).

5.3.4 Follow-up of Adverse Events

After submitting an initial AE or SAE report, the investigator must actively follow each patient and provide further information about the patient's condition to the sponsor (in IIT trials, the investigator).

For all ongoing AEs and SAEs recorded and identified during the last visit/contact, continue to review them in subsequent visits/contacts.

All AEs and SAEs should be followed until resolution, stabilization, or until considered chronic, with another reasonable explanation, the patient is lost to follow-up, or the patient withdraws consent. The investigator should ensure follow-up information includes various supplementary examinations that indicate the nature of the AE or SAE and/or its causal relationship. These examinations may include additional laboratory tests, histopathological examinations, imaging studies, or consultations with other healthcare professionals.

5.3.5 Clinically Significant Abnormal Laboratory Test Results

Abnormal laboratory test results judged by the investigator to be clinically significant (e.g., clinical biochemistry, complete blood count [CBC], coagulation, or urinalysis) or other abnormal assessment results (e.g., ECG, X-ray, or vital signs) will be recorded as AE or SAE. This includes baseline abnormalities that worsen significantly during the study. The investigator determines clinical significance. Generally, clinically significant abnormal laboratory test results or other abnormal assessment results are those that:

- Are associated with clinical signs or symptoms, or

- Require active medical intervention, or

- Lead to treatment interruption or discontinuation, or

- Require close monitoring and more frequent follow-up assessments, or

- Require further diagnostic evaluation.

5.4 Definition of Serious Adverse Events

An SAE is an adverse medical event occurring at any dose that:

- Results in death.

- Is life-threatening. Note: "Life-threatening" in the SAE definition refers to an event where the patient was at risk of death when the event occurred. It does not refer to an event that could have caused death if it had been more severe.

- Requires hospitalization or prolongs existing hospitalization. Note: Hospitalization generally means the patient is admitted (usually for at least one night) to a hospital or emergency room for observation and/or treatment that cannot be performed in an office or outpatient setting.

- Results in disability/incapacity. Note: "Disability" refers to a substantial disruption of a person’s ability to conduct normal life functions. This definition does not include relatively minor medical events such as headaches, nausea, vomiting, diarrhea, influenza, and accidental injuries (e.g., sprained ankle) that may interfere with or prevent daily life functions but do not result in significant disruption.

- Results in congenital anomaly/birth defect.

- Is considered an important medical event by the investigator based on medical judgment (e.g., events that may jeopardize the patient or require medical/surgical intervention to prevent one of the above outcomes).

The following are not considered SAEs:

- Hospitalization for elective treatment of a pre-existing condition that has not worsened.

- Hospitalization for social/convenience reasons.

- Scheduled treatment of the target disease of this study, including hospitalizations for infusions or for convenience.

5.5 Suspected Unexpected Serious Adverse Reactions (SUSAR)

A Suspected Unexpected Serious Adverse Reaction (SUSAR) is an adverse reaction that is both unexpected (i.e., not listed in the product's reference safety information [RSI]) and meets the criteria for a Serious Adverse Drug Reaction (SADR), with specificity or severity that is inconsistent with the Investigator's Brochure.

5.6 Time, Frequency, and Method of Recording Adverse Events (AE) and Serious Adverse Events (SAE)

5.6.1 Adverse Event Reporting Period

SAEs should be reported only after the informed consent form has been signed but before the administration of the study drug begins. After initiating study drug treatment, all AEs and SAEs, regardless of their relation to the study drug, must be reported until 30 days after the last administration of the study drug (including chemotherapy) or until the initiation of new anti-cancer treatment, whichever comes first.

5.6.2 Reporting Serious Adverse Events

Once the investigator determines that an AE meets the criteria for an SAE as defined in the protocol, it must be reported immediately (within 24 hours) to the Ethics Committee or the Sponsor (for IIT trials, the investigator) or designated personnel, as described in the table below:

| Table 3. Timeline and Documentation Methods for Reporting Serious Adverse Events (SAEs) | | | | |
| --- | --- | --- | --- | --- |
| Event Type | Initial Reporting Timeline | Documentation Method | Follow-up Reporting Timeline | Reporting Method |
| All SAEs | Within 24 hours of first knowledge of the SAE | SAE Report | As soon as possible | Send SAE Form or Pregnancy Form via email or fax |

5.6.3 Active Inquiry of Adverse Events

Investigators or designated personnel will actively inquire about AEs by asking patients the following standard questions:

- How are you feeling?

- Have you had any medical problems since the last visit?

- Have you started any new medications since the last visit?

5.6.4 Disease Progression

Disease progression, which is expected within the study population and serves as an efficacy endpoint (including fatal disease progression), should not be reported as an AE term. However, symptoms, signs, or clinical sequelae resulting from disease progression should be reported as AEs. For example, if a patient develops pleural effusion due to disease progression in the lungs, the event should be reported as "pleural effusion" rather than disease progression. If disease progression results in fatal multi-organ failure, the SAE should be reported as "multi-organ failure leading to death" instead of "fatal disease progression" or "death due to disease progression."

5.6.5 Death

Death is an outcome and is generally not considered an event. If the only available information is death with an unknown cause, report the event as "death," "unknown cause of death," or "unexplained death."

5.6.6 Pregnancy

If a female patient or a male patient's partner becomes pregnant during the study treatment or within 120 days after the last dose of tislelizumab or chemotherapy (whichever is later), a pregnancy report form must be completed and promptly submitted to the Ethics Committee and the Sponsor (for IIT trials, the investigator) to track the outcome. Information about the mother and child will be sent to the Sponsor (for IIT trials, the investigator). Generally, follow-up time does not exceed 6 to 8 weeks post-expected delivery date. Any premature termination of pregnancy must be reported.

Although pregnancy itself is not considered an AE, any pregnancy complications or medically necessary elective terminations of pregnancy will be recorded as AEs or SAEs. Spontaneous abortions, therapeutic abortions, and natural miscarriages should all be reported as SAEs. Similarly, any congenital anomalies or birth defects in infants exposed to the study drug must be recorded and reported as SAEs.

5.6.7 Evaluation and Recording of Immune-Related Adverse Events

Due to the potential for autoimmune diseases triggered by anti-PD-1 therapy, AEs deemed immune-related by the investigator (see Section 5.7.3) should be classified as irAEs and identified on the CRF AE page until 90 days post-treatment termination.

5.7 Management of Special Attention AEs

As a routine precaution, patients must be observed in a location equipped with resuscitation equipment and emergency medications for at least 60 minutes after receiving tislelizumab infusion on Day 1 of the first two cycles before administering chemotherapy. From the third cycle onwards, patients should be observed in a resuscitation-equipped area for at least 30 minutes. The management of infusion-related reactions, severe hypersensitivity reactions, and irAEs as per NCI-CTCAE standards is listed below.

5.7.1 Infusion-Related Reactions

Symptoms of infusion-related reactions include fever, chills/rigor, nausea, pruritus, angioedema, hypotension, headache, bronchospasm, urticaria, rash, vomiting, myalgia, dizziness, or hypertension. Severe reactions may include acute respiratory distress syndrome, myocardial infarction, ventricular fibrillation, and cardiogenic shock. Patients should be closely monitored for such reactions. If an infusion reaction occurs, the patient must be immediately transferred to an ICU or an equivalent setting and given appropriate medical treatment, including epinephrine, corticosteroids, intravenous antihistamines, bronchodilators, and oxygen.

Treatment adjustments for symptoms of infusion-related reactions caused by the study drug are outlined below:

| NCI-CTCAE Grade | Tislelizumab Treatment Adjustment |
| --- | --- |
| **Grade 1 – Mild**  Transient mild reaction; no need to interrupt infusion; no intervention required. | Reduce infusion rate by 50%. Monitor closely for any worsening. Administer medication as needed. Prior to subsequent infusions, pre-medicate and reduce the infusion rate. |
| **Grade 2 – Moderate**  Requires treatment or interruption of infusion but resolves quickly with symptomatic treatment (e.g., antihistamines, NSAIDs, analgesics, IV fluids); requires prophylactic medication for ≤ 24 hours. | Stop the infusion. Once the infusion-related reaction resolves or severity decreases to Grade 1, resume infusion at 50% of the previous rate. Monitor closely for any worsening. Administer appropriate medication as described below. Prior to subsequent infusions, pre-medicate and reduce the infusion rate. |
| **Grade 3 - Severe** rolonged duration (e.g., does not quickly resolve with symptomatic treatment and/or brief interruption of infusion); symptoms recur after initial improvement; requires hospitalization due to clinical sequelae. | Immediately stop the infusion. Administer appropriate medication as described below. Discontinue study drug treatment for the patient. |
| **Grade 4 - Life-threatening**  Life-threatening consequences; requires urgent intervention. | Immediately stop the infusion. Administer appropriate medication as described below. Discontinue study drug treatment for the patient. Hospitalization is recommended. |

If the infusion rate of tislelizumab is reduced by 50% or stopped due to an infusion-related reaction, all subsequent infusions should be at a low rate and require pre-medication. If the patient experiences another infusion-related reaction (≥ Grade 2) at the slower infusion rate, the infusion should be stopped, and treatment with tislelizumab terminated.

NCI-CTCAE Grade 1 or 2 Infusion Reaction: Appropriate medical treatment should be administered based on the type of reaction. This includes but is not limited to antihistamines (e.g., diphenhydramine or equivalent), antipyretics (e.g., acetaminophen or equivalent), oral or intravenous corticosteroids if deemed necessary, epinephrine, bronchodilators, and oxygen. In the next treatment cycle, oral antihistamines (e.g., diphenhydramine or equivalent) and antipyretics (e.g., acetaminophen or equivalent) should be administered prior to infusion, and patients should be closely monitored for clinical signs or symptoms of infusion reactions.

NCI-CTCAE Grade 3 or 4 Infusion Reaction: Immediate appropriate medical treatment should be given based on the type and severity of the reaction. This includes but is not limited to oral or intravenous antihistamines, antipyretics, corticosteroids, epinephrine, bronchodilators, and oxygen.

5.7.2 Severe Hypersensitivity Reactions and Flu-Like Symptoms

If a hypersensitivity reaction occurs, it must be treated according to the best feasible medical practice as described in the full guideline for the emergency treatment of anaphylaxis by the Resuscitation Council (UK) [43]. Patients are required to report any delayed reactions to the investigator immediately.

In cases of systemic anaphylaxis/anaphylactoid reactions (typically occurring within minutes of drug administration/contact with the antigen, characterized by respiratory distress, laryngeal edema, and/or severe bronchospasm; usually accompanied by vascular collapse or shock, with no prior dyspnea; skin manifestations such as itching and urticaria with/without edema; and gastrointestinal symptoms like nausea, vomiting, abdominal cramps, diarrhea), the infusion must be stopped immediately, and the patient withdrawn from the study.

If a hypersensitivity reaction is observed, the patient should receive an injection of epinephrine and infusion of dexamethasone, be connected to a monitor immediately, and ICU should be alerted if needed to transfer the patient when necessary.

To prevent flu-like symptoms, 25 mg indomethacin or an equivalent dose of a non-steroidal anti-inflammatory drug (i.e., 600 mg ibuprofen, 500 mg naproxen sodium) should be administered 2 hours before and 8 hours after each study drug infusion. The investigator may provide alternative treatment for fever (i.e., paracetamol) as deemed appropriate.

5.7.3 Immune-Related Adverse Events

Immune-related adverse events (irAEs) are of particular concern in this study. If any of the following events or similar incidents occur, the investigator should rule out other causes (e.g., concomitant medications, infectious diseases, metabolic conditions, toxins, disease progression, or other tumor origins) through appropriate diagnostic tests. These tests may include but are not limited to serological, immunological, and histological (biopsy) data. If other causes have been excluded, and the AE requires treatment with systemic steroids, other immunosuppressants, or endocrine therapy, and is consistent with an immune-mediated mechanism, it should be recorded as an irAE on the CRF AE page.

A list of potential irAEs is provided in the table below. All conditions similar to those listed should be evaluated in patients receiving tislelizumab to determine if they are immune-related.

Refer to the latest European Society for Medical Oncology (ESMO) and American Society of Clinical Oncology (ASCO) guidelines for recommendations on the diagnosis, evaluation, and management of irAEs [44, 45]. Common immune-related toxicities are detailed in Appendix 5. For any adverse events not listed in Appendix 5, refer to the ASCO Clinical Practice Guidelines [45] for further information on the diagnosis, evaluation, and management of immune-related toxicities.

| Immune-Related Adverse Events | |
| --- | --- |
| Affected Body System | Event |
| Skin (Mild - Common) | Pruritus or maculopapular rash; Vitiligo |
| Skin (Moderate) | Follicular or urticarial dermatitis; Erythema/lichen planus-like rash; Sweet’s syndrome |
| Skin (Severe - Rare) | Full-thickness necrolysis/Stevens-Johnson syndrome |
| Gastrointestinal | Colitis (including diarrhea with abdominal pain or endoscopic/imaging evidence of inflammation); Pancreatitis; Hepatitis; Elevated transaminases (ALT/AST); Intestinal perforation |
| Endocrine | Thyroiditis; Hypothyroidism; Hyperthyroidism; Hypophysitis with features of hypopituitarism (e.g., fatigue, weakness, weight gain); Insulin-dependent diabetes; Diabetic ketoacidosis; Adrenal insufficiency |
| Respiratory | Non-infectious pneumonitis/diffuse alveolitis |
| Ocular | Episcleritis; Conjunctivitis; Iritis/Uveitis |
| Neuromuscular | Arthritis; Arthralgia; Myalgia; Neurological disorders; Guillain-Barré syndrome; Aseptic meningitis; Myasthenia syndrome/myasthenia gravis; Meningoencephalitis; Myositis |
| Hematologic | Anemia; Leukopenia; Thrombocytopenia |
| Renal | Interstitial nephritis; Glomerulonephritis; Acute renal failure |
| Cardiac | Pericarditis; Myocarditis; Heart failure |

If toxicity does not resolve to ≤ Grade 1 within 12 weeks, treatment with the investigational drug should be discontinued in consultation with the sponsor (for IIT trials, the investigator). If the same or a higher severity of any event recurs after resumption of the investigational drug, treatment should be permanently discontinued.

5.7.4 Renal Dysfunction

Patients with moderate renal impairment (eGFR >30 mL/min/1.73 m² and <60 mL/min/1.73 m² estimated by the CKD-EPI formula) may be enrolled in this study. The following rules should be followed when managing irAEs with steroids:

- If serum creatinine is normal at baseline, refer to sections 7.7.3 and Appendix 5 for diagnosing and treating patients with abnormal renal laboratory values.

- If baseline serum creatinine is Grade 1 and rises to ≥ Grade 2 after starting tislelizumab treatment, refer to Appendix 5 for diagnosing and treating patients with abnormal renal laboratory values. Use Appendix 6 and the eGFR calculator link to check the estimated glomerular filtration rate (eGFR). If there is only a Grade 2 increase in serum creatinine, the study treatment may continue unless serum creatinine has increased by at least 50% from baseline or eGFR has dropped to below 20 mL/min/1.73 m².

- If baseline serum creatinine is Grade 2 and rises to ≥ Grade 3 after starting tislelizumab, refer to Appendix 5 for diagnosing and treating patients with abnormal renal laboratory values. If there is only a Grade 3 increase in serum creatinine, the study treatment should be paused until serum creatinine returns to baseline, and treatment can only be resumed after discussion with the medical monitor.

6. Statistical Methods and Sample Size Determination

6.1 Statistical Analysis

6.1.1 Analysis Sets

The Intent-to-Treat (ITT) analysis set includes all enrolled patients. This set will serve as the primary analysis set for all efficacy analyses, including the analysis of progression-free survival (PFS) and overall survival (OS) endpoints.

The Safety Analysis Set includes all patients who have received at least one dose of any study drug; this set will be used for safety analyses. Patients will be analyzed according to the treatment regimen they actually received.

6.1.2 Demographic and Baseline Characteristics

Demographic and baseline characteristics of the ITT analysis set will be summarized using descriptive statistics. Continuous variables will include age, weight, vital signs, and time since first cancer diagnosis; categorical variables will include histology, prior systemic therapy, prior radiotherapy, disease stage, PD-L1 expression, sex, ECOG PS, smoking status, and metastatic sites.

6.2 Safety and Feasibility Analysis

Safety will be evaluated by monitoring and recording all adverse events (AEs) graded according to NCI-CTCAE 5.0. Safety assessments will also use laboratory test results (e.g., hematology, clinical chemistry, urinalysis), vital signs, ECG, and physical examination findings. Safety will be defined as the frequency of serious adverse events occurring within 90 days after the last PD-1 drug administration before surgery, 30 days post-surgery, or before starting new treatment (whichever occurs first). Safety analysis will primarily use descriptive methods and percentages, with a tabulated description of AEs occurring in this trial.

Feasibility will be assessed by the successful completion of preoperative treatment and surgery without any extended treatment-related delays. Delayed treatment is defined as occurring >37 days from the scheduled Day 0 under the specified conditions.

6.3 Efficacy Analysis

1. Major Pathological Response Rate (MPR):

MPR is defined as the proportion of patients achieving major pathological response, with less than 10% of viable tumor cells observed in the resected tumor as assessed by pathological examination.

2. Objective Response Rate (ORR):

ORR is defined as the proportion of enrolled patients with measurable disease at baseline who achieve a complete response (CR) or partial response (PR) as determined by RECIST v1.1. Patients who do not undergo post-baseline assessment will be considered non-responders.

3. Disease-Free Survival (DFS):

DFS is defined as the time from curative surgery to the first documentation of disease progression (assessed by RECIST v1.1) or death from any cause, whichever occurs first. The median DFS will be estimated using the Kaplan-Meier method, and a Kaplan-Meier curve will be plotted.

4. 5-Year Overall Survival (5-y OS):

5-y OS is defined as the time from the start of treatment to death from any cause within 5 years. The median PFS will be estimated using the Kaplan-Meier method, and a Kaplan-Meier curve will be plotted.

5. Overall Survival (OS):

OS is defined as the time from enrollment to death from any cause. The median PFS will be estimated using the Kaplan-Meier method, and a Kaplan-Meier curve will be plotted.

6. R0 Resection Rate:

The R0 resection rate is the proportion of patients who achieve an R0 resection (negative surgical margins with no residual tumor on microscopic examination) among all patients undergoing surgery.

7. Health-Related Quality of Life (HRQoL):

HRQoL will be assessed using the Lung Cancer Hospital Patient Questionnaire (based on QLQ-C30 and QLQ-LC13). Descriptive statistics (mean, SD, median, and range) of post-baseline scores will be summarized, along with the mean change from baseline (with 95% CI). The proportion of patients with clinically significant changes in selected items and subscales will be calculated at each assessment time point. Analyses will only include patients with baseline assessments and at least one post-baseline assessment and will be summarized in the ITT analysis set.

6.4 Exploratory Efficacy Analysis

Exploration of potential predictive biomarkers in blood and tumor tissue, including analysis of tumor-infiltrating lymphocytes.

6.5 Sample Size Considerations

This trial is expected to enroll 15 patients.

7. Ethical and Legal Considerations

7.1 Ethical Standards

The study will be conducted by the principal investigator and study center, adhering fully to the ICH E6 Guideline for Good Clinical Practice, the principles of the Declaration of Helsinki, or the regulations of the country where the study is conducted, whichever provides the greatest protection for individual subjects. The study will comply with the ICH E2A Guideline (Clinical Safety Data Management: Definitions and Standards for Expedited Reporting).

7.2 Independent Ethics Committee (IEC) or Institutional Review Board (IRB)

Prior to the commencement of the study, this protocol, the Informed Consent Form (ICF), any patient information, and supporting documents must be submitted by the principal investigator to the IRB/IEC for review and approval. Additionally, any patient recruitment materials must also receive IRB/IEC approval.

The principal investigator is responsible for providing the IRB/IEC with written summaries of the study status according to their requirements, policies, and procedures. Investigators are also responsible for promptly informing the IRB/IEC of any protocol amendments.

7.3 Protocol Amendments

Any amendments to the protocol will be prepared by the sponsor (for IIT trials, the investigator). All protocol amendments must be submitted to the regulatory authorities and the IRB/IEC, along with the revised ICF sample if applicable. The sponsor (for IIT trials, the investigator) must obtain written approval from the regulatory authorities and the IRB/IEC, as well as the necessary approval from the study centers before implementing any changes, except when necessary to eliminate immediate hazards to patients or changes of an administrative or logistical nature (e.g., change in the sponsor's medical monitor or contact information).

7.4 Subject Information and Informed Consent

Subjects must be provided with key information about the study and the informed consent form. Prior to study initiation, the research staff must provide the subjects with the written approval from the Ethics Committee/IRB of the informed consent form and all other written information. The Ethics Committee/IRB approval letter and the approved subject information/informed consent form must be archived in the study documents.

Informed consent must be obtained before any specific study procedures are performed. The date of the subject's consent to participate in the study and the signing of the informed consent form should be recorded in the subject's corresponding files.

7.5 Patient and Data Confidentiality

All records pertaining to patient identity will be kept confidential and, to the extent permitted by relevant laws and/or regulations, these records will not be disclosed to the public.

Subjects' names will not be provided. Case report forms (CRFs) will only record subject numbers and initials. If subjects' names appear in any other documents (e.g., pathology reports), the copies of these documents must have the names redacted. Data stored electronically must comply with relevant data protection laws. When study results are published, subjects' identities will also be kept confidential.

Researchers will retain a list to identify subjects' records.

8. Study Monitoring

If patient confidentiality complies with local requirements, the responsible monitor (or designated personnel) will regularly contact and visit the investigators and will be allowed to inspect various trial records (CRFs and other related data) as required.

During the entire study, the monitor is responsible for regularly checking CRFs, verifying compliance with the protocol, and examining the completeness, consistency, and accuracy of the data entries. The monitor should have access to laboratory test reports and other patient records to verify the entries in the CRFs. The investigator (or their designated personnel) agrees to cooperate with the monitor to ensure that any issues discovered during these monitoring visits are resolved.

9. Data Handling and Record Keeping

9.1 Case Report Forms

For each enrolled patient, the CRF must be completed and signed by the principal investigator or a designated representative authorized by the principal investigator. This also applies to CRF records for patients who do not complete the trial (even during the screening period if CRF has been filled out). If a patient withdraws from the treatment study, the reason for withdrawal must be recorded in the CRF. If a patient withdraws from the study due to treatment-limiting adverse events, every effort should be made to thoroughly document the outcome.

All forms should be printed or recorded with indelible ink and must be clear and legible. Errors should be crossed out rather than erased, and corrected entries should be inserted with the investigator or their authorized representative signing and dating the correction.

9.2 Data Collection

Data required by the protocol will be entered into the CRFs.

Data collection in CRFs should follow the instructions described in the CRF completion guidelines. The investigator is ultimately responsible for the collection and reporting of all clinical data entered into the CRFs. The investigator must provide a signature in the CRFs to confirm their accuracy, authenticity, and completeness.

9.3 Archiving

Data entered into the CRFs must match the original records or be directly recorded in the CRFs. When data are directly recorded into the CRFs, the entries will be considered original data. The parameters of the original data must be validated, and the information source must be documented. Study documents and all original data must be retained until notified by the sponsor to destroy them.

9.4 Investigator's File/Document Retention

To ensure a complete record of the study's conduct, enabling subsequent verification of the study data, the investigator must retain comprehensive and accurate records during the study's conduct. These documents should be classified into two distinct categories: (1) investigator’s study files, and (2) clinical source documents for patients.

The investigator's study files include the protocol and amendments, IRB/IEC and governmental approval letters, sample informed consent forms, drug records, personnel resumes, authorization forms, and other relevant documents/communications.

Patients' clinical source documents (usually predefined before the project starts, recorded outside the CRF, and containing key efficacy/safety parameters) typically include inpatient/outpatient records, doctors' and nurses' notes, appointment books, original laboratory reports, ECGs, EEGs, X-rays, pathology and specialist assessment reports, signed informed consent forms, consultation letters, patient screening and recruitment forms.

9.5 Audits and Inspections

The investigator should be aware that, upon formal notification, they must prepare the relevant original records for inspection and provide access to qualified personnel from the drug research quality assurance department or their designated personnel, or inspectors from health authorities. Verification of data in the CRFs must be directly checked against the original records.

10. References

1.	Siegel, R.L., K.D. Miller, and A. Jemal, Cancerstatistics, 2019. CA	Cancer J Clin, 2019. 69(1): p. 7-34.

2.	陈万青, 张思维, 郑荣寿, 雷正龙, 李光琳, 邹小农, and 赵平, 中国肿瘤登记地区 2007 年肿瘤发病和死亡分析. 中国肿瘤, 2011. 20(3): p. 162-169.

3.	Chen, W., R. Zheng, P.D. Baade, S. Zhang, H. Zeng, F. Bray, A. Jemal, X.Q. Yu, and J. He, CancerstatisticsinChina,2015.CA Cancer J Clin, 2016. 66(2): p. 115-132.

4.	段纪俊, 严亚琼, 杨念念, 曾晶, 郑荣寿, 张思维, and 陈万青, 中国恶性肿瘤发病与死亡的国际比较分析. 中国医学前沿杂志（电子版）, 2016. 8(7): p. 17-23.

5.	Kalemkerian, G.P.	and	B.J. Schneider, Advances in Small Cell Lung Cancer. Hematol	Oncol Clin North Am, 2017. 31(1): p. 143-156.

6.	Kalemkerian, G.P., B.W. Loo, W. Akerley, et al, NCCN Guidelines In sights: Small Cell Lung Cancer, Version2. 2018. J Natl Compr Canc Netw, 2018. 16(10): p. 1171-1182.

7.	Schmid, S. and M. Fruh, Immune check point inhibitors and small cell lung cancer: what's new? J Thorac Dis, 2018. 10(Suppl 13): p. S1503-s1508.

8.	Pesch, B., B. Kendzia, P. Gustavsson, K.H.et al, Cigarette smoking and lung cancer—relative risk estimates for the major histological types from a pooled analysis of case-control studies. Int J Cancer, 2012. 131(5): p. 12101219.

9.	Varghese, A.M., M.F. Zakowski, H.A. Yu, H.H. et al, Small-cell lung cancers inpatients who never smoked cigarettes. J Thorac Oncol, 2014. 9(6): p. 892-896.

10.	Evans, W.K., F.A. Shepherd, R. Feld, D. et al, VP-16 and cisplatin	as	first-line therapy for small-cell lung cancer. J Clin Oncol, 1985. 3(11): p. 1471-1477.

11.	Einhorn, L.H., J. Crawford, R. Birch, G. Omura, D.H. Johnson, and F.A. Greco, Cisplatin plus etoposide consolidation following cyclophosphamide, doxorubicin, and vincristine in limited small-cell lung cancer. J Clin Oncol, 1988. 6(3): p. 451-456.

12.	Sundstrom, S., R.M. Bremnes, S. Kaasa, U et al, Cisplatin and etoposide regimen is superior to cyclophosphamide	epirubicin	andvincristine	regimen	in	small-cell	lung	cancer: J Clin Oncol, 2002. 20(24): p. 4665-4672.

13.	Warde, P. and D. Payne, Does thoracic irradiation improve survival and local control in limited-stage smallcell carcinoma of the lung? A meta-analysis. J Clin Oncol, 1992.10(6): p. 890-895.

14.	Takada, M., M. Fukuoka, M. Kawahara, et al, Phase III study of concurrent versus sequential thoracic radiotherapy in combination with cisplatin and etoposide for limited-stage small-cell lung cancer: results of the Japan Clinical Oncology Group Study 9104.J Clin Oncol, 2002. 20(14): p. 3054-3060.

15.	Murray, N., P. Coy, J.L. Pater, I. et al., Importance of timing for thoracic irradiation in the combined modality treatment of limited-stages mall-cell lung cancer. The National Cancer Institute of Canada Clinical Trials Group. J Clin Oncol, 1993. 11(2): p. 336-344.

16.	Jeremic, B., Y. Shibamoto, L. Acimovic, and S. Milisavljevic, Initial versus delayed accelerated hyperfractionated radiationtherapy and concurrent chemotherapy inl imited small-cell lung cancer: a randomized study. J Clin Oncol, 1997. 15(3): p. 893-900.

17.	Rudin, C.M., M.C. Pietanza, T.M. Bauer, N. Ready, D. Morgensztern, et al, Roval pituzumabtesirine, aDLL3 targetedantibody drugconjugate, in recurrent small-cell lung cancer: a first-in-human, first-in-class, open-label, phase 1 study.Lancet Oncol, 2017. 18(1): p. 42-51.

18.	Roviello, G., L. Zanotti, M.R. Cappelletti, A. et al, No Advantage in Survival With Targeted Therapiesas Maintenance in Patients With Limited and Extensive-Stage Small Cell Lung Cancer: A Literature-Based MetaAnalysis of Randomized Trials. Clin Lung Cancer, 2016. 17(5): p. 334-340.

19.	Arnold, A.M., L. Seymour, M. Smylie, K. et al, Phase II study of vandetanibor placebo in small-cell lung cancer patients after complete or partial response to induction chemotherapy with or without radiation therapy: National Cancer Institute of Canada Clinical Trials Group Study BR.20. J Clin Oncol, 2007. 25(27): p. 4278-4284. 20. Owonikoko, T.K., S.E. Dahlberg, S.A. Khan, D.E.et al, A phase 1 safety study of veliparib combined with cisplatin	and	etoposide	in	extensive	stage	small	cell	lung	cancer:	A	trial	of	the ECOG-ACRIN Cancer Research Group(E2511).Lung Cancer, 2015. 89(1): p. 66-70.

21.	Fox, W. and J.G. Scadding, Medical Research Council comparative trial of surgery and radiotherapy for primary treatment of small-cell edoroat-cell edcarcinoma of bronchus. Ten-year follow-up. Lancet, 1973. 2(7820): p. 63-65.

22.	Lad, T., S. Piantadosi, P. Thomas, D. Payne, J. Ruckdeschel, and G. Giaccone, A prospective randomized trial to determine the benefit of surgical resection of residual disease following response of small cell lung cancer to combination chemotherapy. Chest, 1994. 106(6 Suppl): p. 320s-323s.

23.	Schreiber, D., J. Rineer, J. Weedon, D. Vongtama, A. Wortham, A. Kim, P. Han, K. Choi, and M. Rotman, Survival outcomes with the use of surgery in limited-stage small cell lung cancer: should its rolebere-evaluated? Cancer, 2010. 116(5): p. 1350-1357.

24.	Luchtenborg, M., S.P. Riaz, E. Lim, R. Page, D.R. Baldwin, E. Jakobsen, P. Vedsted, M. Lind, M.D. Peake, A. Mellemgaard, J. Spicer, L. Lang-Lazdunski, and H. Moller, Survival of patients with small cell lung cancer undergoing	lung	resection	in	England	1998-2009. Thorax, 2014. 69(3): p. 269-273.

25.	Yu, J.B., R.H. Decker, F.C. Detterbeck, and L.D. Wilson, Surveillance epidemiology and end results evaluation of	the	role	of	surgery	for	stageI	small	cell	lung	cancer. J Thora Oncol, 2010. 5(2): p. 215-219.

26.	Weksler, B., K.S. Nason, M. Shende, R.J. Landreneau, and A. Pennathur, Surgical resection should beconsidered	for	stage	I	and	II	small	cell	carcinoma	of	the	lung.	Ann	Thorac	Surg, 2012. 94(3): p. 889-893.

27.	Yang, C.F., D.Y. Chan, P.J. Speicher, B.C. Gulack, X. Wang, M.G. Hartwig, M.W. Onaitis, B.C. Tong, T.A. D'Amico, M.F. Berry, and D.H. Harpole, Role of Adjuvant Therapy in a Population-Based Cohortof Patients With Early-Stage Small-Cell Lung Cancer. J Clin Oncol, 2016. 34(10): p. 1057-1064.

28.	Tsuchiya, R., K. Suzuki, Y. Ichinose, Y. Watanabe, T. Yasumitsu, N. Ishizuka, and H. Kato, Phase	II trial	of postoperative adjuvant	cisplatin	and	etoposide	in patients	with completely resected stageI-IIIa small cell lung cancer: the Japan Clinical Oncology Lung	Cancer	Study	Group	Trial (JCOG9101). J Thorac Cardiovasc Surg, 2005. 129(5): p. 977-983.

29.	Yang,	C.J.,	D.Y.	Chan,	S.A.	Shah,	B.A.	et	al.	Long-term	Survival	after surgery Compared With Concurrent Chemoradiation for Node-negative Small Cell Lung Cancer. Ann Surg, 2018. 268(6): p. 1105-1112.

30.	Fujimori, K., A. Yokoyama, Y. Kurita, and	M. Terashima, A pilot phase 2 study of surgical treatment after induction chemotherapy for resectable stage I to IIIA small cell lung cancer.Chest, 1997. 111(4): p. 1089-1093. 31. Veronesi, G., P. Scanagatta, F. Leo, T. De Pas, G. et al Adjuvant Surgery after Carboplatinand VP16 in Resectable Small Cell Lung Cancer. Journal of Thoracic Oncology, 2007. 2(2): p. 131-134.

32.	Reck, M., A. Luft, A. Szczesna, L. et al, Phase III Randomized Trial of Ipilimumab Plus Etoposide and Platinum Versus Placebo Plus Etoposid eand Platinum in Extensive-Stage Small-Cell Lung Cancer. J Clin Oncol, 2016. 34(31): p. 3740-3748.

33.	Gadgeel, S.M., N.A. Pennell, M.J. Fidler, B.et al, Phase II Study of Maintenance Pembrolizumab in	Patients with Extensive-Stage Small Cell Lung	Cancer (SCLC). J Thorac Oncol, 2018. 13(9): p. 13931399.

34.	Antonia, S.J., J.A. Lopez-Martin, J. Bendell, P.A. Ott, et al. Nivolumab alone and nivolumab plus ipilimumab in recurrent small-cell lung cancer (CheckMate032): amulticentre, open-label, phase1/2trial. Lancet Oncol, 2016.17(7): p. 883-895.

35.	Dunn, G.P., A.T. Bruce, H. Ikeda, L.J. Old, and R.D. Schreiber, Cancer immune editing:from immunosurveillance to tumor escape. Nat Immunol, 2002. 3(11): p. 991-998.

36.	Carter,	L.,	L.A.	Fouser,	J.	Jussif,	L.et	al.	PD-1:	PD-L	inhibitory pathway affects both CD4(+) and CD8(+) T cells and isovercome by IL-2.Eur J Immunol, 2002. 32(3): p. 634-643.

37.	Langer, C.J., S.M. Gadgeel, H. Borghaei, V.A.et al. Carboplatin and pemetrexed with	or	without pembrolizumab for advanced, non-squamous non-small-cell lung	cancer: a randomised, phase	2 cohort of	the open-label KEYNOTE-021study.Lancet Oncol, 2016. 17(11): p. 1497-1508.

38.	Yoshimura,	A.,	T.	Yamada,	A.	Miyagawa-Hayashino,	et	al.	Comparing three different	anti-PD-L1 antibodies for immunohistochemical evaluation of small cell lung cancer. Lung Cancer, 2019. 137: p. 108-112.

39.	Azuma, T., S. Yao, G. Zhu, A.S. Flies, S.J. Flies, and L. Chen, B7-H1 is a ubiquitous antiapoptotic receptor on cancer cells. Blood, 2008. 111(7): p. 3635-3643.

40.	Hirano, F., K. Kaneko, H. Tamura, H. Dong, S. Wang, M. Ichikawa, C. Rietz, D.B. Flies, J.S. Lau, G. Zhu, K. Tamada, and L. Chen, Blockade of B7-H1 and PD-1 by monoclonal antibodies potentiates cancer therapeutic immunity. Cancer Res, 2005. 65(3): p. 1089-1096.

41.	Labrijn, A.F., A.O. Buijsse, E.T. van den Bremer, A.Y. Verwilligen, W.K. Bleeker, S.J. Thorpe, J. Killestein, C.H. Polman, R.C. Aalberse, J. Schuurman, J.G. van de Winkel, and P.W. Parren, Therapeutic IgG4 antibodies engage in Fab-arm exchange with endogenous humanIgG4invivo.Nat Biotechnol, 2009. 27(8): p. 767-771.

42.	Zhang, T., X. Song, L. Xu, J. Ma, et al. The binding of an anti-PD-1 antibody to Fc gammaR Iotahasa profound impact on its biological functions. Cancer Immunol Immunother, 2018. 67(7): p. 1079-1090.

43.	Ott, P.A., E. Elez, S. Hiret, D.W. Kim, A. Morosky, S. Saraf, B. Piperdi, and J.M. Mehnert, thePhaseIbKEYNOTE-028Study.J Clin Oncol, 2017. 35(34): p. 3823-3829.

44.	Paz-Ares, L., M. Dvorkin, Y. Chen, N. Reinmuth, K.et al. Goldman, Durvalumab plus platinum-etoposide versus platinum-etoposide in first-line treatment of extensive-stage small-cell lung cancer (CASPIAN): a randomised, controlled, open-label, phase3 trial. Lancet, 2019. 394(10212): p. 1929-1939.

45.	Horn, L., A.S. Mansfield, A. Szczesna, L. Havel, et al. First-Line Atezolizumab plus Chemotherapy in Extensive-Stage Small-Cell Lung Cancer. N Engl J Med, 2018. 379(23): p. 2220-2229.

46.	Waqar, S.N. and D. Morgensztern, Treatment advances in small cell lung cancer(SCLC). Pharmacol Ther, 2017.

180: p. 16-23.

Appendix 1. Assessment Schedule

| Estimate | Screening Period^1^ | | Preoperative Treatment Period | | | | | | | | | Surgery | Adjuvant therapy | Safety Visits^2^ | Follow-up^3^ |
| --- | --- | --- | --- | --- | --- | --- | --- | --- | --- | --- | --- | --- | --- | --- | --- |
|  |  |  | Every 3 Weeks as 1 Cycle, for 4 Cycles | | | | | | | | |  |  |  |  |
|  |  |  | Neoadjuvant cycle 1 | | | Neoadjuvant cycle 2 | | | Neoadjuvant cycle 3-4 | | |  |  |  |  |
| Time Window (Days) | -42 | -7 | 1(±3) | 8(±3) | 21(±3) | 1(±3) | 8(±3) | 21(±3) | 1(±3) | 8(±3) | 21(±3) | - |  | 30±7 days |  |
| informed consent | X |  |  |  |  |  |  |  |  |  |  |  |  |  |  |
| Inclusion/Exclusion Criteria | X |  |  |  |  |  |  |  |  |  |  |  |  |  |  |
| Demographic Information/History | X |  |  |  |  |  |  |  |  |  |  |  |  |  |  |
| Vital Signs/Height and Weight | X |  | X |  |  | X |  |  | X |  |  |  | X | X |  |
| Physical Examination | X |  | X |  |  | X |  |  | X |  |  |  | X | X |  |
| ECOG Performance Status | X |  | X |  |  | X |  |  | X |  |  |  | X | X |  |
| 12-lead ECG | X |  |  |  |  |  |  |  |  |  |  |  | X |  |  |
| Adverse Events^4^ | X |  | X |  | X | X |  | X | X |  | X |  | X | X | X |
| Concomitant Medications and Treatments | X |  | X |  | X | X |  | X | X |  | X |  | X | X | X |
| Complete Blood Count (CBC) |  | X |  | X | X |  | X | X |  | X | X |  | X | X |  |
| Serum Biochemistry |  | X |  |  | X |  |  | X |  |  | X |  | X | X |  |
| Liver, Kidney Function and Electrolytes |  | X |  | X | X |  | X | X |  | X | X |  | X | X |  |
| Coagulation Function |  | X |  |  |  |  |  | X |  |  | X |  | X | X |  |
| Myocardial Enzymes |  | X |  |  | X |  |  | X |  |  | X |  | X | X |  |
|  |  |  |  |  |  |  |  |  |  |  |  |  |  |  |  |
| Thyroid Function | X |  |  |  | X |  |  | X |  |  | X |  | X | X |  |
|  |  |  |  |  |  |  |  |  |  |  |  |  |  |  |  |
| Urinalysis |  | X |  |  | X |  |  | X |  |  | X |  |  | X |  |
| Hepatitis Markers | X |  |  |  |  |  |  | X |  |  | X |  |  | X |  |
| HIV Test | X |  |  |  |  |  |  |  |  |  |  |  |  |  |  |
| Pregnancy Test ^5^ | X |  |  |  |  |  |  |  |  |  |  |  |  |  |  |
| Pulmonary Function Test | X |  |  |  |  |  |  | X |  |  | X |  |  | X |  |
| Tumor Assessment^6^ | X |  |  |  | X |  |  | X |  |  | X |  | X | X |  |
| Tislelizumab |  |  | X |  |  | X |  |  | X |  |  |  |  |  |  |
| Platinum + Etoposide |  |  | X |  |  | X |  |  | X |  |  |  | X |  |  |
| Surgical Records |  |  |  |  |  |  |  |  |  |  |  | X |  |  |  |
| Pathological Results |  |  |  |  |  |  |  |  |  |  |  | X |  |  |  |
| Peripheral Blood (Research Use, 10mL) | X |  | X |  |  | X |  |  | X |  |  |  | X |  |  |
| Quality of Life Score | X |  |  |  | X |  |  | X |  |  | X |  | X |  |  |
| Survival Status |  |  |  |  |  |  |  |  |  |  |  |  |  |  | X |

Note:

1. No study-specific assessments or procedures should be performed until after the written informed consent has been signed. Results from standard care assessments or tests conducted within 35 ± 7 days before enrollment can be used for screening evaluations, eliminating the need for repeat tests.

2. A safety follow-up visit is required 90 days (± 7 days) after the last dose of study treatment or 30 days post-surgery, or before the start of any new anticancer treatment, whichever occurs first.

3. After treatment completion, follow-up assessments will occur every 3 months (±14 days) for 2 years. If no recurrence or metastasis is observed within 2 years post-treatment, the patient will enter the survival follow-up phase with telephone check-ins every 3 months (±14 days). These check-ins will gather information on survival status, time to disease recurrence, date and cause of death if applicable, and details of any subsequent anticancer treatments. Follow-up will continue for all patients to gather survival status and subsequent anticancer treatment data unless the patient opts out.

4. Adverse events (AEs) and laboratory abnormalities will be graded according to NCI-CTCAE v5.0. The severity of all AEs will be assessed. Only serious adverse events (SAEs) should be reported if they occur after informed consent but before the administration of the study drug. After the administration of the study drug, all AEs and SAEs must be reported regardless of their relation to the study drug until 90 days after the last dose of the study drug (including chemotherapy) or 30 days post-surgery or the initiation of new anticancer treatment, whichever occurs first.

5. A urine or serum pregnancy test (for women of childbearing potential, including those with tubal ligation) must be conducted within 30 days before enrollment, and the results must be negative. If the urine pregnancy test is positive or ambiguous, a serum pregnancy test must be performed.

6. If radiological examinations were conducted before obtaining written informed consent and within 35 ± 7 days before enrollment, these results can be used without the need for repeat examinations. All measurable and evaluable lesions must be assessed and recorded during the screening visit. A head MRI may be required based on clinical judgment during the screening period (a CT scan may be performed if MRI is contraindicated or impractical). A bone scan or PET-CT may be conducted if clinically indicated.

7. Adjuvant therapy will consist of four cycles (every 3 weeks per cycle), with adjuvant radiotherapy as needed.

Appendix 2. ECOG Performance Status

| **Grade** | **Description** |
| --- | --- |
| 0 | Fully functional, able to perform all pre-illness activities without limitation. (Karnofsky score 90-100) |
| 1 | Severe physical activity limitations, but able to walk and perform light physical or sedentary work (e.g., housework and office work). (Karnofsky score 70-80) |
| 2 | Able to walk and fully care for oneself, but unable to perform any work activities. Bedridden or awake for more than 50% of the time. (Karnofsky score 50-60) |
| 3 | Limited ability to care for oneself. More than 50% of the awake time spent in bed or sitting. (Karnofsky score 30-40) |
| 4 | Totally disabled with loss of activity. No self-care ability. Completely bedridden or sitting. (Karnofsky score 10-20) |

Appendix 3. RECIST Guideline for Solid Tumors (Version 1.1)

Definitions

The evaluation of remission and progression in this trial will be conducted according to the international standards proposed by the RECIST Committee (Version 1.1). The RECIST criteria use only changes in the longest diameter of target lesions (one-dimensional measurement) for assessment.

Note: Lesions are classified as measurable or non-measurable based on the criteria provided below. The term “assessable” is not used to describe measurability, as it does not provide additional significance or accuracy.

Measurable Lesions

- Tumor Lesions: Must have at least one measurable diameter (record the largest diameter in the measurement plane), with the minimum length as follows:

- CT and MRI Scans: 10 mm (no less than twice the slice thickness, at least 10 mm)

- Clinical Measurement Tools: 10 mm (lesion located on the body surface)

- Chest X-ray: 20 mm (if the boundaries are clear and surrounded by aerated lungs)

- Malignant Lymph Nodes: When assessed by CT scan (preferably with slice thickness ≤5 mm), lymph nodes must have a short axis ≥15 mm to be considered pathologically enlarged and measurable. Only the short axis should be measured and tracked at baseline and follow-up.

Non-measurable Lesions

- All other lesions (or disease sites), including small lesions (with maximum diameter ≥10 mm and <15 mm by conventional measurement techniques or <10 mm by spiral CT scan), are considered non-measurable. Non-measurable lesions include: leptomeningeal disease, ascites, pleural/pericardial effusion, inflammatory breast disease, skin or lymphangitic lung involvement, and abdominal masses/organ enlargement that cannot be measured reproducibly by imaging techniques.

- Bone Lesions:

- Bone scans, PET scans, or X-rays are not suitable for measuring bone lesions but can be used to confirm the presence or absence of bone lesions.

- If lytic or lytic-blastic mixed lesions have identifiable soft tissue components that meet the criteria for measurability, they can be considered measurable if assessed by cross-sectional imaging such as CT or MRI.

- Blastic bone lesions are considered non-measurable lesions.

- Cystic Lesions:

- Lesions defined as simple cysts by radiological imaging criteria should not be classified as malignant lesions (neither measurable nor non-measurable).

- Cystic lesions considered metastatic and meeting the above criteria for measurability can be classified as measurable. However, if non-cystic lesions are present in the same patient, non-cystic lesions should be prioritized as target lesions.

- Previously Treated Lesions:

- Lesions located in areas previously treated with radiation or other localized regional treatments are generally not considered measurable unless there is clear evidence of progression. The study protocol should detail conditions under which such lesions may be considered measurable.

Target Lesions

All measurable lesions (up to 2 per organ, total of 5 lesions) should be identified as target lesions and recorded and measured at baseline. Target lesions should be selected based on their size (longest diameter) to represent all involved organs and should be reproducible in repeated measurements.

Lymph nodes are noteworthy because they are normal anatomical structures that can be seen on imaging even without tumor metastasis. Pathological nodes defined as measurable and recognized as target lesions must meet the CT scan short axis ≥15 mm criterion. The baseline sum is derived from the sum of the short axes of these nodes. The short axis is the diameter usually used by radiologists to determine if a node is affected by solid tumors. The size of nodes is generally reported in two-dimensional data in the imaging plane obtained (for CT scans, results are almost always in the axial plane; for MRI, planes may be transverse, sagittal, or coronal). The shorter diameter is reported. For example, a reported abdominal nodule of 20 mm × 30 mm has a short diameter of 20 mm and is classified as a malignant, measurable nodule. In this case, the measurement of 20 mm should be recorded. Other pathological nodes (short axis ≥10 mm but <15 mm) should be considered non-target lesions. Nodes with short axis <10 mm are considered non-pathological and should not be recorded or tracked.

Sum the diameters of all target lesions (for non-nodular lesions, use the longest diameter; for nodular lesions, use the short axis) and report this as the baseline diameter sum. If lymph nodes are included in the sum, only the short axis data should be included. The baseline diameter sum will be used as a reference for further characterization of any objective tumor response.

Non-target Lesions

All other lesions (or disease sites), including pathological lymph nodes, should be identified as non-target lesions and recorded at baseline. These lesions do not require measurement and should be recorded as “present,” “absent,” or in some cases, “definite progression” (details below). Additionally, in the case of multiple non-target lesions in the same organ, they may be recorded as, for example, “multiple enlarged pelvic lymph nodes” or “multiple liver metastases.”

Measurable Disease Assessment Guidelines

- All measurements should be recorded in metric units and measured with calipers during clinical assessments. All baseline assessments must be conducted as close to the start of treatment as possible, and no more than 4 weeks before treatment begins.

- During baseline and follow-up, the same assessment methods and techniques should be used to characterize each identified and reported lesion. All lesions must be evaluated by imaging techniques, except those that can only be assessed by clinical examination.

- Clinical Lesions: Only superficial lesions with a diameter ≥10 mm (e.g., skin nodules) are considered for clinical measurement using calipers. Skin lesions should be documented with color photographs including a scale for measuring the lesion size. As mentioned, when lesions can be assessed by both clinical examination and imaging, imaging should be used due to its objectivity and ability to retrospectively review study endpoints.

- Chest X-ray: Chest CT is preferred over chest X-ray, especially when disease progression is a significant endpoint, as CT is more sensitive, particularly in detecting new lesions. However, clearly defined lesions surrounded by aerated lungs can also be detected by chest X-ray.

- CT, MRI: CT is currently the most effective and reproducible method for lesion assessment. This guideline specifies whether CT scans can be used based on slice thickness ≤5 mm. If the slice thickness exceeds 5 mm, measurable lesions must be at least twice the slice thickness. MRI can also be used in some cases (e.g., whole-body scans).

- Ultrasound: Ultrasound is not suitable for assessing lesion size and should not be used as a measurement method. Due to its operator-dependence and inability to reproducibly assess in subsequent dates, it does not guarantee consistency between measurements. If new lesions are detected using ultrasound during the trial, confirmation by CT or MRI is required. MRI can be used instead of CT if there are concerns about radiation exposure.

- Endoscopy, Laparoscopy: These techniques are not recommended for objective tumor assessment. However, they can be used to confirm complete pathological remission when obtaining biopsy samples or to confirm recurrence in studies where the endpoint is complete remission (CR) or recurrence after surgical resection.

- Tumor Markers: Tumor markers alone cannot evaluate objective tumor response. If markers are elevated above normal limits at baseline, they must return to standard levels when determining CR. Tumor marker measurements should be included in the protocol, considering their disease specificity. Specific guidelines for CA-125 response (for recurrent ovarian cancer) and prostate-specific antigen response (for recurrent prostate cancer) have been published. Additionally, the Gynecologic Oncology International Group has established CA-125 progression criteria to be used in combination with objective tumor assessment for first-line ovarian cancer trials.

- Cytology, Histology: These techniques may be used in rare cases as required by the protocol to distinguish between PR and CR (e.g., residual disease in germ cell tumors, where residual benign tumors may be present). When exudates are known potential side effects of treatment (e.g., certain taxanes or anti-angiogenic agents), cytology can confirm the tumor origin of any exudates appearing or worsening if measurable tumors meet the criteria for effective or stable disease.

Target Lesion Efficacy Evaluation

- Complete Response (CR): All target lesions disappear. Any pathological lymph nodes (whether target lesions or not) must decrease to a short axis <10 mm.

- Partial Response (PR): The sum of the diameters of target lesions decreases by at least 30% compared to baseline.

- Disease Progression (PD): The sum of the diameters of target lesions increases by at least 20% (including baseline sum if it is the smallest diameter in the study). In addition to a relative increase of 20%, the sum must also increase by at least 5 mm. (Note: The appearance of one or more new lesions is also considered disease progression).

- Stable Disease (SD): The decrease does not meet the criteria for PR, and the increase does not meet the criteria for PD.

- Lymph Nodes: Lymph nodes designated as target lesions must always have a recorded short-axis measurement (taken in the same anatomical plane as at baseline), even if the lymph nodes shrink to less than 10 mm during the study. This implies that if a lymph node is included as a target lesion, the "sum" of lesions cannot be zero, even when a Complete Response (CR) is achieved, as normal lymph nodes are defined as having a short axis of <10 mm. Case report forms should separately record that, to meet CR criteria, the short axis of each node must be <10 mm. For Partial Response (PR), Stable Disease (SD), and Progressive Disease (PD), the actual short-axis measurements of the nodes will be included in the sum of target lesions.

- Target Lesions That Become "Too Small to Measure": All lesions (both nodal and non-nodal) recorded at baseline should have their actual measurements recorded at each subsequent assessment, even if they become very small (e.g., 2 mm). However, sometimes lesions or lymph nodes recorded as target lesions at baseline may become indistinct on CT scans during the study, making it challenging for radiologists to measure them precisely. Such lesions might be reported as "too small to measure."

In such cases, it is important to record the measurements in the electronic Case Report Form (eCRF). If the radiologist believes the lesion might have disappeared, the measurement should be recorded as 0 mm. If the lesion is thought to be present but is too small to measure, a default value of 5 mm should be specified (Note: This rule is less likely to apply to lymph nodes, as they usually have a definable size and are often surrounded by fat, such as in the retroperitoneum; however, if a lymph node is believed to be present but too small to measure, a default value of 5 mm should be specified in such cases). The default value is based on the 5 mm slice thickness of CT scans (if this thickness changes, the default value should not be altered). This default value helps avoid erroneous conclusions about remission or disease progression due to measurement errors. However, if the radiologist can make an actual measurement, it should be recorded, even if the value is less than 5 mm.

- Lesions That Split or Fuse During Treatment: If non-nodal lesions are "dispersed," the longest diameter of the dispersed portions should be added together to calculate the sum of the target lesions' diameters. Similarly, if lesions fuse, they may maintain a plane between them, which aids in measuring the longest diameter of each lesion. If lesions have completely fused and are no longer separable, the longest diameter in the sagittal plane should be considered the longest diameter of the "fused lesion."

Although some non-target lesions may be measurable, measurement is not required; only qualitative assessment at the specified time points is mandated by the protocol.

- Complete Response (CR): All non-target lesions have disappeared, and tumor marker levels have returned to normal. All lymph nodes must meet non-pathological criteria in size (i.e., short axis <10 mm).

- Progressive Disease (PD): Clear progression of existing non-target lesions (details below). (Note: The appearance of one or more new lesions is also considered disease progression.)

- Non-CR/Non-PD: One or more non-target lesions persist and/or tumor marker levels remain above normal limits.

- When the Patient Has Measurable Lesions: In such cases, to achieve "clear progression" based on non-target disease, there must be substantial deterioration in non-target disease such that even with SD or PR in target disease, the overall tumor burden has increased to the extent that treatment should be stopped. A slight "increase" in one or more non-target lesions is generally insufficient to be categorized as clear progression. Therefore, specifying overall progression based solely on changes in target disease in such situations is extremely rare.

- When the Patient Has Only Non-Measurable Lesions: This situation occurs in some phase III trials where measurable lesions are not included in the inclusion criteria. The same general concept applies here, but without assessing measurable lesions to explain the increased burden of non-measurable lesions. Since deterioration of non-target lesions is difficult to quantify (by definition: if all lesions are indeed non-measurable), when assessing whether there is clear progression, if the change in non-measurable lesions indicates that the overall disease burden has increased to the extent required to determine PD in measurable lesions, a useful check can be applied: a 73% increase in tumor burden (equivalent to a 20% increase in measurable lesion diameter).

- Examples include: pleural effusion increasing from "trace" to "large," lymphangitic spread from localized to disseminated, or descriptions in the protocol as "requiring treatment adjustment." If clear progression is observed, the patient should be classified as having overall PD at that time. While applying objective criteria to non-measurable lesions is ideal, it is impractical due to the nature of the disease; hence, the increase must be substantial.

- New Lesions:

The appearance of new malignant lesions indicates disease progression; therefore, certain comments on the identification of new lesions are crucial. There are no specific criteria for identifying new radiological lesions; however, new lesions should be unequivocally identified, meaning they are not attributable to differences in scan techniques or changes in imaging patterns, nor are they due to non-tumor factors (e.g., some "new" bone lesions might merely represent healing or worsening of existing lesions). This is particularly important when baseline lesions show partial or complete response. For example, necrosis in liver lesions might be reported as "new cystic lesions" on a CT scan but not actually be new.

If a lesion in an anatomical location not detected at baseline is identified in subsequent follow-up tests, it is considered a new lesion, indicating disease progression. For instance, if organ disease is present at baseline, and a metastatic lesion is found in the brain via CT or MRI during the study, this brain metastasis can be considered evidence of PD, even though brain imaging was not performed at baseline.

If a new lesion is ambiguous, such as being too small to clearly define, continued treatment and follow-up evaluations are necessary to determine whether it represents a true new lesion. If repeated scans confirm a new lesion, the date of disease progression should be declared based on the initial scan date.

If FDG-PET efficacy evaluation is necessary, using FDG-PET scans to assist CT scans during progression assessments is sometimes reasonable (especially when "new" lesions might appear). New lesions based on FDG-PET imaging can be identified as follows:

- If FDG-PET was negative at baseline and positive on follow-up, this indicates new lesions, implying disease progression.

- If FDG-PET was not conducted at baseline and positive on follow-up: If the positive FDG-PET result correlates with new lesions confirmed by CT, PD can be determined. If the FDG-PET positive result is not confirmed as new lesions by CT, further CT scans are required to confirm whether progression has indeed occurred (if so, the date of FDG-PET abnormality is the date of PD determination). If the positive FDG-PET result correlates with lesions already identified by CT and there is no progression according to anatomical images, this is considered non-PD.

- Best Overall Response Evaluation:

Best overall response refers to the best response recorded from the start of the study treatment to the end of treatment, considering various factors. Sometimes the best response is recorded only after treatment completion; thus, the protocol should specify whether post-treatment evaluations should be considered when determining the best overall response. The protocol must state how starting any new treatment before disease progression will affect the best response evaluation outcome. The patient's best overall response will depend on the findings of target and non-target lesions, as well as any new lesions. Additionally, confirmatory measurements might be required depending on the trial's nature and protocol requirements. Especially in non-randomized trials where efficacy is the primary endpoint, confirming PR or CR is essential to identify the "best overall response."

Once all patient data is available, the best overall response can be determined. In trials where confirmation of complete or partial response is not required, the best response is defined as the best response observed at any time point (e.g., a patient assessed as SD at the first evaluation, PR at the second, and PD at the last evaluation will have a best overall response of PR). If disease stability is determined as the best response, it must also meet the minimum time requirement from baseline specified in the protocol. If the SD duration does not meet the minimum duration requirement but is indeed the best response at a time point, the patient's best response will depend on subsequent evaluations. For instance, if a patient is assessed as SD at the first evaluation and PD at the second, and the minimum SD duration is not met, the best response will be PD. If the patient is lost to follow-up after the first SD evaluation, the response is considered unevaluable.

| Target Lesion | Non-Target Lesion | New Lesion | Overall Response |
| --- | --- | --- | --- |
| CR | CR | No | CR |
| CR | Non-CR/Non-PD | No | PR |
| CR | Not Assessable | No | PR |
| PR | Non-progressive or Not Fully Assessable | No | PR |
| SD | Non-progressive or Not Fully Assessable | No | SD |
| Not Fully Assessable | Non-progressive | No | NE |
| PD | Any situation | Yes or no | PD |
| Any situation | PD | Yes or no | PD |
| Any situation | Any situation | Yes | PD |

Abbreviations: CR = Complete Response; NE = Not Evaluated; PD = Progressive Disease; PR = Partial Response; SD = Stable Disease.

If a nodal lesion is included in the sum of target lesions and the node’s size decreases to a “normal” size (<10 mm), the measurements should still be reported during scanning. Even if these nodes have returned to normal, the measurements should be recorded, and disease progression should not be overestimated based solely on whether the size of the node has increased. As mentioned, this means that the sum for a patient achieving CR might not be "zero."

In trials where efficacy needs to be confirmed, repeated “NE” time-point assessments may complicate the determination of best response. The trial’s analysis plan must specify how missing data/evaluations will be handled when assessing efficacy and disease progression. For example, in most trials, it is reasonable to confirm PR-NE-PR as a valid response when considering efficacy at different time points.

For patients whose overall health deteriorates and requires treatment interruption but who lack objective evidence of disease progression at that time point, the case should be reported as “symptomatic deterioration.” Even after treatment discontinuation, every effort should be made to record objective progression. Symptomatic deterioration is not a description of objective response; it is the reason for stopping trial treatment.

The conditions for defining “early progression, early death, and in-evaluability” are specific to each trial and should be clearly described in each protocol (depending on treatment duration and cycle settings).

In some cases, distinguishing residual disease from normal tissue may be challenging. If the assessment of complete response relies on this judgment, it is advisable to perform additional tests (e.g., fine-needle aspiration/biopsy) to confirm complete response before declaring it. FDG-PET can be used to upgrade the response assessment to CR in a manner similar to using biopsy to confirm residual radiological abnormalities as fibrosis or scarring. The use of FDG-PET in such cases should be pre-specified in the protocol and supported by disease-specific medical literature. However, it must be acknowledged that the limitations of FDG-PET and biopsy in terms of resolution and sensitivity may lead to false-positive conclusions of CR.

For ambiguous findings of disease progression (e.g., very small and uncertain new lesions; cystic changes and necrosis in existing lesions), treatment may continue until the next scheduled assessment. If progression is confirmed at the next scheduled assessment, the progression date should be recorded as earlier than the suspected progression date.

- Confirmatory Measurement/Duration of Response Confirmation

In non-randomized trials where response is the primary endpoint, it is necessary to discern whether the response is due to measurement error when confirming PR or CR. This also allows for appropriate interpretation of results based on historical data, as confirmation is traditionally required for such trials. However, in all other scenarios, such as randomized trials (Phase II or III) or trials where disease stability or progression is the primary endpoint, confirmation of efficacy may not be required as it does not add interpretive value. Nevertheless, eliminating the requirement for efficacy confirmation may make central review to prevent biases more critical, especially in non-blinded trials.

For SD, there must be at least one measurement meeting the SD criteria within the minimum time interval after entering the trial (usually no less than 6 weeks).

- Overall Duration of Response

The overall duration of response is measured from the time the CR/PR criteria are first met (based on the first recorded instance) to the first day of objective documentation of relapse or progressive disease (using the minimum recorded value during the study as the reference for disease progression).

The measurement of the total duration of complete response is from the time CR criteria are first met until the date of the first objective evidence of disease relapse.

- Duration of Stable Disease

The duration of stable disease is measured from the start of treatment (from the randomization date in randomized trials) until the standard for disease progression, using the minimum total recorded during the study as the reference (if the baseline total is the minimum, it should be used as the reference for calculating disease progression).

The clinical relevance of the duration of stable disease varies among different studies. If the proportion of patients achieving stable disease within the shortest time is an important endpoint in a particular trial, the protocol should specify the minimum time interval required for two determinations of stable disease.

- Note: The frequency of follow-up assessments after baseline evaluation can impact the duration of response, duration of stable disease, and progression-free survival. Defining standard follow-up frequencies is beyond the scope of this guideline. When determining frequency, multiple parameters should be considered, including disease type and stage, treatment cycle, and standard operating procedures. However, if comparing across different trials, the accuracy of the measured endpoints must be considered within these constraints.

Appendix 4. New York Heart Association (NYHA) Functional Classification

| New York Heart Association (NYHA) Functional Classification | |
| --- | --- |
| I | The patient has heart disease but does not experience any limitation in physical activity. Ordinary physical activity does not cause undue fatigue, palpitations, dyspnea, or angina. |
| II | The patient’s heart disease causes mild limitation in physical activity. No discomfort at rest. Mild physical activity results in fatigue, palpitations, dyspnea, or angina. |
| III | The patient’s heart disease causes marked limitation in physical activity. No discomfort at rest. Mild physical activity results in fatigue, palpitations, dyspnea, or angina. |
| IV | The patient’s heart disease prevents any physical activity. Symptoms of heart failure or angina may occur even at rest. Any physical activity increases discomfort. |

Appendix 5. Assessment and Management of Immune-Related Adverse Events

The following guidelines for the diagnosis and management of immune-related adverse events (irAEs) are provided as recommendations only. This document should be used in conjunction with clinical judgment from experts (specialists experienced in using immunotherapy for cancer) and institutional guidelines or policies.

Standards for diagnosing irAEs include blood tests, diagnostic imaging, tissue pathology, and microbiological assessments to exclude other causes such as infections, disease progression, and adverse reactions from concomitant medications. In addition to these tests, the following factors should be considered when diagnosing irAEs:

•	Is there a temporal relationship between the occurrence of the irAE and the administration of the immunotherapy (e.g., atezolizumab)?

•	How does the patient respond after discontinuation of the immunotherapy?

•	Does the event recur after resuming the immunotherapy?

•	Does corticosteroid treatment lead to clinical improvement?

•	Is the event indicative of autoimmune endocrine disease?

•	Is disease progression or another diagnosis a more likely cause?

After excluding other explanations for autoimmune toxicity, the irAE should be recorded in the Case Report Form (CRF).

| Recommended Diagnostic Tests for Immune-Related Adverse Events | |
| --- | --- |
| Immune-Related Toxicity | Diagnostic Assessment Guidelines |
| Thyroid Disease | Scheduled and repeated thyroid function tests (TSH and T4). |
| Pituitary Inflammation | Check visual fields and consider testing pituitary endocrine axis blood levels. Patients with headaches, visual disturbances, unexplained fatigue, weakness, weight loss, and unexplained systemic symptoms should undergo pituitary and brain MRI. If abnormalities are found, consult an endocrinologist. |
| Non-Infectious Pneumonia | All patients with new or worsening pulmonary symptoms or signs (e.g., upper respiratory infection, new cough, shortness of breath, or hypoxia) should be evaluated with high-resolution CT. Consider pulmonary function tests including DLCO. Radiologic images are often non-specific. Depending on the location of abnormalities, consider bronchoscopy and bronchoalveolar lavage or lung biopsy. Consult a pulmonologist if the cause is unclear. |
| Neurotoxicity | Perform a comprehensive neurological examination and brain MRI for all CNS symptoms; review alcohol history and other medications. Screen for diabetes, and assess blood B12/folate, HIV status, TFT, and consider autoimmune serology. For peripheral neuropathy, consider brain/spinal MRI/MRA and nerve conduction studies. Consult a neurologist if abnormalities are found. |
| Colitis | Review dietary intake to exclude fatty diarrhea. Consider a comprehensive assessment including FBC, UEC, LFTs, CRP, TFT, stool microscopy and culture, viral PCR, C. difficile toxin, and Cryptosporidium (resistant organisms). In cases of abdominal discomfort, consider imaging such as X-ray or CT scan. If bleeding, pain, or distension occurs, consider colonoscopy, biopsy, and surgical intervention as needed. |
| Ophthalmic Disorders | If the patient experiences acute, new, or worsening eye inflammation, blurred vision, or other visual disturbances, urgently refer to an ophthalmologist for evaluation and management. |
| Hepatitis | Check ALT/AST/total bilirubin, INR/albumin; frequency depends on AE severity (e.g., daily for grade 3-4, every 2-3 days for grade 2 until recovery). Review medications (e.g., statins, antibiotics) and alcohol history. Perform liver screening including hepatitis A/B serology, hepatitis E PCR, and assess anti-ANA/SMA/LKM/SLA/LP/LCI, iron levels. Consider imaging such as ultrasound to detect metastases or thromboembolism. Consult a hepatologist and consider liver biopsy. |
| Renal Toxicity | Assess fluid status and medication history. Perform urine analysis and culture. Consider renal ultrasound, protein assessment (urine dipstick/24-hour urine collection), or phase-contrast microscopy. Seek nephrology consultation for further management assistance. |
| Dermatology | Consider other causes through physical examination; if needed, perform skin biopsy and refer to dermatology. |
| Arthritis or Myositis | Review musculoskeletal history and complete a musculoskeletal examination. Consider joint X-rays and other imaging as needed to rule out metastatic disease. Perform autoimmune serology and consult rheumatology for additional management support. For suspected myositis/rhabdomyolysis/muscle weakness, include tests such as CK, ESR, CRP, troponin, and consider muscle biopsy. |
| Myocarditis | Perform ECG, echocardiogram, CK/CK-MB, troponin (I and/or T) tests, and refer to a cardiologist. |

Abbreviations: AE = Adverse Event; ALT = Alanine Aminotransferase; ANA = Antinuclear Antibody; AST = Aspartate Aminotransferase; CK = Creatine Kinase; CK-MB = Creatine Kinase-MB; CNS = Central Nervous System; CRP = C-Reactive Protein; CT = Computed Tomography; DLCO = Diffusing Capacity of the Lungs for Carbon Monoxide; ECG = Electrocardiogram; ESR = Erythrocyte Sedimentation Rate; FBC = Full Blood Count; HIV = Human Immunodeficiency Virus; INR = International Normalized Ratio; LCI = Liver Cystolic Antigen; LFT = Liver Function Tests; LKM = Liver-Kidney Microsomal Antibody; LP = Liver-Pancreas Antigen; MRA = Magnetic Resonance Angiography; MRI = Magnetic Resonance Imaging; PCR = Polymerase Chain Reaction; SLA = Soluble Liver Antigen; SMA = Smooth Muscle Antibody; T4 = Thyroxine; TFT = Thyroid Function Tests; TSH = Thyroid-Stimulating Hormone; UEC = Urea, Electrolytes, and Creatinine.

Treatment of Immune-Related Adverse Events

•	Immune-related adverse events can develop rapidly; therefore, research treatment may need to be interrupted based on the specific situation, with close monitoring and timely diagnostic testing and therapeutic intervention.

•	Immune-related AEs should rapidly improve following immunosuppressive therapy. If there is no improvement, reassess the diagnosis, seek further expert advice, and consult with the study medical monitor.

•	For participants who experience a Grade 3 immune-related adverse event but recover quickly, and if there is evidence of clinical response to the research treatment, consideration may be given to resuming the study drug therapy in consultation with the study medical monitor.

•	The steroid doses in the table below refer to oral or intravenous (methyl)prednisolone dosages. These can be substituted with equivalent doses of other corticosteroids. For steroid-resistant irAEs, consider using steroid-sparing agents such as mycophenolate mofetil (MMF).

•	If a patient requires long-term immunosuppressive therapy, consider using prophylactic antibiotics to prevent opportunistic infections.

| Autoimmune Toxicity | Grade | Treatment Guidelines (Based on Clinical Judgment) | Study Drug Management |
| --- | --- | --- | --- |
| Thyroid Disease | 1-2  Asymptomatic TFT Abnormalities or Mild Symptoms | If hypothyroidism, replace thyroid hormone until TSH/T4 levels normalize. If hyperthyroidism, refer to an endocrinologist. For systemic symptoms: pause the study treatment, use β-blockers, consider oral prednisone 0.5 mg/kg/day for thyroid pain. Gradually taper corticosteroids over 2-4 weeks. Monitor thyroid function to determine the need for hormone replacement therapy. | Continue study treatment; if systemic symptoms occur, pause study treatment. |
|  | 3-4  Severe Symptoms: Requires hospitalization. | Consult an endocrinologist. If hypothyroidism, replace with thyroid hormone 0.5-1.6 μg/kg/day (start with 0.5 μg/kg/day for elderly or patients with comorbidities). Add oral prednisone 0.5 mg/kg/day for thyroid pain. Hyperthyroidism patients need β-blockers and may require antithyroid drugs until thyroiditis resolves. | Pause study treatment; if symptoms improve to grade 0-1, treatment may be resumed. |
| Pituitary Inflammation | 1-2  Mild to Moderate Symptoms. | Refer to an endocrinologist for hormone replacement therapy. For pituitary inflammation, add oral prednisone 0.5-1 mg/kg/day. Taper corticosteroids gradually over at least 1 month. If no improvement within 48 hours, escalate to grade 3-4 management. | Continue study treatment. |
|  | 3-4  evere or Life-Threatening Symptoms. | Refer to an endocrinologist for evaluation and treatment. Administer 1 mg/kg intravenous methylprednisolone for headache/visual disturbances caused by pituitary inflammation. Switch to oral prednisone and taper gradually over at least 1 month. Maintain hormone replacement therapy as per endocrinologist’s recommendations. | For patients with headache/visual disturbances due to pituitary inflammation, pause study treatment until the event resolves or improves to grade 2 or lower. Termination of treatment is generally not required. |
| Non-Infectious Pneumonia | 1  Radiographic Changes Only. | Monitor symptoms every 2-3 days. If worsening occurs, escalate to grade 2 management. | Consider pausing study treatment until symptoms improve and the cause is determined. |
|  | 2  Symptomatic: Exertional Dyspnea. | If infection is suspected, start antibiotics. If symptoms/signs persist for 48 hours or worsen, add oral prednisone 1 mg/kg/day. Consider prophylactic treatment for Pneumocystis jirovecii pneumonia. Taper corticosteroids gradually over at least 6 weeks. Consider preventive measures for corticosteroid side effects, such as blood glucose monitoring and vitamin D/calcium supplements. | Pause study treatment. If symptoms fully resolve or are controlled with prednisone ≤10 mg/day, treatment may be resumed. If symptoms persist despite corticosteroid therapy, terminate study treatment. |
|  | 3-4  Severe or Life-Threatening Symptoms: Resting Dyspnea. | Provide inpatient treatment, starting with intravenous methylprednisolone 2-4 mg/kg/day. If symptoms do not improve or worsen after 48 hours, add 5 mg/kg infliximab (if no liver involvement). Switch to oral prednisone and taper gradually over at least 2 months. Administer broad-spectrum antibiotics as needed and consider prophylactic measures for Pneumocystis jirovecii pneumonia and other corticosteroid side effects, such as blood glucose monitoring and vitamin D/calcium supplements. | Terminate study treatment. |
| Neurotoxicity | 1  Mild Symptoms. | - | Continue study treatment. |
|  | 2  Moderate Symptoms. | Administer oral prednisone 0.5-1 mg/kg/day. Taper gradually over at least 4 weeks. Consult with a neurologist. | Pause study treatment; if symptoms improve to grade 0-1, treatment may be resumed. |
|  | 3-4  Severe/Life-Threatening Symptoms. | Based on symptoms, start with oral prednisone or intravenous methylprednisolone 1-2 mg/kg/day. Taper corticosteroids gradually over at least 4 weeks. If no improvement within 72-96 hours, consider azathioprine, MMF, or cyclosporine. | Terminate study treatment. |
| Colitis/Diarrhea | 1  Mild Symptoms: Compared to baseline, ≤3 watery stools per day and feeling well | Symptomatic management: rehydration, loperamide, and avoidance of high-fiber/lactose diets. If grade 1 persists for more than 14 days, manage as a grade 2 event. | Continue study treatment.。 |
|  | 2  Moderate Symptoms: Compared to baseline, 4-6 watery stools per day, or abdominal pain, or blood in stools, or nausea, or nighttime symptoms | Administer oral prednisone 0.5 mg/kg/day (not enteric-coated). Start treatment immediately without waiting for diagnostic results. Taper corticosteroids over 2-4 weeks. If symptoms recur during tapering, consider endoscopy. | Pause study treatment; if symptoms resolve or improve to baseline level, treatment may be resumed. |
|  | 3  Severe Symptoms: Compared to baseline, >6 watery stools per day, or symptoms within 1 hour after meals | Initiate intravenous methylprednisolone 1-2 mg/kg/day. Switch to oral prednisone and taper over at least 4 weeks. Consider preventive measures for corticosteroid side effects, such as blood glucose monitoring and vitamin D/calcium supplements. | Pause study treatment; if symptoms resolve or improve to baseline level and after discussion with the study medical monitor, treatment may be resumed. |
|  | 4  Life-Threatening Symptoms. | Symptoms worsen, and if perforation, sepsis, tuberculosis, hepatitis, or NYHA III/IV CHF have not occurred, consider infliximab 5 mg/kg or other immunosuppressants such as MMF or tacrolimus. Consult with a gastroenterologist for colonoscopy/sigmoidoscopy. | Terminate study treatment. |
| Skin Reactions | 1  Rash with or without symptoms, <10% BSA | Avoid skin irritation and sun exposure; consider topical emollients. | Continue study treatment. |
|  | 2  Rash covering 10%-30% BSA | Avoid skin irritation and sun exposure; consider topical emollients. Use topical steroids (medium-potency cream once daily or high-potency cream twice daily) ± oral or topical antihistamines for itching. Consider short-term oral steroids. | Continue study treatment. |
|  | 3  Rash covering >30% BSA, or grade 2 event with significant symptoms | Avoid skin irritation and sun exposure; consider topical emollients. Based on clinical judgment, initiate steroids as follows: Moderate symptoms: Oral prednisone 0.5-1 mg/kg/day for 3 days, then taper over 2-4 weeks. Severe symptoms: Intravenous methylprednisolone 0.5-1 mg/kg/day; switch to oral prednisone and taper over at least 4 weeks. | Pause study treatment. If AE resolves or improves to grade 1-2 rash, treatment may be resumed after discussion with the study medical monitor. |
|  | 4  Skin necrosis >30% BSA, with associated symptoms (e.g., erythema, purpura, epidermal peeling) | Administer intravenous methylprednisolone 1-2 mg/kg/day. Switch to oral prednisone and taper over at least 4 weeks. Hospitalize and request an urgent dermatology consultation. | Terminate study treatment. |
| Hepatitis | 1  ALT or AST > ULN to 3×ULN | Check LFT within 1 week and before the next dose to confirm no worsening. If LFT worsens, recheck every 48-72 hours until improvement is observed. | If LFT is unchanged or improves, continue study treatment. If LFT worsens, pause study treatment until improvement is observed. |
|  | 2  ALT or AST 3-5×ULN | Recheck LFT every 48-72 hours: For persistent ALT/AST elevation: consider oral prednisone 0.5-1 mg/kg/day for 3 days, then taper over 2-4 weeks. For increasing ALT/AST: initiate oral prednisone 1 mg/kg/day, taper over 2-4 weeks; if LFT worsens, increase the dose based on clinical judgment. | Pause study treatment; resume when event resolves/improves to baseline and taper prednisone to ≤10 mg/day. |
|  | 3  ALT or AST 5-20×ULN | Recheck LFT every 48-72 hours: For persistent ALT/AST elevation: consider oral prednisone 0.5-1 mg/kg/day for 3 days, then taper over 2-4 weeks. For increasing ALT/AST: initiate oral prednisone 1 mg/kg/day, taper over 2-4 weeks; if LFT worsens, increase the dose based on clinical judgment. | Pause study treatment until improvement to baseline; resume after discussion with the study medical monitor. |
|  | 4  ALT or AST > 20×ULN | ALT/AST < 400 IU/L and normal bilirubin/INR/albumin: initiate oral prednisone 1 mg/kg/day, taper over at least 4 weeks. ALT/AST > 400 IU/L or elevated bilirubin/INR/low albumin: initiate IV (methyl)prednisolone 2 mg/kg/day. When LFT improves to grade 2 or below, switch to oral prednisone and taper over at least 4 weeks. | Terminate study treatment. |
|  | If liver function deteriorates despite steroid therapy:  - If on oral prednisone: Switch to IV methylprednisolone pulse therapy.  - If on IV steroids: Add mycophenolate mofetil (MMF), 500-1000 mg twice daily.  - If deterioration continues with MMF: Consider adding tacrolimus.  The duration and dosage of continued steroid therapy will depend on the severity of the event. | | |
| Nephritis | 1  Creatinine 1.5× baseline or >ULN to 1.5× ULN | Monitor creatinine weekly. If symptoms worsen, manage according to the criteria below. | Continue study treatment. |
|  | 2  Creatinine >1.5×-3× baseline or >1.5×-3× ULN | Ensure adequate hydration within 48-72 hours and recheck creatinine. If no improvement, consider a 24-hour urine collection to measure creatinine clearance. Consult a nephrologist to discuss the need for a biopsy. If caused by the study drug, initiate oral prednisone 0.5-1 mg/kg, taper over at least 2 weeks. Monitor creatinine/U&E every 48-72 hours. | Pause study treatment. If not drug-induced, resume treatment. If drug-induced, resume study drug once the event resolves/improves to baseline. If prednisone is tapered to <10 mg, resume the study drug. |
|  | 3  Creatinine >3× baseline or >3×-6× ULN | Hospitalize the patient for monitoring and fluid balance restoration; recheck creatinine every 24 hours. Consult a nephrologist and discuss the need for a biopsy. If symptoms worsen, initiate IV (methyl)prednisolone 1-2 mg/kg. Taper corticosteroids over at least 4 weeks. | Pause study treatment until the cause is identified. If suspected drug-induced: discontinue study treatment. |
|  | 4  Creatinine >6× ULN | Manage according to grade 3 criteria in a hospital capable of providing renal replacement therapy. | Terminate study treatment. |
| Diabetes/Hyperglycemia | 1  Fasting glucose value >ULN up to 160 mg/dL (8.9 mmol/L) | Closely monitor and manage according to guidelines. Check C-peptide, anti-GAD, and islet cell antibodies. | Continue study treatment. |
|  | 2  Fasting glucose value 160-250 mg/dL (8.9-13.9 mmol/L) | Recheck glucose at least weekly. Manage according to clinical guidelines. | Continue study treatment; if hyperglycemia worsens, pause treatment. If glucose stabilizes at baseline or grade 0-1, resume treatment. |
|  | 3  Fasting glucose value 250-500 mg/dL (13.9-27.8 mmol/L) | Admit the patient and refer to a diabetologist for hyperglycemia management. Avoid corticosteroids as they may exacerbate hyperglycemia. | Pause study treatment until hyperglycemia resolves and glucose stabilizes at baseline or grade 0-1. |
|  | 4  Fasting glucose value >500 mg/dL (>27.8 mmol/L) | Admit the patient to the emergency department for diabetes management. Refer to a diabetes specialist for insulin maintenance therapy and monitoring. |  |
| Ocular Toxicity | 1  Asymptomatic ophthalmologic exam/diagnostic abnormalities | Consider other causes and provide topical treatment as needed. | Continue study treatment. |
|  | 2  Anterior uveitis or mild symptoms | Refer to an ophthalmologist for evaluation and topical corticosteroid treatment. Consider oral corticosteroid treatment. | Continue study treatment; if symptoms worsen or visual symptoms occur, pause treatment. |
|  | 3  Posterior uveitis/pan-uveitis or severe symptoms | Urgently refer to an ophthalmologist. Initiate oral prednisone 1-2 mg/kg, taper over at least 4 weeks. | Pause study treatment until improvement to grade 0-1; consult with the study medical monitor before resuming treatment. |
|  | 4  Blindness in the affected eye (at least 20/200) | Initiate IV (methyl)prednisolone 2 mg/kg/day. Switch to oral prednisone, taper over at least 4 weeks. | Terminate study treatment. |
| Pancreatitis | 1-2  Asymptomatic, abnormal blood test results | Monitor pancreatic enzymes. | Continue study treatment. |
|  | 3  Abdominal pain, nausea, and vomiting | Hospitalize for urgent care. Initiate IV (methyl)prednisolone 1-2 mg/kg/day. If amylase/lipase levels improve to grade 2, switch to oral prednisone, taper over at least 4 weeks. | Pause study treatment; resume only after discussion with the study medical monitor. |
|  | 4  Acute abdominal pain, surgical emergency | Hospitalize for urgent treatment and consult as clinically appropriate. | Terminate study treatment. |
| Arthritis | 1  Mild pain with inflammation and swelling | Manage according to clinical guidelines.。 | Continue study treatment.。 |
|  | 2  Moderate pain with inflammation and swelling, limited fine motor activity | Manage according to clinical guidelines. Consider referral to a rheumatologist. If symptoms worsen during treatment, manage as grade 3 event. | Continue treatment; if symptoms continue to worsen, pause study treatment until symptoms improve to baseline or grade 0-1. |
|  | 3  Severe pain with inflammation or permanent joint damage, limiting activities of daily living | Urgently refer to a rheumatologist for evaluation and treatment. Initiate oral prednisone 0.5-1 mg/kg, taper over at least 4 weeks. | Pause study treatment until improvement to grade 0-1; resume only after discussion with the study medical monitor. |
| Mucositis/Stomatitis | 1  Asymptomatic or mild symptoms | Consider local treatment or analgesia according to clinical guidelines. | Continue study treatment. |
|  | 2  Moderate pain, reduced oral intake, limited use of instruments | Treat according to clinical guidelines, including analgesics, local treatments, and oral hygiene care. Ensure adequate hydration. If symptoms worsen or if sepsis or bleeding occurs, manage as grade 3 event. | Continue study treatment. |
|  | 3  Severe pain, limited food and fluid intake, limited daily activities | Hospitalize for appropriate treatment. Initiate IV (methyl)prednisolone 1-2 mg/kg/day. If symptoms improve to grade 2, switch to oral prednisone and taper over at least 4 weeks. | Pause study treatment until improvement to grade 0-1. |
|  | 4  Life-threatening complications or dehydration | Hospitalize for urgent care. Consider IV corticosteroids if there are no contraindications for infection. | Terminate study treatment. |
| Myositis/Rhabdomyolysis | 1  Mild weakness with/without pain | Prescribe analgesics. If CK is significantly elevated and patient is symptomatic, consider oral steroids and manage as grade 2. | Continue study treatment. |
|  | 2  Moderate weakness with/without pain | If CK is ≥3×ULN, initiate oral prednisone 0.5-1 mg/kg and taper over at least 4 weeks. | Pause study treatment until improvement to grade 0-1. |
|  | 3-4  Severe weakness, limited self-care ability | Hospitalize and initiate oral prednisone 1 mg/kg. For severe limitations in mobility or difficulty swallowing, consider IV (methyl)prednisolone 1-2 mg/kg/day. If symptoms do not improve, add immunosuppressive therapy. Taper oral steroids over at least 4 weeks. | Pause study treatment until improvement to grade 0-1. If there are signs of myocardial involvement, discontinue study treatment. |
| Myocarditis | <2  Asymptomatic, but with significantly elevated CK-MB or troponin levels, or clinically significant intraventricular conduction delay | Conduct cardiac evaluation with close monitoring through serial serum testing; consider referral to a cardiologist. If myocarditis is diagnosed, manage according to grade 2 event guidelines. | Pause the study treatment.  If myocarditis is diagnosed, permanently discontinue study treatment for patients with moderate or severe symptoms. For asymptomatic or mildly symptomatic patients, resume treatment with the study drug only after cardiac parameters have returned to baseline and following discussion with the study medical monitor. |
|  | 2  Symptoms occur with mild to moderate exertion. | Hospitalize the patient and initiate treatment with oral prednisone or intravenous (methyl)prednisolone at a dose of 1-2 mg/kg/day. Consult a cardiologist and manage heart failure symptoms according to clinical guidelines. If there is no immediate improvement, switch to high-dose (methyl)prednisolone 1 g/day and consider adding MMF, infliximab, or anti-thymocyte globulin. |  |
|  | 3  Severe symptoms occur with minimal exertion. |  |  |
|  | 4  Life-Threatening |  |  |

Abbreviations: AE = Adverse Event; ALT = Alanine Aminotransferase; AST = Aspartate Aminotransferase; BSA = Body Surface Area; CHF = Congestive Heart Failure; CK = Creatine Kinase; CK-MB = Creatine Kinase-MB; INR = International Normalized Ratio; IV = Intravenous; LFT = Liver Function Tests; MMF = Mycophenolate Mofetil; NYHA = New York Heart Association; T4 = Thyroxine; TB = Tuberculosis; TFT = Thyroid Function Tests; TSH = Thyroid-Stimulating Hormone; U&E = Urea and Electrolytes; ULN = Upper Limit of Normal.

**Appendix 6. Chronic Kidney Disease Epidemiology Collaboration (CKD-EPI) Formula**

In adult patients, the most commonly used formulas to estimate glomerular filtration rate (GFR) based on serum creatinine levels are the Chronic Kidney Disease Epidemiology Collaboration (CKD-EPI) formula and the Modification of Diet in Renal Disease (MDRD) Study formula. The National Kidney Disease Education Program (NKDEP) calculator is based on creatinine results traceable to the isotope dilution mass spectrometry (IDMS) method. All laboratories should use creatinine measurement methods that are traceable to IDMS.

If serum creatinine (Scr) is reported in mg/dL, use the CKD-EPI formula calculator. This formula is recommended when the estimated GFR (eGFR) is >60 mL/min/1.73 m².

The CKD-EPI formula is:

GFR=141 × min (Scr/κ, 1) α × max (Scr/κ, 1)-1.209 × 0.993 Age × 1.018[if female] × 1.159[if Black]

where:

- Scr is serum creatinine in mg/dL,

- κ is 0.7 for females and 0.9 for males,

- α is -0.329 for females and -0.411 for males,

- min denotes the smaller of Scr/κ or 1,

- max denotes the larger of Scr/κ or 1.

This formula does not require weight data as the reported results are adjusted to a body surface area of 1.73 m², which is an acceptable average value for adult body surface area.

- CKD-EPI Online Calculator URL: [https://www.niddk.nih.gov/health-information/communication-programs/nkdep/laboratory-evaluation/glomerular-filtration-rate-calculators](https://www.niddk.nih.gov/health-information/communication-programs/nkdep/laboratory-evaluation/glomerular-filtration-rate-calculators)

References:

1. Levey AS, Stevens LA, Schmid CH, et al. A new equation to estimate glomerular filtration rate. Ann Intern Med. 2009;150(9):604-12.
